# Supplementary material for: Assembly and Functional Role of PACE Transporter PA2880 from Pseudomonas aeruginosa
Source: Microbiol Spectr. 2022 Apr 4;10(2):e01453-21. doi: 10.1128/spectrum.01453-21 (PMC9045395; doi:10.1128/spectrum.01453-21)
Supplement: SUPPLEMENTAL FILE 1 — Supplemental material. Download SPECTRUM01453-21_Supp_1_seq4.pdf, PDF file, 6.9 MB [file spectrum01453-21_supp_1_seq4.pdf]

Supplemental material

Assembly and functional role of the PACE transporter PA2880 from *Pseudomonas aeruginosa*

Jiangfeng Zhao[a,b], Nils Hellwig[c], Bardya Djahanschiri[d], Radhika Khera[b], Nina Morgner[c], Ingo Ebersberger[d,e,f], JingkangWang[a]# and Hartmut Michel[b]#

[a] Tianjin University, School of Chemical Engineering and Technology, State Key Laboratory for Chemical Engineering, Collaborative Innovation Center of Chemical Science and Chemical Engineering, 300072, Tianjin, P. R. China

[b] Department of Molecular Membrane Biology, Max Planck Institute of Biophysics, Max-von-Laue-Strasse 3, 60438, Frankfurt am Main, Germany

[c] Institute of Physical and Theoretical Chemistry, Goethe University Frankfurt, Max-von-Laue Strasse 7, 60438, Frankfurt am Main, Germany

[d] Department for Applied Bioinformatics, Institute for Cell Biology and Neuroscience, Goethe-University Frankfurt, Max-von-Laue Strasse 13, 60438, Frankfurt am Main, Germany

[e] Senckenberg Biodiversity and Climate Research Centre Frankfurt (BIK-F), Frankfurt am Main, 60325, Germany

[f] LOEWE Centre for Translational Biodiversity Genomics, Frankfurt am Main, 60325, Germany

#Address correspondence to: jkwang@tju.edu.cn, Hartmut.Michel@biophys.mpg.de

Supplementary Text

Our phylogenetic analysis has revealed that the Acinetobacter AceI clade forms a group of distantly related and diverged members of the PACE subfamily. The cumulative branch lengths connecting AceI with PA2880, i.e. the patristic distance between the two sequences, sums up to 2.49 substitutions per site. The patristic distance between AceI and the closest related sequence from outside the genus Acinetobacter in our analysis, a protein from the beta-proteobacterium *Herminiimonas arsenitoxidans*, is with 2.50 equally high. In turn, the patristic distance between PA2880 and its closest relative outside the genus, a sequence from the beta-proteobacteria, *Polaromonas naphthalenivorans* (Burkholderiales) is with 1.16 less than half this distance. We next tested whether the short length of the proteins interferes with an accurate reconstruction of their phylogenetic relationships. We repeated the phylogenetic analysis for the second PACE family transporter encoded in the *A. baumannii* genome, A1S_1503 (WP_001161759.1). The resulting tree features, as expected given the relationships of the bacterial species, a monophyletic clade harboring A1S_1503, its ortholog in *P. aeruginosa*, PA2757, alongside with other gamma-proteobacterial sequences (Supplementary figure S2). The patristic distance between A1S_1503 and PA2757 is with 0.65 substitutions per site substantially smaller than that observed for AceI and PA2880. Again, beta-proteobacterial sequences, mainly from the Burkholderiales, fall within the diversity of this clade. However, an analysis of the gene neighborhood revealed that the taxa that are placed in this clade exclusively share a microsyntenic region with *A. baumannii*, where a LysR-like transcriptional activator is placed in tail-to-tail orientation next to A1S_1503. This strongly suggests that the placement of the beta-proteobacterial taxa in this clade can be explained by two independent horizontal gene transfer events from a gamma-proteobacterial donor to a beta-proteobacterial acceptor, leaving the relationships between PA2757 and A1S_1503 unaffected. No such conservation of microsynteny is seen for the AceI clade (Supplementary data 1 and 2). Considering all evidences, we conclude that the phylogenetic signal in members of the PACE

family should, in principle, suffice to reconstruct their evolutionary relationships. In the case of AceI, the evolutionary scenario is more complex. It appears that repeated lateral transfers especially involving members of the Burkholderiales together with lineage specific adaptations resulting in evolutionarily derived members, as represented by the Acinetobacter AceI clade, have resulted in the complex and reticulated evolutionary history of this sub-family.

Supplementary table S1. List of PACE family proteins shown in supplementary figure S1. Proteins are listed in the order of PA2880 orthologs shown in the tree.

**PACE family protein NCBI ID**	***Taxon name***
**NP_251570.1**	***Pseudomonas aeruginosa* PAO1**
WP_003101714.1	*Pseudomonas aeruginosa* PAO1H2O
WP_151135081.1	*Pseudomonas lalkuanensis*
WP_004422384.1	*Pseudomonas furukawaii*
WP_026042082.1	*Pseudomonas mendocina* S5.2
WP_039964952.1	*Pseudomonas pseudoalcaligenes* CECT 5344
WP_106734668.1	*Pseudomonas sediminis*
WP_092377222.1	*Pseudomonas sihuiensis*
WP_169939679.1	*Pseudomonas multiresinivorans*
WP_160287669.1	*Pseudomonas knackmussii*
WP_043316109.1	*Pseudomonas citronellolis*
WP_127163557.1	*Entomomonas moraniae*
WP_168923752.1	*Polaromonas vacuolata*
WP_011801923.1	*Polaromonas naphthalenivorans* CJ2
WP_076199707.1	*Rhodoferax koreense*
WP_048394105.1	*Pseudomonas lini*
WP_092278963.1	*Pseudomonas prosekii*
WP_114884984.1	*Pseudomonas kribbensis*
WP_064383679.1	*Pseudomonas glycinae*
WP_090285475.1	*Pseudomonas granadensis*
WP_083368130.1	*Pseudomonas koreensis*
WP_064676219.1	*Pseudomonas silesiensis*
WP_042933029.1	*Pseudomonas mandelii* JR-1
WP_090187228.1	*Pseudomonas arsenicoxydans*
WP_020797064.1	*Pseudomonas umsongensis*
WP_075945721.1	*Pseudomonas reinekei*
WP_093215033.1	*Pseudomonas vancouverensis*
WP_011062813.1	*Pseudomonas protegens* CHA0
WP_009050361.1	*Pseudomonas chlororaphis*
WP_010445502.1	*Pseudomonas fuscovaginae*
WP_090205669.1	*Pseudomonas asplenii*
WP_010567056.1	*Pseudomonas extremaustralis*
WP_083357106.1	*Pseudomonas antarctica*
WP_079444562.1	*Pseudomonas veronii*
WP_076949665.1	*Pseudomonas cedrina*
WP_124433755.1	*Pseudomonas orientalis*
WP_084381041.1	*Pseudomonas mucidolens*
WP_063027768.1	*Pseudomonas yamanorum*
WP_032862591.1	*Pseudomonas brenneri*

WP_053257660.1	*Pseudomonas fluorescens*
WP_057023865.1	*Pseudomonas synxantha*
WP_003193689.1	*Pseudomonas lactis*
WP_071496625.1	*Pseudomonas azotoformans*
WP_057007095.1	*Pseudomonas trivialis*
WP_034100011.1	*Pseudomonas rhodesiae*
WP_034108949.1	*Pseudomonas lurida*
WP_071489489.1	*Pseudomonas extremorientalis*
WP_010208247.1	*Pseudomonas simiae*
WP_115433430.1	*Crenobacter cavernae*
WP_064808946.1	*Ralstonia insidiosa*
WP_045219569.1	*Ralstonia mannitolilytica*
WP_014618758.1	*Ralstonia solanacearum*
WP_011004760.1	*Ralstonia pseudosolanacearum*
WP_062117380.1	*Collimonas pratensis*
WP_061539502.1	*Collimonas fungivorans*
WP_061535372.1	*Collimonas arenae*
WP_071071668.1	*Cupriavidus malaysiensis*
WP_ 004522489.1	*Burkholderia pseudomallei*
WP_ 004186193.1	*Burkholderia mallei*
WP_ 009892379.1	*Burkholderia thailandensis* E264
WP_ 010102325.1	*Burkholderia oklahomensis* C6786
WP_ 059566347.1	*Burkholderia stagnalis*
WP_ 084904795.1	*Paraburkholderia acidophila*
WP_ 059752793.1	*Burkholderia ubonensis*
WP_ 006763744.1	*Burkholderia dolosa* AU0158
WP_ 006405647.1	*Burkholderia multivorans* ATCC BAA-247
WP_ 047898698.1	*Burkholderia pyrrocinia*
WP_ 034201481.1	*Burkholderia cenocepacia*
WP_ 059558163.1	*Burkholderia seminalis*
WP_ 069258043.1	*Burkholderia metallica*
WP_ 122168915.1	*Burkholderia stabilis*
WP_ 153488639.1	*Burkholderia cepacia*
WP_ 025260823.1	*Pseudomonas cichorii J*BC1
WP_ 088235573.1	*Pseudomonas viridiflava*
WP_ 149032149.1	*Pseudomonas syringae pv. tomato str.* DC3000
WP_ 005780561.1	*Pseudomonas amygdali pv. tabaci str.* ATCC 11528
WP_ 090121328.1	*Kosakonia arachidis*
WP_ 153688543.1	*Enterobacter cancerogenus*
WP_ 153742813.1	*Kluyvera intermedia*
WP_ 024908486.1	*Enterobacter asburiae*
WP_ 025756445.1	*Enterobacter sichuanensis*
WP_ 032640125.1	*Enterobacter chengduensis*
WP_ 038419443.1	*Enterobacter cloacae*

WP_ 008503221.1	*Enterobacter roggenkampii*
WP_ 034495154.1	*Buttiauxella agrestis*
WP_ 126356346.1	*Cedecea lapagei*
WP_ 061276462.1	*Cedecea neteri*
WP_ 013201087.1	*Erwinia billingiae* Eb661
WP_ 012442171.1	*ERWTA* 465817 1
WP_ 012668831.1	*Erwinia pyrifoliae*
WP_ 004156431.1	*Erwinia amylovora* CFBP1430
WP_ 160620112.1	*Mixta intestinalis*
WP_ 085067677.1	*Pantoea alhagi*
WP_ 104956577.1	*Mixta gaviniae*
WP_ 038627530.1	*Mixta calida*
WP_ 067428522.1	*Erwinia gerundensis*
WP_ 039338466.1	*Pantoea stewartii*
WP_ 014606419.1	*Pantoea ananatis* PA13
WP_ 013357032.1	*Pantoea vagans*
WP_ 140916006.1	*Pantoea eucalypti*
WP_ 124889752.1	*Pantoea agglomerans*
WP_ 015696336.1	*Rahnella aquatilis* CIP 78.65 = ATCC 33071
WP_ 086005804.1	*Rouxiella badensis*
WP_ 051124010.1	*Dickeya aquatica*
WP_ 050569394.1	*Dickeya poaceiphila*
WP_ 012770925.1	*Dickeya chrysanthemi* Ech1591
WP_ 023639018.1	*Dickeya zeae*
WP_ 024104771.1	*Dickeya dianthicola*
WP_ 038661798.1	*Dickeya fangzhongdai*
WP_ 022632335.1	*Dickeya solani* IPO 2222
WP_ 013316633.1	*Dickeya dadantii* 3937
WP_ 040344087.1	*Brenneria nigrifluens* DSM 30175 = ATCC 13028
WP_ 121514202.1	*Brenneria goodwinii*
WP_ 005971921.1	*Pectobacterium wasabiae* CFBP 3304
WP_ 012822743.1	*Pectobacterium parmentieri*
WP_ 107168219.1	*Pectobacterium punjabense*
WP_ 039290329.1	*Pectobacterium atrosepticum*
WP_ 039313974.1	*Pectobacterium brasiliense*
WP_ 010305875.1	*Pectobacterium carotovorum*
WP_161528182.1	*Pectobacterium odoriferum*
WP_103971852.1	*Pectobacterium versatile*
WP_039485099.1	*Pectobacterium polaris*
WP_024486100.1	*Serratia fonticola*
WP_157193182.1	*Pseudomonas alkylphenolica*
WP_015673251.1	*Serratia rubidaea*
WP_013813975.1	*Serratia plymuthica* AS9
WP_112363870.1	*Serratia quinivorans*

WP_061795696.1	*Serratia ficaria*
WP_095847870.1	*Gibbsiella quercinecans*
WP_004936357.1	*Serratia nematodiphila*
WP_019453822.1	*Serratia surfactantfaciens*
WP_049300106.1	*Serratia marcescens*
WP_004717931.1	*Yersinia ruckeri*
WP_064517058.1	*Yersinia entomophaga*
WP_004716894.1	*Yersinia rohdei*
WP_025384414.1	*Yersinia similis*
WP_002211616.1	*Yersinia pestis* A1122
WP_002211616.1	*Yersinia pseudotuberculosis*
WP_032905959.1	*Yersinia intermedia*
WP_042548125.1	*Yersinia aldovae* 670-83
WP_004878201.1	*Yersinia mollaretii* ATCC 43969
WP_019083648.1	*Yersinia enterocolitica*
WP_145554939.1	*Yersinia canariae*
WP_129197699.1	*Yersinia hibernica*
WP_087487672.1	*Tatumella citrea*
WP_114986174.1	*Pragia fontium*
WP_108902128.1	*Limnobaculum parvum*
WP_109912721.1	*Providencia rettgeri*
WP_068445857.1	*Providencia heimbachae*
WP_025800792.1	*Hafnia alvei*
WP_047370074.1	*Phytobacter ursingii*
WP_041851514.1	*Phytobacter diazotrophicus*
WP_112213987.1	*Klebsiella huaxiensis*
WP_025107400.1	*Klebsiella michiganensis*
WP_057213031.1	*Klebsiella quasipneumoniae*
YP_005228637.1	*Klebsiella pneumoniae subsp. pneumoniae* HS11286
WP_008806359.1	*Klebsiella variicola*
WP_115191973.1	*Raoultella terrigena*
WP_015369903.1	*Klebsiella aerogenes*
WP_141963565.1	*Raoultella electrica*
WP_032690866.1	*Raoultella planticola*
WP_104896877.1	*Raoultella ornithinolytica*
WP_062741211.1	*Lignolyticus*
WP_002443838.1	*Shimwellia blattae* DSM 4481 = NBRC 105725
WP_059307252.1	*Leclercia adecarboxylata*
WP_062773200.1	*Kluyvera intermedia*
WP_014071632.1	*Enterobacter soli*
WP_152082122.1	*Enterobacter oligotrophicus*
WP_104950175.1	*Lelliottia nimipressuralis*
WP_032643224.1	*Enterobacter chengduensis*
WP_014171480.1	*Enterobacter ludwigii*

WP_015960293.1	*Lelliottia amnigena*
WP_095283238.1	*Lelliottia jeotgali*
WP_061382392.1	*Citrobacter werkmanii*
WP_003840976.1	*Citrobacter freundii*
WP_008785723.1	*Citrobacter portucalensis*
WP_042999456.1	*Citrobacter amalonaticus*
WP_001163400.1	*Salmonella bongori*
WP_017465480.1	*Salmonella enterica subsp. enterica serovar Thompson*
WP_001530217.1	*Salmonella enterica subsp. Enterica*
WP_001519547.1	*Salmonella enterica subsp. enterica serovar Enteritidis*
WP_001519547.1	*Salmonella enterica subsp. enterica serovar Muenchen*
WP_001519547.1	*Salmonella enterica subsp. enterica serovar Enteritidis str.* RM2968
WP_001519547.1	*Salmonella enterica subsp. enterica serovar Typhimurium*
NP_461938.1	*Salmonella enterica subsp. enterica serovar Typhimurium str.* LT2
WP 092283621.1	*Pseudomonas sabulinigri*
WP_180307888.1	*Chitinibacter fontanus*
WP_159875580.1	*Aquitalea denitrificans*
WP_019101823.1	*Chromobacterium haemolyticum*
WP_019101824.1	*Chromobacterium haemolyticum*
WP_149295449.1	*Chromobacterium paludis*
WP_114063097.1	*Chromobacterium phragmitis*
WP_011136228.1	*Chromobacterium violaceum* ATCC 12472
WP_046157682.1	*Chromobacterium vaccinii*
WP_067750013.1	*Orrella dioscoreae*
WP_043680601.1	*Castellaniella defragrans* 65Phen
WP_010929261.1	*Bordetella parapertussis*
WP_003814711.1	*Bordetella bronchiseptica*
WP_005018628.1	*Bordetella holmesii*
WP_043209783.1	*Bordetella pseudohinzii*
WP_029580220.1	*Bordetella hinzii*
WP_149065184.1	*Achromobacter insolitus*
WP_062681238.1	*Achromobacter denitrificans*
WP_006388706.1	*Achromobacter xylosoxidans*
WP_100853153.1	*Achromobacter spanius*
WP_137166478.1	*Salinimonas lutimaris*
WP 137166478.1	*Salinimonas lutimaris*
WP_076592872.1	*Herminiimonas arsenitoxidans*
WP_113997181.1	*Acinetobacter haemolyticus*
WP_004701059.1	*Acinetobacter seifertii*
WP_002010078.1	*Acinetobacter baumannii*
WP_002010078.1	*Acinetobacter nosocomialis* M2
**WP_002010078.1**	***Acinetobacter baumannii***
WP_171429638.1	*Acinetobacter lactucae*
WP_004643197.1	*Acinetobacter calcoaceticus*

YP_004995804.1	*Acinetobacter pittii* PHEA-2
WP_005073799.1	*Acinetobacter oleivorans* DR1
WP_099948838.1	*Acinetobacter junii*
WP 005189632.1	*Acinetobacter dispersus*
WP_153371643.1	*Acinetobacter wanghuae*
WP_004927292.1	*Acinetobacter baylyi* ADP1
WP_087512387.1	*Acinetobacter chinensis*
WP_065992038.1	*Acinetobacter defluvii*
WP_004822192.1	*Acinetobacter guillouiae*
WP_067559227.1	*Acinetobacter larvae*
WP_054581917.1	*Acinetobacter equi*
WP_166011976.1	*Acinetobacter shaoyimingii*
WP_106984915.1	*Acinetobacter cumulans*
WP_163165765.1	*Acinetobacter schindleri*
WP_166324890.1	*Acinetobacter lanii*
WP_038497828.1	*Basilea psittacipulmonis* DSM 24701
WP_014112181.1	*Taylorella asinigenitalis* MCE3
WP_015555498.1	*Taylorella equigenitalis*
WP_039123148.1	*Allofrancisella guangzhouensis*
WP_112870881.1	*Francisella adeliensis*
WP_072713391.1	*Francisella uliginis*
WP_071629719.1	*Francisella opportunistica*
WP_064460706.1	*Francisella persica* ATCC VR-331
WP_014548887.1	*Francisella hispaniensis*
WP_088821892.1	*Francisella philomiragia*
WP_159185050.1	*Francisella noatunensis subsp. noatunensis* FSC774
WP_041263637.1	*Francisella salina*
WP_041263637.1	*Francisella marina*
WP_010937615.1	*Desulfovibrio vulgaris* RCH1
WP _172961714.1	*Desulfovibrio ferrophilus*
WP_165191856.1	*Pseudohalocynthiibacter aestuariivivens*
WP_049834454.1	*Octadecabacter temperatus*
WP_055208895.1	*Rhodobacter capsulatus*
WP_119334274.1	*Hydrogenophilus thermoluteolus*
WP_108644250.1	*Rhodobacter blasticus*
WP_090194561.1	*Pseudomonas pohangensis*
WP_082396508.1	*Oryzomicrobium terrae*
WP_102042929.1	*Thauera hydrothermalis*
WP_011237746.1	*Aromatoleum aromaticum* EbN1
WP_157108220.1	*Azoarcus olearius*
WP_075149629.1	*Thauera chlorobenzoica*
WP_107222467.1	*Thauera aromatica* K172
WP_097011793.1	*Pseudodesulfovibrio profundus*
WP_015850467.1	*Desulfovibrio salexigens* DSM 2638

WP_041277522.1	*Desulfotalea psychrophila* LSv54
WP_088878983.1	*Vibrio mediterranei*
WP_004745074.1	*Vibrio tubiashii* ATCC 19109
WP_067385742.1	*Marinobacterium aestuarii*
WP_172534770.1	*Plesiomonas shigelloides*
WP_182788989.1	*Aeromonas media*
WP_021139191.1	*Aeromonas salmonicida*
WP_005335423.1	*Aeromonas veronii*
WP_197928974.1	*Aeromonas allosaccharophila*
WP_069362105.1	*Salinivibrio kushneri*
WP_089140410.1	*Vibrio rumoiensis*
WP_086980797.1	*Vibrio aphrogenes*
WP_019439568.1	*Moritella marina* ATCC 15381
WP_012550097.1	*Aliivibrio salmonicida* LFI1238
WP_025285946.1	*Granulibacter bethesdensis* CGDNIH4
WP_011279910.1	*Psychrobacter arcticus* 273-4
WP_094038524.1	*Zobellella denitrificans*
WP_087036922.1	*Oceanisphaera profunda*
WP_150992711.1	*Cupriavidus basilensis*
WP_063236873.1	*Cupriavidus oxalaticus*
WP_012351861.1	*Cupriavidus taiwanensis* LMG 19424
WP_010812186.1	*Cupriavidus necator* H16
WP_018008890.1	*Cupriavidus neocaledonicus*
WP_062796993.1	*Cupriavidus nantongensis*
WP_085377874.1	*Paracoccus contaminans*
WP_165192756.1	*Pseudohalocynthiibacter aestuariivivens*
WP_101283849.1	*Thalassospira marina*
WP_075777123.1	*Thioclava nitratireducens*
WP_024089704.1	*Leisingera methylohalidivorans* DSM 14336
WP_079552407.1	*Halomonas subglaciescola*
WP_138572356.1	*Paraoceanicella profunda*
WP_193082827.1	*Pseudopuniceibacterium antarcticum*
WP_148861870.1	*Marinobacter fonticola*
WP_048384468.1	*Marinobacter psychrophilus*
WP_075194959.1	*Marinobacter salarius*
WP_079551388.1	*Halomonas subglaciescola*
WP_014576160.1	*Marinobacter adhaerens* HP15
WP_070969857.1	*Marinobacter salinus*
WP_024313366.1	*Rhizobium favelukesii*
WP_038583541.1	*Neorhizobium galegae bv. orientalis str.* HAMBI 540
WP_099516135.1	*Microvirga ossetica*
WP_014129510.1	*Pelagibacterium halotolerans*
WP_018063714.1	*Martelella mediterranea* DSM 17316
WP_005619956.1	*Epibacterium mobile* F1926

WP_048535233.1	*Marinovum algicola* DG 898
WP_039000849.1	*Halocynthiibacter arcticus*
WP_013165162.1	*Starkeya novella* DSM 506
WP_099621955.1	*Caulobacter mirabilis*
WP_074966652.1	*Paracoccus aminovorans*
WP_010397736.1	*Paracoccus kondratievae*
WP_198140514.1	*Paracoccus denitrificans*
WP_099650356.1	*Paracoccus yeei*
WP_012092982.1	*Ochrobactrum anthropi*
WP_130188971.1	*Massilia lutea*
WP_131145343.1	*Massilia albidiflava*
WP_137313314.1	*Massilia umbonata*
WP_058987219.1	*Acetobacter senegalensis*
WP_197539949.1	*Acetobacter pasteurianus* 386B
WP_197687358.1	*Acetobacter oryzoeni*
WP_202878117.1	*Acetobacter oryzifermentans*
WP_208858880.1	*Acetobacter ascendens*
WP_136962973.1	*Phreatobacter stygius*
WP_109961943.1	*Methylobacterium terrae*
WP_012252728.1	*Methylorubrum extorquens* PA1
WP_175276521.1	*Oricola thermophila*
WP_005671962.1	*Lautropia mirabilis*
WP_005671968.1	*Lautropia mirabilis*
WP_013346671.1	*Ferrimonas balearica* DSM 9799
WP_044623377.1	*Photobacterium gaetbulicola* Gung47
WP_044555658.1	*Shewanella piezotolerans* WP3
WP_144210685.1	*Shewanella donghaensis*
WP_041421816.1	*Shewanella sediminis* HAW-EB3
WP_077754788.1	*Shewanella psychrophila*
WP_133407346.1	*Parashewanella tropica*
WP_127750774.1	*Parasedimentitalea marina*
WP_012600123.1	*Vibrio atlanticus*
WP_017057825.1	*Vibrio kanaloae*
WP_010431783.1	*Vibrio cyclitrophicus*
WP_025009453.1	*Shewanella algae*
WP_011864953.1	*Shewanella loihica* PV-4
WP_033538623.1	*Shewanella marisflavi*
WP_012141590.1	*Shewanella sediminis* HAW-EB3
WP_012323981.1	*Shewanella woodyi* ATCC 51908
WP_012154392.1	*Shewanella pealeana* ATCC 700345
WP_012276293.1	*Shewanella halifaxensis* HAW-EB4
WP_004725480.1	*Vibrio furnissii*
WP_020330256.1	*Vibrio fluvialis*
WP_033197397.1	*Vibrio qinghaiensis*

WP_013857051.1	*Vibrio anguillarum*
WP_124731043.1	*Shewanella livingstonensis*
WP_140234293.1	*Shewanella polaris*
WP_011637292.1	*Shewanella frigidimarina* NCIMB 400
WP_033020790.1	*Pseudoalteromonas paragorgicola* KMM 3548
WP_064664832.1	*Pseudoalteromonas prydzensis* ACAM 620
WP_011041283.1	*Colwellia psychrerythraea* 34H
WP_088875881.1	*Vibrio mediterranei*
WP_006086899.1	*Shewanella baltica* OS678
WP_004744309.1	*Vibrio tubiashii* ATCC 19109
WP_171322666.1	*Vibrio europaeus*
WP_158143952.1	*Vibrio metschnikovii*
WP_026060833.1	*Vibrio vulnificus*
WP_005390910.1	*Vibrio diabolicus*
WP_005390910.1	*Vibrio antiquarius*
WP_005462438.1	*Vibrio parahaemolyticus*
WP_009705768.1	*Vibrio owensii*
WP_005450737.1	*Vibrio harveyi*
WP_038867149.1	*Vibrio jasicida* 090810c
WP_128810645.1	*Vibrio alfacsensis*
WP_088880583.1	*Vibrio rotiferianus*
WP_062334438.1	*Moraxella osloensis*
WP_041640089.1	*Mannheimia succiniciproducens* MBEL55E
WP_006248618.1	*Mannheimia haemolytica* USMARC 2286
WP_025267441.1	*Bibersteinia trehalosi* USDA-ARS-USMARC-188
WP_005598619.1	*Actinobacillus pleuropneumoniae*
WP_039198466.1	*Actinobacillus equuli subsp. Equuli*
WP_081906454.1	*Actinobacillus suis* ATCC 33415
WP_080915056.1	*Shewanella japonica*
WP_102522486.1	*Vibrio tapetis subsp. Tapetis*
WP_012600935.1	*Vibrio atlanticus*
WP_010430503.1	*Vibrio cyclitrophicus*
WP_077681737.1	*Vibrio kanaloae*
WP_088878676.1	*Vibrio mediterranei*
WP_081149777.1	*Colwellia beringensis*
WP_144210570.1	*Shewanella donghaensis*
WP_011329659.1	*Pseudoalteromonas translucida*
WP_010552555.1	*Pseudoalteromonas arctica* A 37-1-2
WP_197709260.1	*Pseudoalteromonas carrageenovora*
WP_164504445.1	*Pseudoalteromonas espejiana* DSM 9414
WP_020195742.1	*Vibrio owensii*
WP_038868300.1	*Vibrio jasicida* 090810c
WP_065546680.1	*Vibrio scophthalmi*
WP_075650208.1	*Vibrio ponticus*

WP_162047704.1	*Vibrio taketomensis*
WP_106872465.1	*Campylobacter blaseri*
WP_108924313.1	*Actinobacillus porcitonsillarum*
WP_077663976.1	*Rodentibacter heylii*
WP_089035146.1	*Neisseria chenwenguii*
WP_123795531.1	*Neisseria animalis*
WP_067440059.1	*Eikenella exigua*
WP_003824472.1	*Eikenella corrodens*

Supplementary table S2: Drug resistance levels of *E. coli* cells expressing PA2880.

	Chlorhexidine (µg/ml)	Cadaverine (mg/ml)
**BW25113 *(ΔacrB, ΔmdfA, ΔemrE)***		
Control	0.25	1.25
PA2880-eGFP	1	1.25
PA2880	0.5	1.25
**BL21**		
Control	0.5	1.25
PA2880	1	1.25

Supplementary table S3: Binding affinity between protein and chlorhexidine.

	K_d value (μM)
PA2880-eGFP	2.15 ± 0.4
E38Q	2.69 ± 0.82
D83A	1.43 ± 0.42
H101A	1.86 ± 0.86
E106A	4.56 ± 0.34
D132A	1.79 ± 0.62
eGFP	–

–: No binding was detected.

Supplementary table S4: Salt composition of buffers used in eletrogenicity experiments.

KCl (mM)		Choline chloride (mM)		Membrane potential (mV)
Inside	Outside	Inside	Outside	
50	50/1/200	0/0/150	0/49/0	0/-100/+36

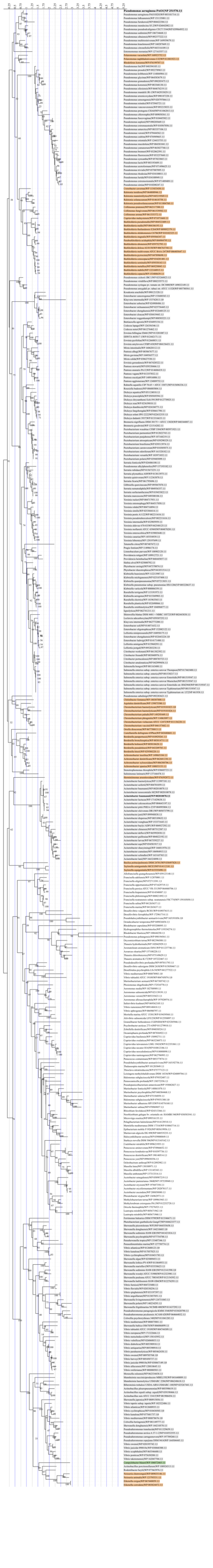

Supplementary figure S1. A complete representation of maximum likelihood phylogeny of 399 *A. baumannii* AceI orthologs. The two focal clades, *Acinetobacter spp.*, and *P. aeruginosa* are shown in bold face. The leaf label was written in the way of species name | RefSeq protein ID. The backgrounds of the node labels indicate the bacterial class: grey – gamma-proteobacteria; orange – beta-proteobacteria, green – epsilon-proteobacteria; white – Proteobacteria. Branch labels indicate percent bootstrap support.

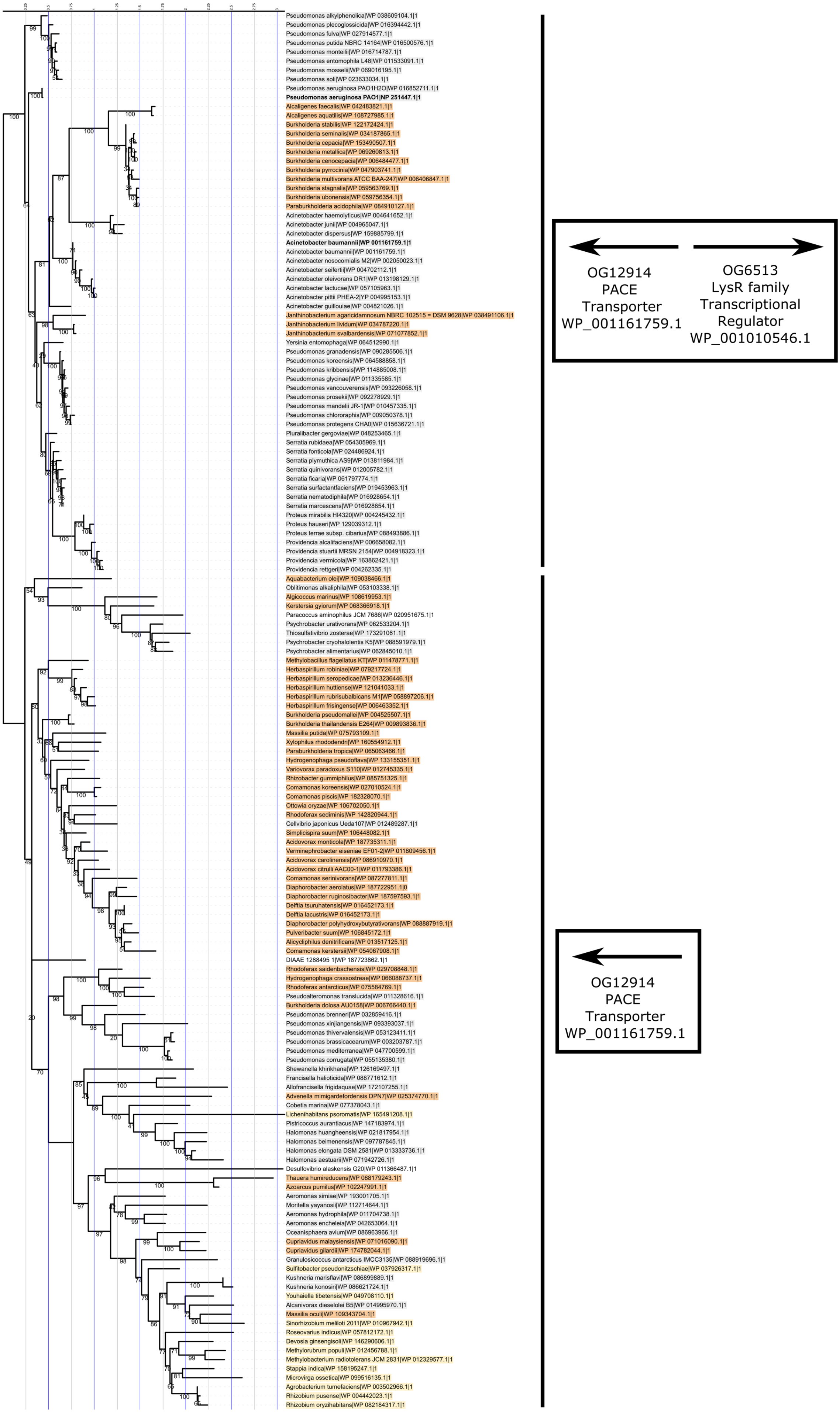
OG12914
PACE
Transporter
WP_001161759.1
OG6513
LysR family
Transcriptional
Regulator
WP_001010546.1
OG12914
PACE
Transporter
WP_001161759.1

Supplementary figure S2. Unrooted maximum likelihood phylogeny of 159 *A. baumannii* AS_1503 orthologs. The two focal proteins, *Acinetobacter baumannii* and *P. aeruginosa* are shown in bold face. Branch labels indicated percent bootstrap support, node labels indicate the species together with the RefSeq protein IDs. The backgrounds of the node lables indicate the bacterial class: grey – gamma-proteobacteria; orange – bate-proteobacteria; yellow – alpha-proteobacteria. Conservation of the gene pair PACE family transporter – LysR family transcriptional regular, that characterises the situation in *A. baumannii* is shown next to the tree.

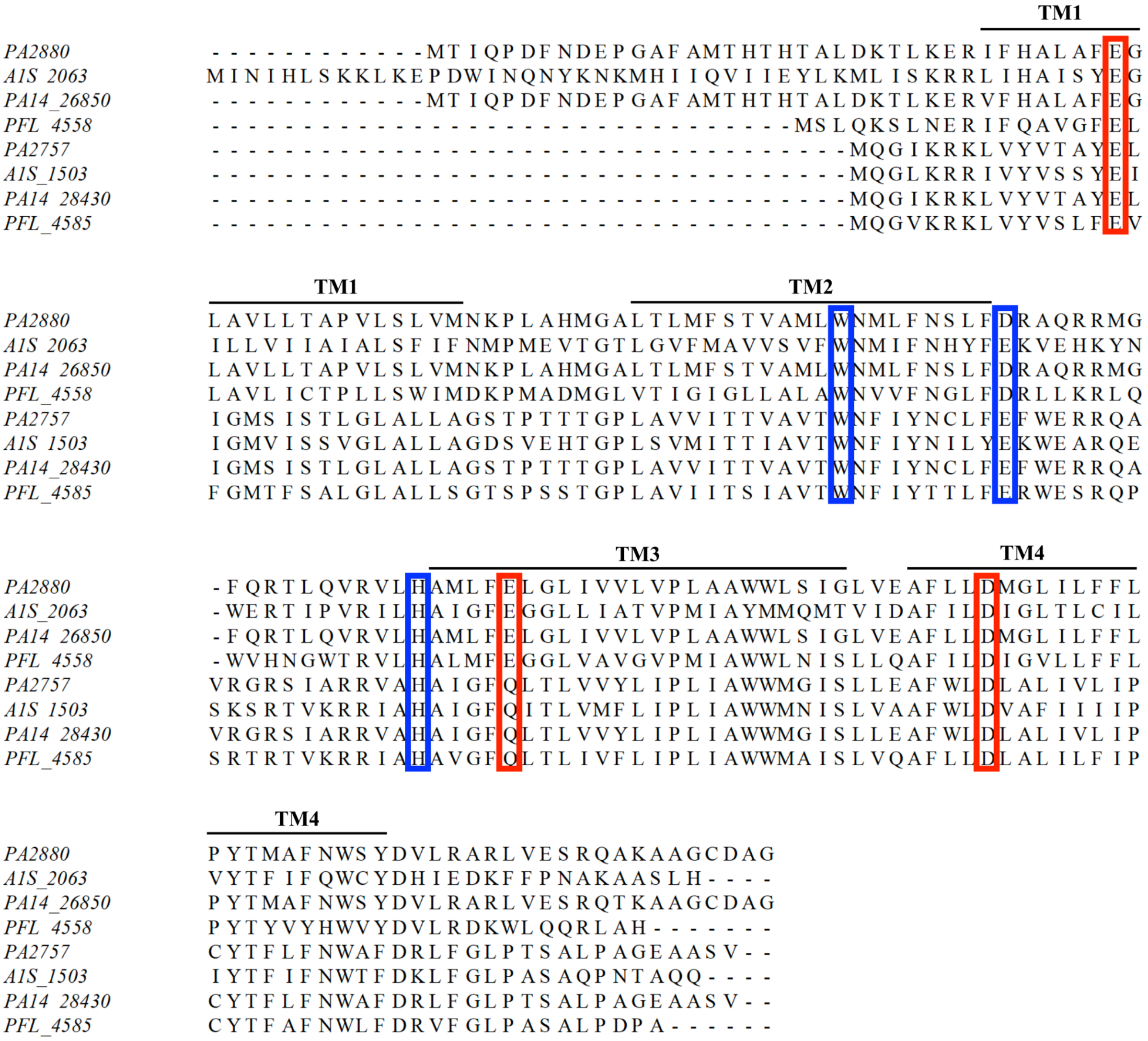

Supplementary figure S3. Sequence alignment of selected PACE family transporters. The locations of the α-helical TMs predicted by TMHMM(1) for PA2880 transporter are shown above the alignment. The residues whose substitution by glutamine/alanine could totally abolish and slightly affect protein transport activity are indicated by red and orange rectangular, respectively.

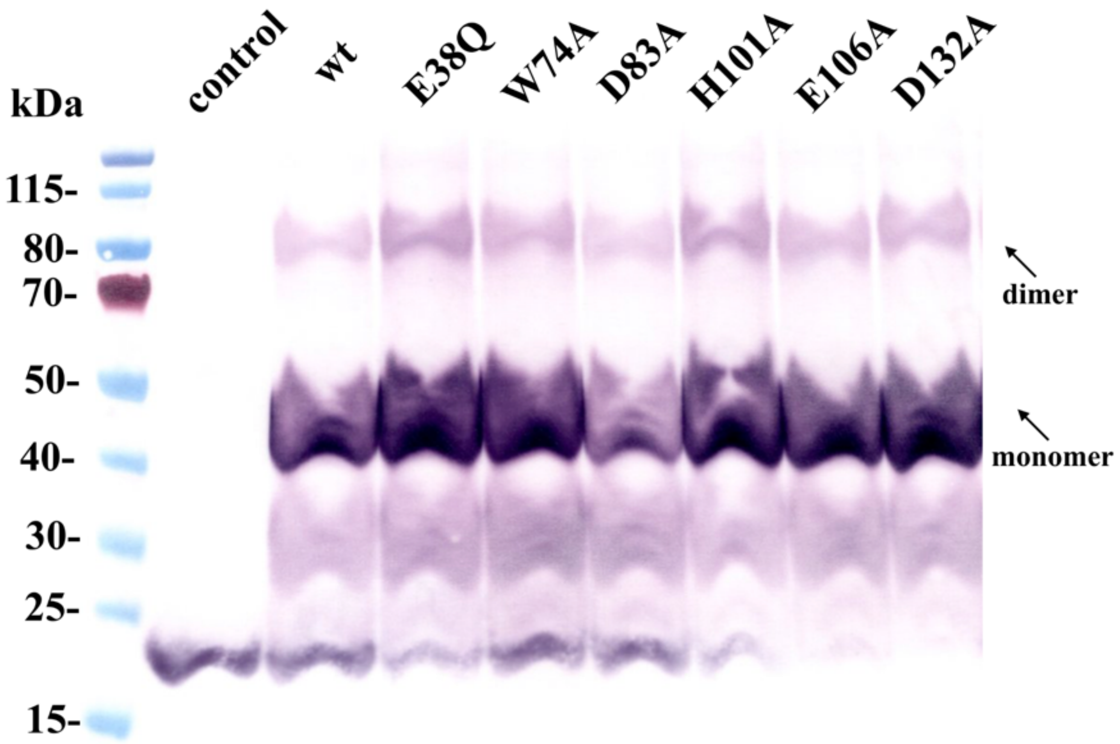

Supplementary figure S4. Western blot of protein PA2880 and variants. The protein was expressed using BL21 cells harboring pTTQ vectors, which include eGFP fusions and a Strep-tag at the C-termini. Monomer and dimer band are indicated by black arrows.

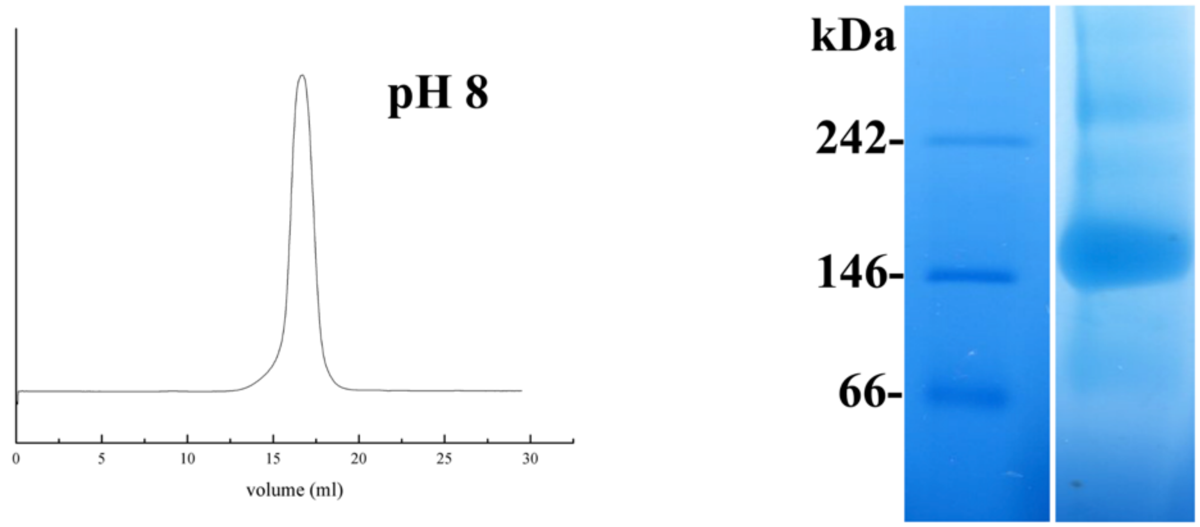

Supplementary figure S5. Size exclusion elution profile of the purified recombinant PA2880. The profile of size exclusion chromatography is shown in left panel. The peak fraction was analyzed by blue native PAGE and the result is shown in the right panel.

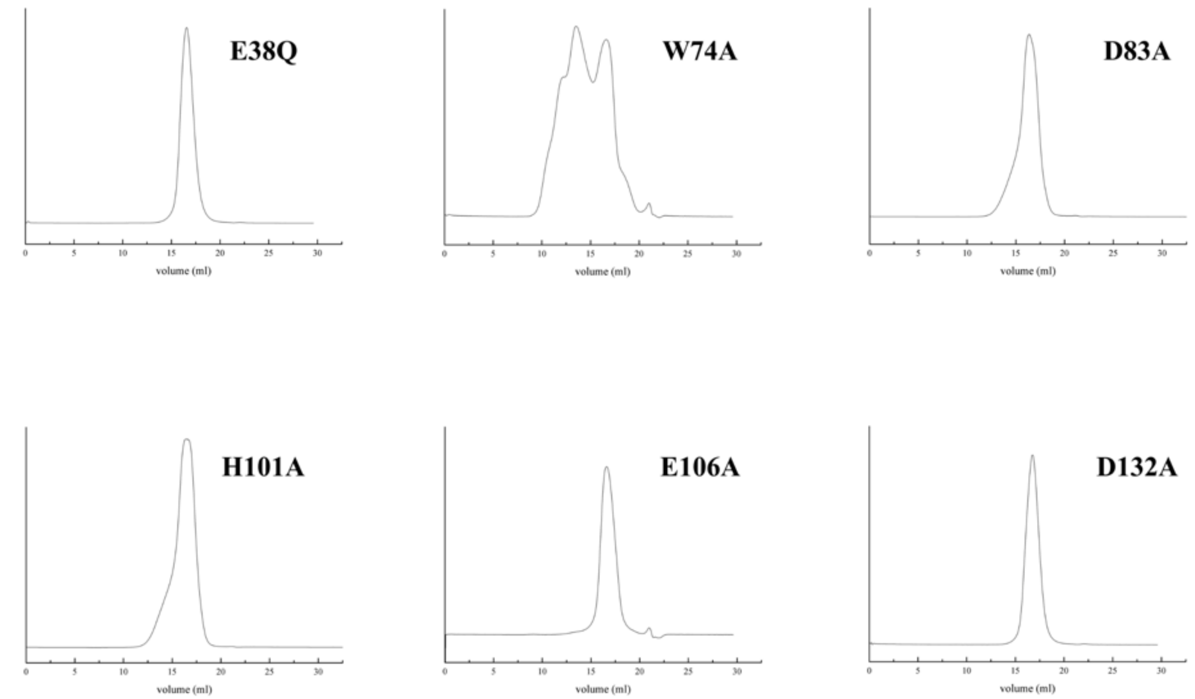

Supplementary figure S6. The elution profiles of the purified PA2880 variants. All proteins were purified at pH 8.

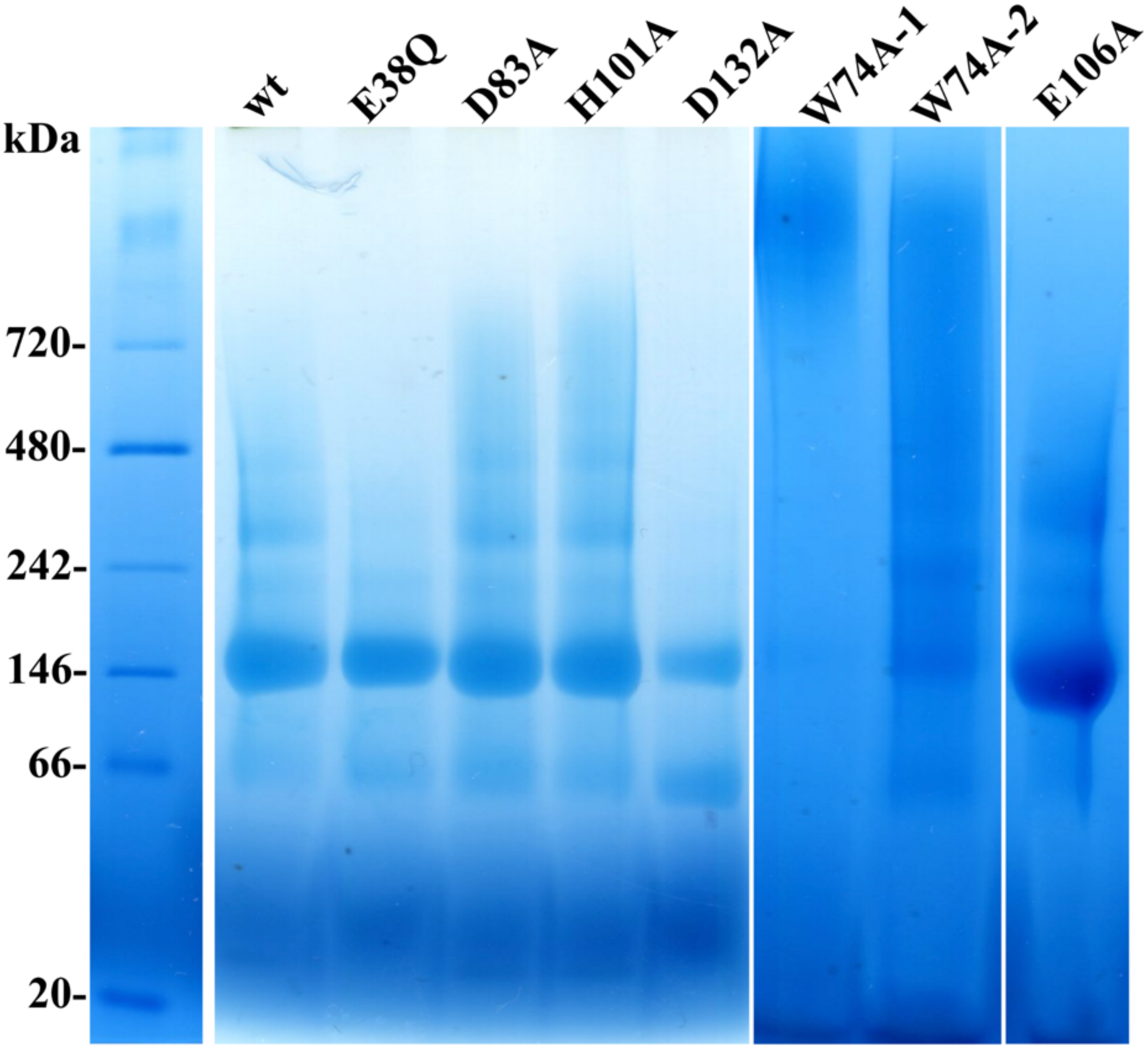

Supplementary figure S7. Blue native PAGE gels of PA2880 and its variants. All samples were purified at pH 8. Clear differences were observed among different samples. For wild type (wt), E38Q, D83A and H101A samples, they migrated as a dominant band with an apparent molecular weight of 146 kDa, and a minor band at 66 kDa, which corresponds to the dimeric and monomeric forms, respectively. The D132A sample had roughly the same amounts of monomers and dimers, while E106A sample mainly showed dimers. W74A-1 and W74A-2 represent the protein eluted at ~12.5 ml and ~16.2 ml, respectively. W74A-1 sample migrated as a high oligomeric complex, and W74A-2 sample behaved similar to D132A.The molecular marker used was the NativeMark unstained protein standard.

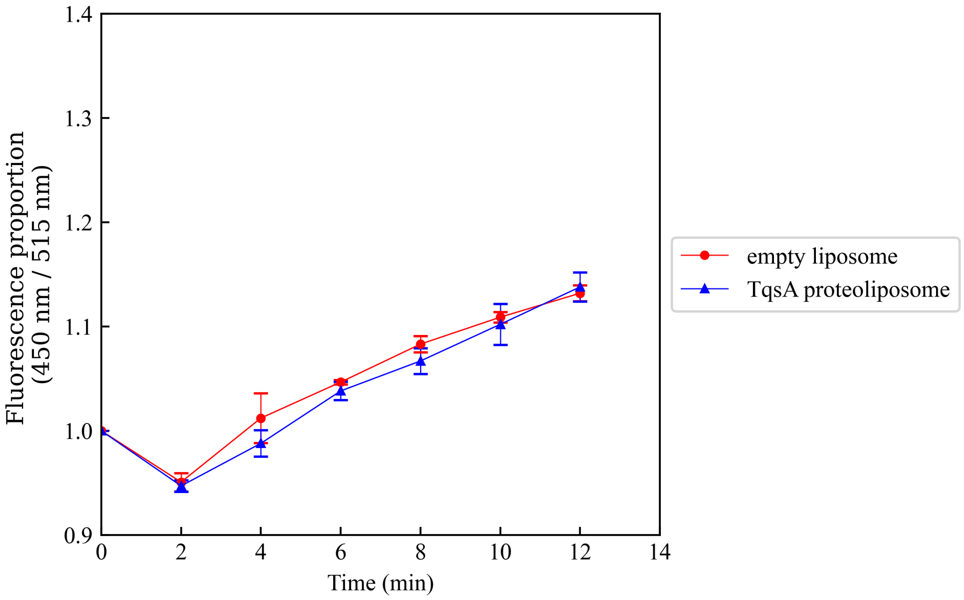

Supplementary figure S8. In vitro transport assay of empty liposomes and proteoliposomes containing TqsA. The experiments were performed under the same condition as that of Figure 2b. The fluorescence intensity measured at time = 0 is normalized to 1. Every experiment was repeated twice, and the error bars were indicated by standard deviations.

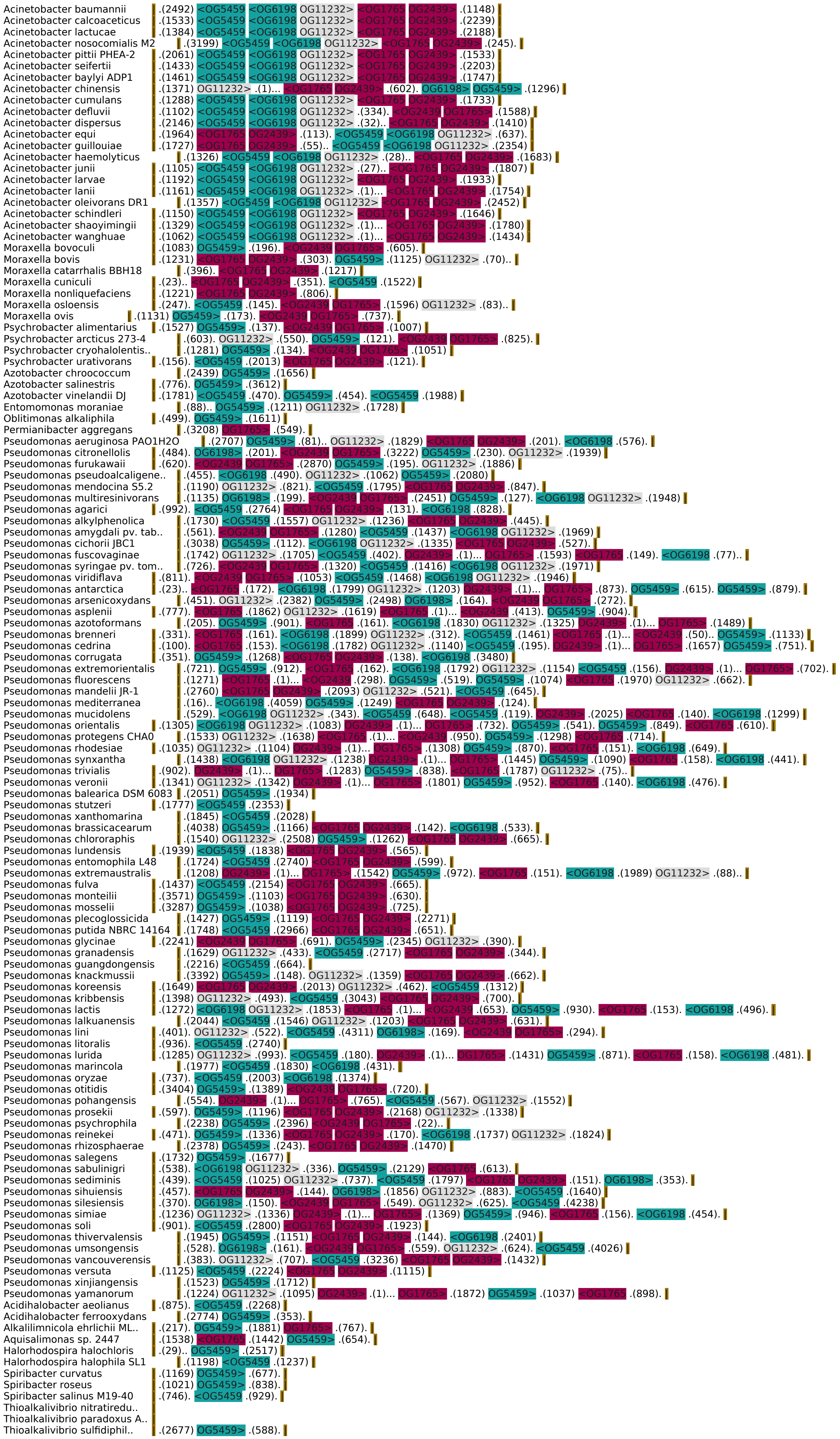
Acinetobacter baumannii .(2492) <OG5459 <OG6198 OG11232> <OG1765 OG2439> .(1148)
Acinetobacter calcoaceticus .(1533) <OG5459 <OG6198 OG11232> <OG1765 OG2439> .(2239)
Acinetobacter lactucae .(1384) <OG5459 <OG6198 OG11232> <OG1765 OG2439> .(2188)
Acinetobacter nosocomialis M2 .(3199) <OG5459 <OG6198 OG11232> <OG1765 OG2439> .(245).
Acinetobacter pittii PHEA-2 .(2061) <OG5459 <OG6198 OG11232> <OG1765 OG2439> .(1533)
Acinetobacter seifertii .(1433) <OG5459 <OG6198 OG11232> <OG1765 OG2439> .(2203)
Acinetobacter baylyi ADP1 .(1461) <OG5459 <OG6198 OG11232> <OG1765 OG2439> .(1747)
Acinetobacter chinensis .(1371) OG11232> .(1)... <OG1765 OG2439> .(602). OG6198> OG5459> .(1296)
Acinetobacter cumulans .(1288) <OG5459 <OG6198 OG11232> <OG1765 OG2439> .(1733)
Acinetobacter defluvii .(1102) <OG5459 <OG6198 OG11232> .(334). <OG2439 OG1765> .(1588)
Acinetobacter dispersus .(2146) <OG5459 <OG6198 OG11232> .(32).. <OG1765 OG2439> .(1410)
Acinetobacter equi .(1964) <OG1765 OG2439> .(113). <OG5459 <OG6198 OG11232> .(637).
Acinetobacter guillouiae .(1727) <OG1765 OG2439> .(55).. <OG5459 <OG6198 OG11232> .(2354)
Acinetobacter haemolyticus .(1326) <OG5459 <OG6198 OG11232> .(28).. <OG1765 OG2439> .(1683)
Acinetobacter junii .(1105) <OG5459 <OG6198 OG11232> .(27).. <OG1765 OG2439> .(1807)
Acinetobacter larvae .(1192) <OG5459 <OG6198 OG11232> <OG1765 OG2439> .(1933)
Acinetobacter lanii .(1161) <OG5459 <OG6198 OG11232> .(1)... <OG1765 OG2439> .(1754)
Acinetobacter oleivorans DR1 .(1357) <OG5459 <OG6198 OG11232> <OG1765 OG2439> .(2452)
Acinetobacter schindleri .(1150) <OG5459 <OG6198 OG11232> <OG1765 OG2439> .(1646)
Acinetobacter shaoyimingii .(1329) <OG5459 <OG6198 OG11232> .(1)... <OG1765 OG2439> .(1780)
Acinetobacter wanghuae .(1062) <OG5459 <OG6198 OG11232> .(1)... <OG1765 OG2439> .(1434)
Moraxella bovoculi .(1083) OG5459> .(196). <OG2439 OG1765> .(605).
Moraxella bovis .(1231) <OG1765 OG2439> .(303). OG5459> .(1125) OG11232> .(70)..
Moraxella catarrhalis BBH18 .(396). <OG1765 OG2439> .(1217)
Moraxella cuniculi .(23).. <OG1765 OG2439> .(351). <OG5459 .(1522)
Moraxella nonliquefaciens .(1221) <OG1765 OG2439> .(806).
Moraxella osloensis .(247). <OG5459 .(145). <OG2439 OG1765> .(1596) OG11232> .(83)..
Moraxella ovis .(1131) OG5459> .(173). <OG2439 OG1765> .(737).
Psychrobacter alimentarius .(1527) OG5459> .(137). <OG2439 OG1765> .(1007)
Psychrobacter arcticus 273-4 .(603). OG11232> .(550). OG5459> .(121). <OG2439 OG1765> .(825).
Psychrobacter cryohalolentis.. .(1281) OG5459> .(134). <OG2439 OG1765> .(1051)
Psychrobacter urativorans .(156). <OG5459 .(2013) <OG1765 OG2439> .(121).
Azotobacter chroococcum .(2439) OG5459> .(1656)
Azotobacter salinestris .(776). OG5459> .(3612)
Azotobacter vinelandii DJ .(1781) <OG5459 .(470). OG5459> .(454). <OG5459 .(1988)
Entomomonas moraniae .(88).. OG5459> .(1211) OG11232> .(1728)
Oblitimonas alkaliphila .(499). OG5459> .(1611)
Permianibacter aggregans .(3208) OG1765> .(549).
Pseudomonas aeruginosa PAO1H2O .(2707) OG5459> .(81).. OG11232> .(1829) <OG1765 OG2439> .(201). <OG6198 .(576).
Pseudomonas citronellolis .(484). OG6198> .(201). <OG2439 OG1765> .(3222) OG5459> .(230). OG11232> .(1939)
Pseudomonas furukawaii .(620). <OG2439 OG1765> .(2870) OG5459> .(195). OG11232> .(1886)
Pseudomonas pseudoalcaligene.. .(455). <OG6198 .(490). OG11232> .(1062) OG5459> .(2080)
Pseudomonas mendocina S5.2 .(1190) OG11232> .(821). <OG5459 .(1795) <OG1765 OG2439> .(847).
Pseudomonas multiresinivorans .(1135) OG6198> .(199). <OG2439 OG1765> .(2451) OG5459> .(127). <OG6198 OG11232> .(1948)
Pseudomonas agarici .(992). <OG5459 .(2764) <OG1765 OG2439> .(131). <OG6198 .(828).
Pseudomonas alkylphenolica .(1730) <OG5459 .(1557) OG11232> .(1236) <OG1765 OG2439> .(445).
Pseudomonas amygdali pv. tab.. .(561). <OG2439 OG1765> .(1280) <OG5459 .(1437) <OG6198 OG11232> .(1969)
Pseudomonas cichorii JBC1 .(3038) OG5459> .(112). <OG6198 OG11232> .(1335) <OG1765 OG2439> .(527).
Pseudomonas fuscovaginae .(1742) OG11232> .(1705) <OG5459 .(402). OG2439> .(1)... OG1765> .(1593) <OG1765 .(149). <OG6198 .(77)..
Pseudomonas syringae pv. tom.. .(726). <OG2439 OG1765> .(1320) <OG5459 .(1416) <OG6198 OG11232> .(1971)
Pseudomonas viridiflava .(811). <OG2439 OG1765> .(1053) <OG5459 .(1468) <OG6198 OG11232> .(1946)
Pseudomonas antarctica .(23).. <OG1765 .(172). <OG6198 .(1799) OG11232> .(1203) OG2439> .(1)... OG1765> .(873). OG5459> .(615). OG5459> .(879).
Pseudomonas arsenicoxydans .(451). OG11232> .(2382) OG5459> .(2498) OG6198> .(164). <OG2439 OG1765> .(272).
Pseudomonas asplenii .(777). <OG1765 .(1862) OG11232> .(1619) <OG1765 .(1)... <OG2439 .(413). OG5459> .(904).
Pseudomonas azotoformans .(205). OG5459> .(901). <OG1765 .(161). <OG6198 .(1830) OG11232> .(1325) OG2439> .(1)... OG1765> .(1489)
Pseudomonas brenneri .(331). <OG1765 .(161). <OG6198 .(1461) <OG1765 .(1)... <OG2439 .(50).. OG5459> .(1133)
Pseudomonas cedrina .(100). <OG1765 .(153). <OG6198 .(1782) OG11232> .(1140) <OG5459 .(195). OG2439> .(1)... OG1765> .(1657) OG5459> .(751).
Pseudomonas corrugata .(351). OG5459> .(1268) <OG1765 OG2439> .(138). <OG6198 .(3480)
Pseudomonas extremorientalis .(721). OG5459> .(912). <OG1765 .(162). <OG6198 .(1792) OG11232> .(1154) <OG5459 .(156). OG2439> .(1)... OG1765> .(702).
Pseudomonas fluorescens .(1271) <OG1765 .(1)... <OG2439 .(298). OG5459> .(519). OG5459> .(1074) <OG1765 .(1970) OG11232> .(662).
Pseudomonas mandelii JR-1 .(2760) <OG1765 OG2439> .(2093) OG11232> .(521). <OG5459 .(645).
Pseudomonas mediterranea .(16).. <OG6198 .(4059) OG5459> .(1249) <OG1765 OG2439> .(124).
Pseudomonas mucidolens .(529). <OG6198 OG11232> .(343). <OG5459 .(648). <OG5459 .(119). OG2439> .(2025) <OG1765 .(140). <OG6198 .(1299)
Pseudomonas orientalis .(1305) <OG6198 OG11232> .(1083) OG2439> .(1)... OG1765> .(732). OG5459> .(541). OG5459> .(849). <OG1765 .(610).
Pseudomonas protegens CHA0 .(1533) OG11232> .(1638) <OG1765 .(1)... <OG2439 .(950). OG5459> .(1298) <OG1765 .(714).
Pseudomonas rhodesiae .(1035) OG11232> .(1104) OG2439> .(1)... OG1765> .(1308) OG5459> .(870). <OG1765 .(151). <OG6198 .(649).
Pseudomonas synxantha .(1438) <OG6198 OG11232> .(1238) OG2439> .(1)... OG1765> .(1445) OG5459> .(1090) <OG1765 .(158). <OG6198 .(441).
Pseudomonas trivialis .(902). OG2439> .(1)... OG1765> .(1283) OG5459> .(838). <OG1765 .(1787) OG11232> .(75)..
Pseudomonas veronii .(1341) OG11232> .(1342) OG2439> .(1)... OG1765> .(1801) OG5459> .(952). <OG1765 .(140). <OG6198 .(476).
Pseudomonas balearica DSM 6083 .(2051) OG5459> .(1934)
Pseudomonas stutzeri .(1777) <OG5459 .(2353)
Pseudomonas xanthomarina .(1845) <OG5459 .(2028)
Pseudomonas brassicacearum .(4038) OG5459> .(1166) <OG1765 OG2439> .(142). <OG6198 .(533).
Pseudomonas chlororaphis .(1540) OG11232> .(2508) OG5459> .(1262) <OG1765 OG2439> .(665).
Pseudomonas lundensis .(1939) <OG5459 .(1838) <OG1765 OG2439> .(565).
Pseudomonas entomophila L48 .(1724) <OG5459 .(2740) <OG1765 OG2439> .(599).
Pseudomonas extremaustralis .(1208) OG2439> .(1)... OG1765> .(1542) OG5459> .(972). <OG1765 .(151). <OG6198 .(1989) OG11232> .(88)..
Pseudomonas fulva .(1437) <OG5459 .(2154) <OG1765 OG2439> .(665).
Pseudomonas monteilii .(3571) OG5459> .(1103) <OG1765 OG2439> .(630).
Pseudomonas mosselii .(3287) OG5459> .(1038) <OG1765 OG2439> .(725).
Pseudomonas plecoglossicida .(1427) OG5459> .(1119) <OG1765 OG2439> .(2271)
Pseudomonas putida NBRC 14164 .(1748) <OG5459 .(2966) <OG1765 OG2439> .(651).
Pseudomonas glycinae .(2241) <OG2439 OG1765> .(691). OG5459> .(2345) OG11232> .(390).
Pseudomonas granadensis .(1629) OG11232> .(433). <OG5459 .(2717) <OG1765 OG2439> .(344).
Pseudomonas guangdongensis .(2216) <OG5459 .(664).
Pseudomonas knackmussii .(3392) OG5459> .(148). OG11232> .(1359) <OG1765 OG2439> .(662).
Pseudomonas koreensis .(1649) <OG1765 OG2439> .(2013) OG11232> .(462). <OG5459 .(1312)
Pseudomonas kribbensis .(1398) OG11232> .(493). <OG5459 .(3043) <OG1765 OG2439> .(700).
Pseudomonas lactis .(1272) <OG6198 OG11232> .(1853) <OG1765 .(1)... <OG2439 .(653). OG5459> .(930). <OG1765 .(153). <OG6198 .(496).
Pseudomonas lalkuanensis .(2044) <OG5459 .(1546) OG11232> .(1203) <OG1765 OG2439> .(631).
Pseudomonas lini .(401). OG11232> .(522). <OG5459 .(4311) OG6198> .(169). <OG2439 OG1765> .(294).
Pseudomonas litoralis .(936). <OG5459 .(2740)
Pseudomonas lurida .(1285) OG11232> .(993). <OG5459 .(180). OG2439> .(1)... OG1765> .(1431) OG5459> .(871). <OG1765 .(158). <OG6198 .(481).
Pseudomonas marincola .(1977) <OG5459 .(1830) <OG6198 .(431).
Pseudomonas oryzae .(737). <OG5459 .(2003) <OG6198 .(1374)
Pseudomonas otitidis .(3404) OG5459> .(1389) <OG2439 OG1765> .(720).
Pseudomonas pohangensis .(554). OG2439> .(1)... OG1765> .(765). <OG5459 .(567). OG11232> .(1552)
Pseudomonas prosekii .(597). OG5459> .(1196) <OG1765 OG2439> .(2168) OG11232> .(1338)
Pseudomonas psychrophila .(2238) OG5459> .(2396) <OG2439 OG1765> .(22)..
Pseudomonas reinekei .(471). OG5459> .(1336) <OG1765 OG2439> .(170). <OG6198 .(1737) OG11232> .(1824)
Pseudomonas rhizosphaerae .(2378) OG5459> .(243). <OG1765 OG2439> .(1470)
Pseudomonas salegens .(1732) OG5459> .(1677)
Pseudomonas sabulinigri .(538). <OG6198 OG11232> .(336). OG5459> .(2129) <OG1765 .(613).
Pseudomonas sediminis .(439). <OG5459 .(1025) OG11232> .(737). <OG5459 .(1797) <OG1765 OG2439> .(151). OG6198> .(353).
Pseudomonas sihuiensis .(457). <OG1765 OG2439> .(144). OG6198> .(1856) OG11232> .(883). <OG5459 .(1640)
Pseudomonas silesiensis .(370). OG6198> .(150). <OG2439 OG1765> .(549). OG11232> .(625). <OG5459 .(4238)
Pseudomonas simiae .(1236) OG11232> .(1336) OG2439> .(1)... OG1765> .(1369) OG5459> .(946). <OG1765 .(156). <OG6198 .(454).
Pseudomonas soli .(901). <OG5459 .(2800) <OG1765 OG2439> .(1923)
Pseudomonas thivervalensis .(1945) OG5459> .(1151) <OG1765 OG2439> .(144). <OG6198 .(2401)
Pseudomonas umsongensis .(528). OG6198> .(161). <OG2439 OG1765> .(559). OG11232> .(624). <OG5459 .(4026)
Pseudomonas vancouverensis .(383). OG11232> .(707). <OG5459 .(3236) <OG1765 OG2439> .(1432)
Pseudomonas versuta .(1125) <OG5459 .(2224) <OG1765 OG2439> .(1115)
Pseudomonas xinjiangensis .(1523) OG5459> .(1712)
Pseudomonas yamanorum .(1224) OG11232> .(1095) OG2439> .(1)... OG1765> .(1872) OG5459> .(1037) <OG1765 .(898).
Acidihalobacter aeolianus .(875). <OG5459 .(2268)
Acidihalobacter ferrooxydans .(2774) OG5459> .(353).
Alkalilimnicola ehrlichii ML.. .(217). OG5459> .(1881) OG1765> .(767).
Aquisalimonas sp. 2447 .(1538) <OG1765 .(1442) OG5459> .(654).
Halorhodospira halochloris .(29).. OG5459> .(2517)
Halorhodospira halophila SL1 .(1198) <OG5459 .(1237)
Spiribacter curvatus .(1169) OG5459> .(677).
Spiribacter roseus .(1021) OG5459> .(838).
Spiribacter salinus M19-40 .(746). <OG5459 .(929).
Thioalkalivibrio nitratiredu..
Thioalkalivibrio paradoxus A..
Thioalkalivibrio sulfidiphil.. .(2677) OG5459> .(588).

Thioalkalivibrio versutus
Allochromatium vinosum DSM 180 .(2250) <OG5459 .(799).
Marichromatium purpuratum 984 .(2372) OG5459> .(859).
Nitrosococcus halophilus Nc 4 .(1915) OG1765> .(730). <OG5459 .(1036)
Nitrosococcus oceani ATCC 19.. .(267). <OG5459 .(1474) <OG1765 .(1351)
Nitrosococcus watsonii C-113 .(1303) OG1765> .(1587)
Nitrosococcus wardiae .(2822) <OG1765 .(848).
Thermochromatium tepidum ATC..
Thioflavicoccus mobilis 8321 .(1538) OG5459> .(1930)
Thiocystis violascens DSM 198 .(2767) <OG5459 .(1598)
Granulosicoccus antarcticus .. .(3769) <OG5459 .(1514) <OG1765 .(1354)
Sulfuriflexus mobilis .(1530) OG5459> .(11).. OG6198> .(26).. OG1765> .(1486)
Guyparkeria halophila .(2229) <OG5459 .(62)..
Halothiobacillus neapolitanu.. .(157). OG5459> .(820). OG6198> .(1342)
Sulfurivermis fontis .(417). OG5459> .(2767)
Wenzhouxiangella marina .(1551) <OG1765 .(1506)
Woeseia oceani .(121). <OG1765 .(3423)
Actinobacillus delphinicola .(720). <OG5459 .(897).
Actinobacillus equuli subsp... .(257). OG11232> .(557). <OG5459 .(303). <OG1765 .(1061)
Actinobacillus pleuropneumon.. .(575). OG11232> .(610). <OG1765 .(829). OG5459> .(59)..
Actinobacillus porcitonsilla.. .(986). <OG5459 .(517). OG11232> .(345). OG1765> .(187).
Actinobacillus suis ATCC 33415 .(1041) OG1765> .(318). OG5459> .(369). OG11232> .(504).
Aggregatibacter actinomycete.. .(1768) <OG5459 .(129).
Aggregatibacter aphrophilus .. .(1263) <OG5459 .(795).
Aggregatibacter segnis ATCC .. .(333). <OG5459 .(1511)
Avibacterium volantium .(125). OG5459> .(1880)
Basfia succiniciproducens .(1640) OG5459> .(363).
Bibersteinia trehalosi USDA-.. .(1189) <OG1765 .(543). OG5459> .(146). OG11232> .(166).
Bisgaardia hudsonensis .(248). <OG5459 .(1614)
Frederiksenia canicola .(112). OG5459> .(952). OG1765> .(885).
Glaesserella parasuis SH0165 .(625). <OG1765 .(326). <OG5459 .(1095)
Haemophilus aegyptius .(1126) OG5459> .(724).
Haemophilus haemolyticus .(253). OG5459> .(1498)
Haemophilus influenzae .(1613) <OG5459 .(99)..
Haemophilus pittmaniae .(699). OG5459> .(1262)
Histophilus somni .(210). <OG5459 .(1683)
Mannheimia haemolytica USMAR.. .(261). OG11232> .(2070) OG5459> .(196).
Mannheimia ovis .(751). <OG5459 .(1214)
Mannheimia varigena USDA-ARS.. .(397). <OG5459 .(1522)
Otariodibacter oris .(620). OG1765> .(915). OG5459> .(288).
Pasteurella dagmatis .(1574) <OG5459 .(427).
Pasteurella multocida .(538). <OG5459 .(280). <OG5459 .(1229)
Pasteurella skyensis .(1668) <OG5459 .(400).
Rodentibacter heylii .(1472) <OG5459 .(176). OG11232> .(724).
Vespertiliibacter pulmonis .(218). <OG1765 .(252). OG5459> .(1190)
Aeromonas allosaccharophila .(29).. OG5459> .(137). <OG6198 OG11232> .(2807) OG1765> .(1050)
Aeromonas encheleia .(618). OG6198> .(1490) <OG1765 .(822). OG5459> .(1075)
Aeromonas hydrophila .(785). OG6198> .(1556) <OG1765 .(909). OG5459> .(1108)
Aeromonas media .(1102) <OG5459 .(846). OG1765> .(1483) <OG6198 OG11232> .(404). OG5459> .(295).
Aeromonas salmonicida .(280). <OG6198 OG11232> .(2755) <OG1765 .(853). OG5459> .(149).
Aeromonas simiae .(961). OG6198> .(53).. <OG5459 .(1174) <OG1765 .(1362)
Aeromonas veronii .(866). <OG1765 .(1099) <OG6198 OG11232> .(1806) <OG5459 .(231).
Oceanisphaera avium .(1360) <OG5459 .(707). <OG1765 .(74).. OG6198> .(371).
Oceanisphaera profunda .(595). OG5459> .(414). OG11232> .(1649) OG1765> .(232).
Tolumonas auensis DSM 9187 .(15).. OG5459> .(69).. <OG5459 .(15).. <OG5459 .(900). <OG5459 .(320). <OG5459 .(1815)
Zobellella denitrificans .(1861) OG1765> .(799). OG5459> .(688). <OG6198 OG11232> .(458).
Aerosticca soli .(48).. OG1765> .(120). OG5459> .(2428)
Ahniella affigens .(3825) <OG1765 .(632).
Dokdonella koreensis DS-123 .(129). <OG1765 .(3435)
Dyella thiooxydans .(2456) OG5459> .(524). OG1765> .(788).
Frateuria aurantia DSM 6220 .(1004) <OG1765 .(1)... <OG2439 .(1355) OG5459> .(742).
Luteibacter pinisoli .(211). OG1765> .(3475) OG5459> .(353). <OG6198 .(57)..
Luteibacter rhizovicinus DSM.. .(1027) OG5459> .(626). OG1765> .(2616)
Rhodanobacter denitrificans .(3744) <OG1765 .(91)..
Arenimonas daejeonensis
Luteimonas chenhongjianii .(614). OG1765> .(18).. OG5459> .(2278)
Luteimonas granuli .(1272) <OG1765 .(1218)
Lysobacter alkalisoli .(2962) OG5459> .(107). <OG1765 .(233).
Lysobacter antibioticus .(311). <OG5459 .(4033) <OG1765 .(455).
Lysobacter capsici .(612). <OG5459 .(2239) OG6198> .(1984) <OG1765 .(260).
Lysobacter enzymogenes .(398). OG1765> .(4062) OG5459> .(373).
Lysobacter gummosus .(386). <OG5459 .(366). <OG6198 .(3899) <OG1765 .(296).
Lysobacter lycopersici .(1718) <OG1765 .(779).
Lysobacter maris .(211). OG1765> .(218). <OG5459 .(2970)
Lysobacter oculi .(1882) <OG1765 .(515).
Lysobacter soli .(271). <OG5459 .(3298) OG1765> .(87)..
Pseudolysobacter antarcticus .(234). OG1765> .(3712)
Pseudoxanthomonas mexicana .(1853) <OG6198 .(202). OG5459> .(795). <OG1765 .(793).
Pseudoxanthomonas spadix BD-.. .(212). OG1765> .(2154) OG5459> .(719).
Stenotrophomonas acidaminiph.. .(815). <OG5459 .(560). OG5459> .(1847) <OG1765 .(383).
Stenotrophomonas maltophilia .(1778) <OG6198 .(1746) OG5459> .(577). <OG1765 .(135).
Stenotrophomonas rhizophila .(207). OG1765> .(608). <OG5459 .(1322) OG6198> .(1653) OG11232> .(196).
Thermomonas brevis .(1496) OG1765> .(283). OG5459> .(1302)
Xanthomonas albilineans .(368). <OG5459 .(2494) <OG1765 .(85)..
Xanthomonas cassavae CFBP 4642 .(2467) <OG6198 .(472). OG5459> .(830). <OG1765 .(400).
Xanthomonas campestris pv. r.. .(913). <OG5459 .(822). OG6198> .(2091) <OG1765 .(212).
Xanthomonas citri .(2425) <OG6198 .(813). OG5459> .(689). <OG1765 .(184).
Xanthomonas cucurbitae .(665). <OG5459 .(2872) <OG1765 .(151).
Xanthomonas euroxanthea .(2247) <OG6198 .(878). OG5459> .(651). <OG1765 .(208).
Xanthomonas fragariae .(328). OG1765> .(472). <OG5459 .(548). OG6198> .(1868)
Xanthomonas hortorum .(2052) OG5459> .(1109) OG1765> .(989).
Xanthomonas hyacinthi .(601). <OG6198 .(1732) OG1765> .(763). <OG5459 .(909).
Xanthomonas oryzae pv. oryzi.. .(2031) <OG6198 .(822). OG5459> .(459). <OG1765 .(157).
Xanthomonas phaseoli pv. die.. .(977). OG6198> .(1434) OG5459> .(659). <OG1765 .(907).
Xanthomonas vesicatoria ATCC.. .(33).. <OG6198 .(878). OG5459> .(698). <OG1765 .(2494)
Xylella fastidiosa Temecula1
Xylella taiwanensis
Agarilytica rhodophyticola .(3336) <OG6198 .(558). OG5459> .(1615)
Cellvibrio japonicus Ueda107 .(1409) <OG5459 .(2103) OG6198> .(102).
Saccharophagus degradans 2-40 .(787). OG5459> .(713). <OG5459 .(2553)
Simiduia agarivorans SA1 = D.. .(1480) OG6198> .(2144) OG5459> .(117).
Congregibacter litoralis KT71 .(2355) OG5459> .(1450)
Halioglobus maricola .(2568) OG5459> .(1297)
Kineobactrum salinum .(143). OG5459> .(3769)
Microbulbifer aggregans .(103). OG5459> .(2639) OG6198> .(447). OG1765> OG2439> .(95)..
Microbulbifer agarilyticus .(2275) OG6198> .(366). OG5459> .(658). OG5459> .(155).
Microbulbifer hydrolyticus .(300). <OG5459 .(442). <OG5459 .(1310) OG6198> .(1389)
Microbulbifer thermotolerans .(2353) OG5459> .(805). <OG6198 .(60)..
Oceanicoccus sagamiensis .(2888) <OG5459 .(1033)
Zhongshania aliphaticivorans .(14).. OG6198> .(1474) <OG5459 .(1081) <OG1765 OG2439> .(1107)
Alcanivorax borkumensis SK2 .(1517) OG5459> .(197). <OG1765 OG2439> .(1033)
Alcanivorax dieselolei B5 .(797). <OG6198 .(1156) <OG5459 .(996). <OG1765 OG2439> .(1434)
Alcanivorax pacificus W11-5 .(864). <OG6198 .(710). <OG5459 .(1033) <OG1765 OG2439> .(1090)
Ketobacter alkanivorans .(86).. OG5459> .(413). <OG1765 OG2439> .(3639)
Bermanella marisrubri .(980). <OG2439 .(610). OG5459> .(353). <OG1765 .(1234)
Marinobacterium aestuarii .(825). OG11232> .(743). OG5459> .(1004) <OG6198 .(449). OG5459> .(1436)
Marinomonas arctica .(2205) OG5459> .(1752) <OG6198 .(100).
Marinomonas mediterranea MMB-1 .(1750) <OG5459 .(366). OG6198> .(1986)
Marinomonas posidonica IVIA-.. .(1502) <OG5459 .(1890) <OG6198 .(83)..
Marinomonas primoryensis .(1631) <OG5459 .(1905) <OG6198 .(90)..
Neptunomonas concharum .(1006) <OG5459 .(2272)
Neptunomonas phycophila .(1000) OG5459> .(2545)
Thalassolituus oleivorans MI.. .(1813) <OG5459 .(532). <OG1765 OG2439> .(1166)
Chromohalobacter salexigens .. .(351). <OG5459 .(290). OG5459> .(2658)
Cobetia marina .(388). <OG6198 .(2412) OG5459> .(558).
Halomonas aestuarii .(1197) OG6198> .(1436) <OG5459 .(582).

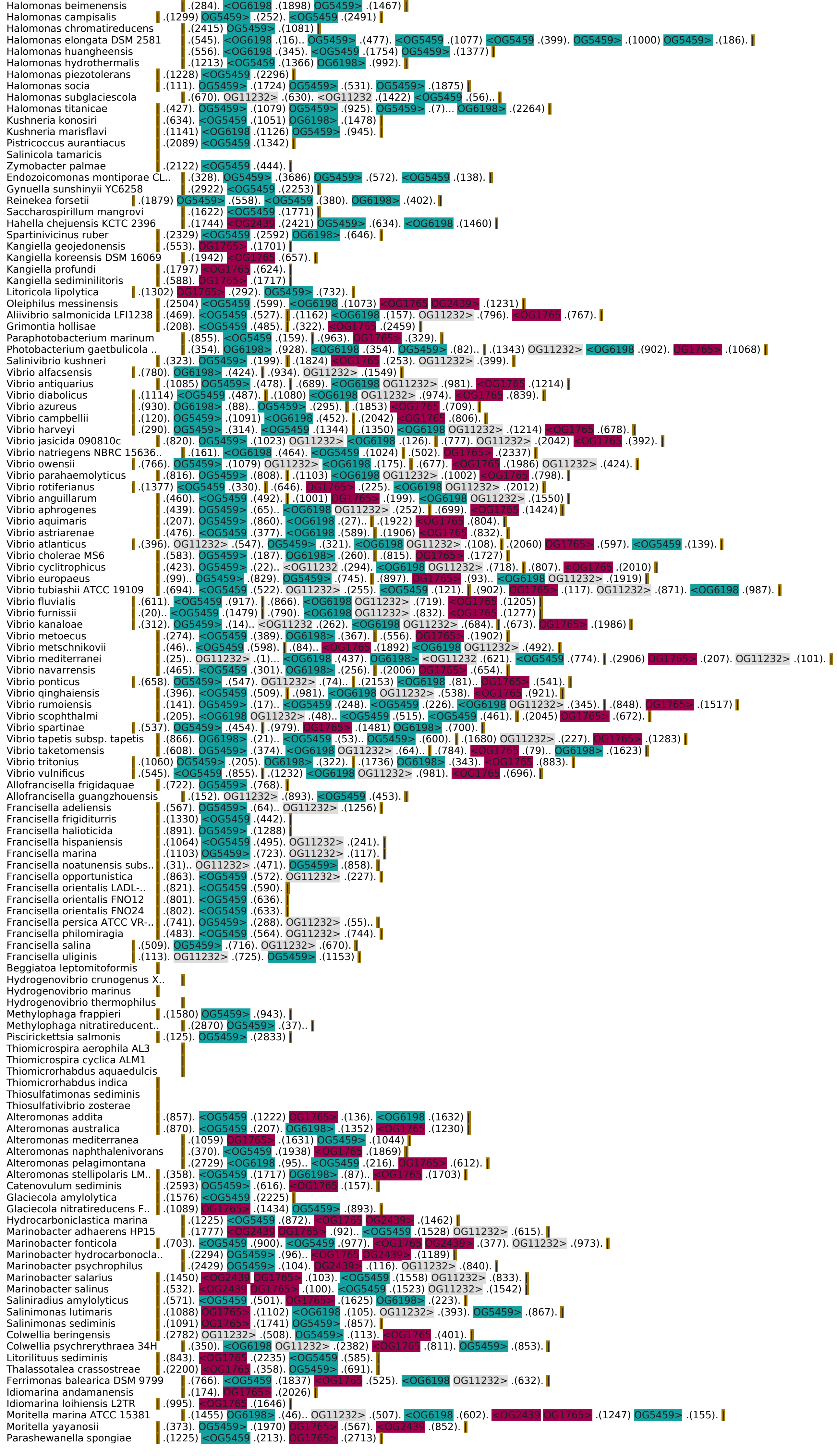
Halomonas beimenensis .(284). <OG6198 .(1898) OG5459> .(1467)
Halomonas campisalis .(1299) OG5459> .(252). <OG5459 .(2491)
Halomonas chromatireducens .(2415) OG5459> .(1081)
Halomonas elongata DSM 2581 .(545). <OG6198 .(16).. OG5459> .(477). <OG5459 .(1077) <OG5459 .(399). OG5459> .(1000) OG5459> .(186).
Halomonas huangheensis .(556). <OG6198 .(345). <OG5459 .(1754) OG5459> .(1377)
Halomonas hydrothermalis .(1213) <OG5459 .(1366) OG6198> .(992).
Halomonas piezotolerans .(1228) <OG5459 .(2296)
Halomonas socia .(111). OG5459> .(1724) OG5459> .(531). OG5459> .(1875)
Halomonas subglaciescola .(670). OG11232> .(630). <OG11232 .(1422) <OG5459 .(56)..
Halomonas titanicae .(427). OG5459> .(1079) OG5459> .(925). OG5459> .(7)... OG6198> .(2264)
Kushneria konosiri .(634). <OG5459 .(1051) OG6198> .(1478)
Kushneria marisflavi .(1141) <OG6198 .(1126) OG5459> .(945).
Pistricoccus aurantiacus .(2089) <OG5459 .(1342)
Salinicola tamaricis
Zymobacter palmae .(2122) <OG5459 .(444).
Endozoicomonas montiporae CL.. .(328). OG5459> .(3686) OG5459> .(572). <OG5459 .(138).
Gynuella sunshinyii YC6258 .(2922) <OG5459 .(2253)
Reinekea forsetii .(1879) OG5459> .(558). <OG5459 .(380). OG6198> .(402).
Saccharospirillum mangrovi .(1622) <OG5459 .(1771)
Hahella chejuensis KCTC 2396 .(1744) <OG2439 .(2421) OG5459> .(634). <OG6198 .(1460)
Spartinivicinus ruber .(2329) <OG5459 .(2592) OG6198> .(646).
Kangiella geojedonensis .(553). OG1765> .(1701)
Kangiella koreensis DSM 16069 .(1942) <OG1765 .(657).
Kangiella profundi .(1797) <OG1765 .(624).
Kangiella sediminilitoris .(588). OG1765> .(1717)
Litoricola lipolytica .(1302) OG1765> .(292). OG5459> .(732).
Oleiphilus messinensis .(2504) <OG5459 .(599). <OG6198 .(1073) <OG1765 OG2439> .(1231)
Aliivibrio salmonicida LFI1238 .(469). <OG5459 .(527). .(1162) <OG6198 .(157). OG11232> .(796). <OG1765 .(767).
Grimontia hollisae .(208). <OG5459 .(485). .(322). <OG1765 .(2459)
Paraphotobacterium marinum .(855). <OG5459 .(159). .(963). OG1765> .(329).
Photobacterium gaetbulicola .. .(354). OG6198> .(928). <OG6198 .(354). OG5459> .(82).. .(1343) OG11232> <OG6198 .(902). OG1765> .(1068)
Salinivibrio kushneri .(323). OG5459> .(199). .(1824) <OG1765 .(253). OG11232> .(399).
Vibrio alfacsensis .(780). OG6198> .(424). .(934). OG11232> .(1549)
Vibrio antiquarius .(1085) OG5459> .(478). .(689). <OG6198 OG11232> .(981). <OG1765 .(1214)
Vibrio diabolicus .(1114) <OG5459 .(487). .(1080) <OG6198 OG11232> .(974). <OG1765 .(839).
Vibrio azureus .(930). OG6198> .(88).. OG5459> .(295). .(1853) <OG1765 .(709).
Vibrio campbellii .(120). OG5459> .(1091) <OG6198 .(452). .(2042) <OG1765 .(806).
Vibrio harveyi .(290). OG5459> .(314). <OG5459 .(1344) .(1350) <OG6198 OG11232> .(1214) <OG1765 .(678).
Vibrio jasicida 090810c .(820). OG5459> .(1023) OG11232> <OG6198 .(126). .(777). OG11232> .(2042) <OG1765 .(392).
Vibrio natriegens NBRC 15636.. .(161). <OG6198 .(464). <OG5459 .(1024) .(502). OG1765> .(2337)
Vibrio owensii .(766). OG5459> .(1079) OG11232> <OG6198 .(175). .(677). <OG1765 .(1986) OG11232> .(424).
Vibrio parahaemolyticus .(816). OG5459> .(808). .(1103) <OG6198 OG11232> .(1002) <OG1765 .(798).
Vibrio rotiferianus .(1377) <OG5459 .(330). .(646). OG1765> .(225). <OG6198 OG11232> .(2012)
Vibrio anguillarum .(460). <OG5459 .(492). .(1001) OG1765> .(199). <OG6198 OG11232> .(1550)
Vibrio aphrogenes .(439). OG5459> .(65).. <OG6198 OG11232> .(252). .(699). <OG1765 .(1424)
Vibrio aquimaris .(207). OG5459> .(860). <OG6198 .(27).. .(1922) <OG1765 .(804).
Vibrio astriarenae .(476). <OG5459 .(377). <OG6198 .(589). .(1906) <OG1765 .(832).
Vibrio atlanticus .(396). OG11232> .(547). OG5459> .(321). <OG6198 OG11232> .(108). .(2060) OG1765> .(597). <OG5459 .(139).
Vibrio cholerae MS6 .(583). OG5459> .(187). OG6198> .(260). .(815). OG1765> .(1727)
Vibrio cyclitrophicus .(423). OG5459> .(22).. <OG11232 .(294). <OG6198 OG11232> .(718). .(807). <OG1765 .(2010)
Vibrio europaeus .(99).. OG5459> .(829). OG5459> .(745). .(897). OG1765> .(93).. <OG6198 OG11232> .(1919)
Vibrio tubiashii ATCC 19109 .(694). <OG5459 .(522). OG11232> .(255). <OG5459 .(121). .(902). OG1765> .(117). OG11232> .(871). <OG6198 .(987).
Vibrio fluvialis .(611). <OG5459 .(917). .(866). <OG6198 OG11232> .(719). <OG1765 .(1205)
Vibrio furnissii .(20).. <OG5459 .(1479) .(790). <OG6198 OG11232> .(832). <OG1765 .(1277)
Vibrio kanaloae .(312). OG5459> .(14).. <OG11232 .(262). <OG6198 OG11232> .(684). .(673). OG1765> .(1986)
Vibrio metoecus .(274). <OG5459 .(389). OG6198> .(367). .(556). OG1765> .(1902)
Vibrio metschnikovii .(46).. <OG5459 .(598). .(84).. <OG1765 .(1892) <OG6198 OG11232> .(492).
Vibrio mediterranei .(25).. OG11232> .(1)... <OG6198 .(437). OG6198> <OG11232 .(621). <OG5459 .(774). .(2906) OG1765> .(207). OG11232> .(101).
Vibrio navarrensis .(465). <OG5459 .(301). OG6198> .(256). .(2006) OG1765> .(654).
Vibrio ponticus .(658). OG5459> .(547). OG11232> .(74).. .(2153) <OG6198 .(81).. OG1765> .(541).
Vibrio qinghaiensis .(396). <OG5459 .(509). .(981). <OG6198 OG11232> .(538). <OG1765 .(921).
Vibrio rumoiensis .(141). OG5459> .(17).. <OG5459 .(248). <OG5459 .(226). <OG6198 OG11232> .(345). .(848). OG1765> .(1517)
Vibrio scophthalmi .(205). <OG6198 OG11232> .(48).. <OG5459 .(515). <OG5459 .(461). .(2045) OG1765> .(672).
Vibrio spartinae .(537). OG5459> .(454). .(979). OG1765> .(1481) OG6198> .(700).
Vibrio tapetis subsp. tapetis .(866). OG6198> .(21).. <OG5459 .(53).. OG5459> .(600). .(1680) OG11232> .(227). OG1765> .(1283)
Vibrio taketomensis .(608). OG5459> .(374). <OG6198 OG11232> .(64).. .(784). <OG1765 .(79).. OG6198> .(1623)
Vibrio tritonius .(1060) OG5459> .(205). OG6198> .(322). .(1736) OG6198> .(343). <OG1765 .(883).
Vibrio vulnificus .(545). <OG5459 .(855). .(1232) <OG6198 OG11232> .(981). <OG1765 .(696).
Allofrancisella frigidaquae .(722). OG5459> .(768).
Allofrancisella guangzhouensis .(152). OG11232> .(893). <OG5459 .(453).
Francisella adeliensis .(567). OG5459> .(64).. OG11232> .(1256)
Francisella frigiditurris .(1330) <OG5459 .(442).
Francisella halioticida .(891). OG5459> .(1288)
Francisella hispaniensis .(1064) <OG5459 .(495). OG11232> .(241).
Francisella marina .(1103) OG5459> .(723). OG11232> .(117).
Francisella noatunensis subs.. .(31).. OG11232> .(471). OG5459> .(858).
Francisella opportunistica .(863). <OG5459 .(572). OG11232> .(227).
Francisella orientalis LADL-.. .(821). <OG5459 .(590).
Francisella orientalis FNO12 .(801). <OG5459 .(636).
Francisella orientalis FNO24 .(802). <OG5459 .(633).
Francisella persica ATCC VR-.. .(741). OG5459> .(288). OG11232> .(55)..
Francisella philomiragia .(483). <OG5459 .(564). OG11232> .(744).
Francisella salina .(509). OG5459> .(716). OG11232> .(670).
Francisella uliginis .(113). OG11232> .(725). OG5459> .(1153)
Beggiatoa leptomitoformis
Hydrogenovibrio crunogenus X..
Hydrogenovibrio marinus
Hydrogenovibrio thermophilus
Methylophaga frappieri .(1580) OG5459> .(943).
Methylophaga nitratireducent.. .(2870) OG5459> .(37)..
Piscirickettsia salmonis .(125). OG5459> .(2833)
Thiomicrospira aerophila AL3
Thiomicrospira cyclica ALM1
Thiomicrorhabdus aquaedulcis
Thiomicrorhabdus indica
Thiosulfatimonas sediminis
Thiosulfativibrio zosterae
Alteromonas addita .(857). <OG5459 .(1222) OG1765> .(136). <OG6198 .(1632)
Alteromonas australica .(870). <OG5459 .(207). OG6198> .(1352) <OG1765 .(1230)
Alteromonas mediterranea .(1059) OG1765> .(1631) OG5459> .(1044)
Alteromonas naphthalenivorans .(370). <OG5459 .(1938) <OG1765 .(1869)
Alteromonas pelagimontana .(2729) <OG6198 .(95).. <OG5459 .(216). OG1765> .(612).
Alteromonas stellipolaris LM.. .(358). <OG5459 .(1717) OG6198> .(87).. <OG1765 .(1703)
Catenovulum sediminis .(2593) OG5459> .(616). <OG1765 .(157).
Glaciecola amylolytica .(1576) <OG5459 .(2225)
Glaciecola nitratireducens F.. .(1089) OG1765> .(1434) OG5459> .(893).
Hydrocarboniclastica marina .(1225) <OG5459 .(872). <OG1765 OG2439> .(1462)
Marinobacter adhaerens HP15 .(1777) <OG2439 OG1765> .(92).. <OG5459 .(1528) OG11232> .(615).
Marinobacter fonticola .(703). <OG5459 .(900). <OG5459 .(977). <OG1765 OG2439> .(377). OG11232> .(973).
Marinobacter hydrocarbonocla.. .(2294) OG5459> .(96).. <OG1765 OG2439> .(1189)
Marinobacter psychrophilus .(2429) OG5459> .(104). OG2439> .(116). OG11232> .(840).
Marinobacter salarius .(1450) <OG2439 OG1765> .(103). <OG5459 .(1558) OG11232> .(833).
Marinobacter salinus .(532). <OG2439 OG1765> .(100). <OG5459 .(1523) OG11232> .(1542)
Saliniradius amylolyticus .(571). <OG5459 .(501). OG1765> .(1625) OG6198> .(223).
Salinimonas lutimaris .(1088) OG1765> .(1102) <OG6198 .(105). OG11232> .(393). OG5459> .(867).
Salinimonas sediminis .(1091) OG1765> .(1741) OG5459> .(857).
Colwellia beringensis .(2782) OG11232> .(508). OG5459> .(113). <OG1765 .(401).
Colwellia psychrerythraea 34H .(350). <OG6198 OG11232> .(2382) <OG1765 .(811). OG5459> .(853).
Litorilituus sediminis .(843). <OG1765 .(2235) <OG5459 .(585).
Thalassotalea crassostreae .(2200) <OG1765 .(358). OG5459> .(691).
Ferrimonas balearica DSM 9799 .(766). <OG5459 .(1837) <OG1765 .(525). <OG6198 OG11232> .(632).
Idiomarina andamanensis .(174). OG1765> .(2026)
Idiomarina loihiensis L2TR .(995). <OG1765 .(1646)
Moritella marina ATCC 15381 .(1455) OG6198> .(46).. OG11232> .(507). <OG6198 .(602). <OG2439 OG1765> .(1247) OG5459> .(155).
Moritella yayanosii .(373). OG5459> .(1970) OG1765> .(567). <OG2439 .(852).
Parashewanella spongiae .(1225) <OG5459 .(213). OG1765> .(2713)

Parashewanella tropica | .(1115) OG1765> .(197). <OG6198 OG11232> .(1222) OG5459> .(1068) |

Shewanella algae | .(1271) OG11232> .(1279) <OG1765 .(463). OG5459> .(945). <OG6198 .(320). |

Shewanella amazonensis SB2B | .(915). <OG5459 .(1253) <OG1765 .(1492) |

Shewanella baltica OS678 | .(1123) <OG5459 .(1616) <OG1765 .(458). <OG6198 OG11232> .(1170) |

Shewanella bicestrii | .(975). <OG5459 .(1375) OG6198> .(206). <OG1765 .(1425) |

Shewanella decolorationis | .(406). <OG5459 .(1203) OG6198> .(1154) <OG1765 .(1258) |

Shewanella denitrificans OS217 | .(1525) OG1765> .(1193) OG5459> .(1045) |

Shewanella donghaensis | .(35).. <OG1765 .(2214) <OG11232 OG6198> .(63).. OG11232> .(243). <OG5459 .(823). <OG5459 .(165). <OG6198 .(457). |

Shewanella frigidimarina NCI.. | .(1799) <OG6198 OG11232> .(838). <OG1765 .(220). OG5459> .(1140) |

Shewanella halifaxensis HAW-.. | .(1120) <OG5459 .(59).. OG11232> .(1478) <OG1765 .(730). <OG6198 .(902). |

Shewanella japonica | .(927). <OG6198 OG11232> .(297). <OG5459 .(1446) <OG1765 .(1377) |

Shewanella khirikhana | .(1884) <OG6198 .(325). OG1765> .(1396) OG5459> .(426). |

Shewanella livingstonensis | .(863). <OG5459 .(267). OG1765> .(519). <OG6198 OG11232> .(260). OG5459> .(2092) |

Shewanella loihica PV-4 | .(1098) <OG5459 .(61).. OG11232> .(1270) <OG1765 .(1392) <OG6198 .(66).. |

Shewanella maritima | .(675). <OG1765 .(163). OG5459> .(997). <OG6198 .(1838) OG6198> .(99).. |

Shewanella marisflavi | .(306). OG6198> .(688). <OG5459 .(60).. OG11232> .(1218) <OG1765 .(1374) |

Shewanella oneidensis MR-1 | .(3170) <OG1765 .(403). OG5459> .(554). |

Shewanella pealeana ATCC 700.. | .(1066) <OG5459 .(60).. <OG6198 OG11232> .(1444) <OG1765 .(731). <OG6198 .(716). <OG6198 .(211). |

Shewanella piezotolerans WP3 | .(1200) <OG5459 .(512). OG1765> .(1616) OG6198> .(226). <OG6198 OG11232> .(831). |

Shewanella polaris | .(1828) OG11232> .(808). <OG1765 .(216). OG5459> .(1008) |

Shewanella psychrophila | .(1665) <OG1765 .(511). OG6198> .(59).. OG5459> .(2253) <OG6198 OG11232> .(782). |

Shewanella putrefaciens CN-32 | .(1024) <OG5459 .(1390) <OG1765 .(1523) |

Shewanella sediminis HAW-EB3 | .(167). OG6198> .(1023) <OG5459 .(57).. <OG6198 OG11232> .(389). OG1765> .(2109) <OG6198 OG11232> .(777). |

Shewanella violacea DSS12 | .(1304) OG1765> .(1694) OG5459> .(750). <OG6198 .(137). |

Shewanella woodyi ATCC 51908 | .(1269) <OG5459 .(60).. <OG6198 OG11232> .(1660) <OG1765 .(1877) |

Pseudoalteromonas agarivorans | .(531). <OG5459 .(413). OG1765> .(2120) |

Pseudoalteromonas aliena SW19 | .(90).. OG5459> .(371). | .(838). <OG1765 .(253). |

Pseudoalteromonas arctica A .. | .(1396) <OG6198 OG11232> .(913). <OG1765 .(482). OG5459> .(496). |

Pseudoalteromonas carrageeno.. | .(492). <OG5459 .(424). OG1765> .(410). <OG6198 OG11232> .(1793) |

Pseudoalteromonas donghaensis | .(968). OG1765> .(2141) OG5459> .(8)... |

Pseudoalteromonas espejiana .. | .(1050) OG1765> .(313). OG11232> .(456). OG5459> .(1379) |

Pseudoalteromonas issachenko.. | .(975). OG1765> .(1443) OG5459> .(551). |

Pseudoalteromonas luteoviola.. | .(209). <OG5459 .(743). OG1765> .(2747) |

Pseudoalteromonas paragorgic.. | .(34).. OG5459> .(602). | .(563). <OG6198 OG11232> .(8)... | .(296). <OG1765 .(374). |

Pseudoalteromonas phenolica | .(2323) <OG1765 .(312). OG5459> .(672). |

Pseudoalteromonas piratica | .(1299) <OG5459 .(119). OG1765> .(1413) |

Pseudoalteromonas prydzensis.. | .(372). OG5459> .(469). <OG6198 OG11232> .(19).. | .(14).. <OG1765 .(230). |

Pseudoalteromonas rubra | .(449). <OG5459 .(35).. | .(97).. <OG6198 .(911). | .(799). <OG1765 .(320). |

Pseudoalteromonas spongiae U.. | .(678). <OG5459 .(116). OG1765> .(1973) |

Pseudoalteromonas tetraodonis | .(989). OG1765> .(1447) OG5459> .(537). |

Pseudoalteromonas translucida | .(15).. OG11232> .(517). | .(889). OG1765> .(1489) OG5459> .(437). |

Pseudoalteromonas tunicata | .(1792) <OG1765 .(158). OG5459> .(1467) |

Pseudoalteromonas undina | .(318). <OG5459 .(328). | .(227). <OG5459 .(1422) <OG1765 .(540). |

Psychromonas ingrahamii 37 | .(86).. <OG5459 .(1931) <OG5459 .(1703) |

Aquicella lusitana | .(968). <OG1765 .(973). |

Aquicella siphonis | .(1302) OG1765> .(867). |

Coxiella burnetii RSA 493 | .(493). OG1765> .(1304) |

Fluoribacter dumoffii Tex-KL | .(3083) OG1765> .(41).. |

Legionella adelaidensis |

Legionella anisa | .(592). <OG1765 .(2928) |

Legionella clemsonensis | .(1996) OG1765> .(797). |

Legionella fallonii LLAP-10 | .(32).. <OG1765 .(3358) |

Legionella hackeliae | .(803). <OG1765 .(2136) |

Legionella israelensis | .(626). <OG1765 .(1956) |

Legionella lansingensis | .(1881) OG1765> .(763). |

Legionella pneumophila | .(1562) <OG1765 .(1396) |

Legionella sainthelensi |

Legionella spiritensis | .(244). <OG1765 .(2711) |

Legionella waltersii | .(1739) <OG1765 .(1559) |

Tatlockia micdadei | .(889). <OG1765 .(1823) <OG5459 .(138). |

Atlantibacter hermannii | .(1061) OG5459> .(112). <OG1765 .(68).. OG5459> .(552). <OG6198 .(1102) OG5459> .(954). |

Buttiauxella agrestis | .(1932) <OG6198 OG11232> .(990). <OG1765 .(67).. OG5459> .(494). <OG5459 .(605). |

Cedecea lapagei | .(757). OG5459> .(1375) <OG6198 OG11232> .(985). <OG1765 .(1146) |

Cedecea neteri | .(918). OG5459> .(1531) <OG6198 OG11232> .(1109) <OG1765 .(91).. OG5459> .(1101) |

Citrobacter amalonaticus | .(198). <OG6198 OG11232> .(1851) OG5459> .(2288) <OG1765 .(96).. OG5459> .(302). |

Citrobacter freundii | .(1096) <OG1765 .(555). <OG6198 OG11232> .(1504) OG5459> .(1385) |

Citrobacter portucalensis | .(950). <OG1765 .(500). <OG6198 OG11232> .(1566) OG5459> .(1459) |

Citrobacter werkmanii | .(264). <OG6198 OG11232> .(1474) OG5459> .(2484) <OG1765 .(303). |

Citrobacter rodentium ICC168 | .(254). <OG6198 .(1869) OG1765> .(313). <OG5459 .(2276) <OG5459 .(6)... |

Cronobacter condimenti 1330 | .(1075) <OG5459 .(161). OG1765> .(2048) <OG5459 .(176). <OG6198 .(449). |

Cronobacter dublinensis subs.. | .(1109) <OG5459 .(85).. OG1765> .(2214) <OG5459 .(173). <OG6198 .(456). |

Cronobacter malonaticus LMG .. | .(911). <OG5459 .(136). OG1765> .(2148) <OG5459 .(166). <OG6198 .(558). |

Cronobacter muytjensii ATCC .. | .(761). <OG5459 .(92).. OG1765> .(2179) <OG5459 .(172). <OG6198 .(717). |

Cronobacter sakazakii | .(235). <OG5459 .(133). OG1765> .(2118) <OG5459 .(170). <OG6198 .(1212) |

Cronobacter universalis NCTC.. | .(1092) <OG5459 .(82).. OG1765> .(2121) <OG5459 .(182). <OG6198 .(440). |

Enterobacter asburiae | .(817). OG6198> .(377). <OG5459 .(91).. OG1765> .(869). OG11232> .(1607) <OG5459 .(608). |

Enterobacter cancerogenus | .(288). <OG6198 .(1578) OG5459> .(1181) OG11232> .(1219) <OG1765 .(45).. OG5459> .(117). |

Enterobacter chengduensis | .(880). <OG11232 OG6198> .(378). <OG5459 .(84).. OG1765> .(935). OG11232> .(1632) <OG5459 .(740). |

Enterobacter cloacae | .(866). OG6198> .(380). <OG5459 .(48).. OG1765> .(994). OG11232> .(1499) <OG5459 .(651). |

Enterobacter ludwigii | .(270). <OG6198 OG11232> .(672). <OG5459 .(1066) <OG5459 .(70).. OG1765> .(2362) |

Enterobacter roggenkampii | .(841). OG6198> .(355). <OG5459 .(72).. OG1765> .(961). OG11232> .(1461) <OG5459 .(609). |

Enterobacter sichuanensis | .(817). OG6198> .(392). <OG5459 .(51).. OG1765> .(930). OG11232> .(1534) <OG5459 .(603). |

Enterobacter oligotrophicus | .(974). <OG1765 .(44).. OG5459> .(373). <OG6198 OG11232> .(1538) OG5459> .(1157) |

Enterobacter soli | .(613). OG5459> .(2408) <OG1765 .(107). OG5459> .(376). <OG6198 OG11232> .(907). |

Escherichia albertii | .(7)... OG5459> .(326). <OG6198 .(1922) <OG1765 .(78).. OG5459> .(1907) |

Escherichia coli O26 str. RM.. | .(7)... OG5459> .(324). <OG6198 .(2792) <OG1765 .(173). OG5459> .(685). <OG6198 .(1337) |

Escherichia coli O26 str. RM.. | .(7)... OG5459> .(346). <OG6198 .(2839) <OG1765 .(249). OG5459> .(729). <OG6198 .(1332) |

Escherichia coli O103 str. R.. | .(8)... OG5459> .(325). <OG6198 .(257). OG1765> .(2541) OG5459> .(691). <OG6198 .(1455) |

Escherichia coli O43 str. RM.. | .(8)... OG5459> .(280). <OG6198 .(2254) <OG1765 .(105). OG5459> .(1994) |

Escherichia coli O111 str. R.. | .(6)... OG5459> .(314). <OG6198 .(2336) <OG1765 .(89).. OG5459> .(728). <OG6198 .(1279) |

Escherichia coli O121 str. R.. | .(1306) <OG1765 .(166). OG5459> .(790). <OG6198 .(2411) OG6198> .(274). <OG5459 .(7)... |

Escherichia coli O145 str. R.. | .(7)... OG5459> .(277). <OG6198 .(2515) <OG1765 .(86).. OG5459> .(616). <OG6198 .(1386) |

Escherichia coli O157:H7 str..

Escherichia coli str. K-12 s.. | .(7)... <OG5459 .(296). <OG6198 .(1979) <OG1765 .(115). OG5459> .(1839) |

Escherichia fergusonii | .(219). <OG1765 .(73).. OG5459> .(253). OG5459> .(10).. OG6198> .(375). <OG5459 .(3313) |

Escherichia marmotae | .(555). OG5459> .(297). <OG6198 .(1836) <OG1765 .(134). OG5459> .(573). <OG6198 .(630). |

Klebsiella aerogenes | .(668). OG5459> .(2757) <OG1765 .(52).. OG5459> .(353). <OG6198 OG11232> .(878). |

Klebsiella huaxiensis | .(938). OG5459> .(1374) OG5459> .(873). <OG5459 .(26).. OG5459> .(800). <OG1765 .(566). <OG6198 OG11232> .(972). |

Klebsiella michiganensis | .(825). OG5459> .(3226) <OG1765 .(618). <OG6198 OG11232> .(909). |

Klebsiella pneumoniae subsp... | .(717). OG5459> .(3074) <OG1765 .(74).. OG5459> .(467). <OG6198 OG11232> .(979). |

Klebsiella quasipneumoniae | .(779). OG5459> .(2681) <OG1765 .(64).. OG5459> .(443). <OG6198 OG11232> .(855). |

Klebsiella variicola | .(709). OG5459> .(1183) OG5459> .(1876) <OG1765 .(95).. OG5459> .(442). <OG6198 OG11232> .(855). |

Kluyvera intermedia | .(610). OG5459> .(1651) OG11232> .(690). <OG1765 .(421). <OG6198 OG11232> .(823). |

Kosakonia arachidis | .(2136) <OG5459 .(1235) OG6198> .(141). OG11232> .(640). <OG5459 .(115). OG1765> .(388). |

Kosakonia cowanii | .(642). OG5459> .(2278) <OG1765 .(131). OG5459> .(633). <OG6198 .(537). |

Kosakonia oryzae | .(758). OG5459> .(2672) <OG1765 .(128). OG5459> .(828). <OG6198 .(574). |

Kosakonia pseudosacchari | .(283). <OG6198 .(312). OG6198> .(758). <OG5459 .(84).. OG1765> .(2428) <OG5459 .(641). |

Kosakonia radicincitans | .(461). OG5459> .(2730) <OG1765 .(123). OG5459> .(974). <OG6198 .(874). |

Kosakonia sacchari | .(251). <OG1765 .(91).. OG5459> .(1486) <OG6198 .(306). OG6198> .(173). OG5459> .(2189) |

Leclercia adecarboxylata | .(624). OG5459> .(2452) <OG1765 .(61).. OG5459> .(382). <OG6198 OG11232> .(818). |

Lelliottia amnigena | .(1925) <OG1765 .(45).. OG5459> .(345). <OG6198 OG11232> .(1422) OG5459> .(313). |

Lelliottia jeotgali | .(636). OG5459> .(2263) <OG1765 .(45).. OG5459> .(356). <OG6198 OG11232> .(904). |

Lelliottia nimipressuralis | .(769). <OG1765 .(74).. OG5459> .(369). <OG6198 OG11232> .(1487) OG5459> .(1692) |

Phytobacter diazotrophicus | .(255). OG5459> .(2129) <OG6198 OG11232> .(706). <OG1765 .(96).. OG5459> .(1770) |

Phytobacter ursingii | .(1539) OG5459> .(2122) <OG6198 OG11232> .(805). <OG1765 .(75).. OG5459> .(534). |

Pluralibacter gergoviae | .(514). <OG5459 .(53).. OG1765> .(2680) <OG5459 .(1637) |

Raoultella electrica | .(678). OG5459> .(1120) OG5459> .(1591) <OG1765 .(523). <OG6198 OG11232> .(794). |

Raoultella ornithinolytica | .(736). OG5459> .(1156) OG5459> .(1822) <OG1765 .(445). <OG6198 OG11232> .(877). |

Raoultella planticola | .(208). <OG1765 .(215). <OG5459 .(234). <OG6198 OG11232> .(1593) OG5459> .(2692) |

Raoultella terrigena | .(664). OG5459> .(2990) <OG1765 .(472). <OG6198 OG11232> .(913). |

Salmonella bongori | .(412). <OG1765 .(82).. OG5459> .(383). <OG6198 OG11232> .(1417) OG5459> .(1687) |

Salmonella enterica subsp. e.. | .(6)... OG5459> .(2390) <OG1765 .(76).. OG5459> .(407). <OG6198 OG11232> .(1690) |

Salmonella enterica subsp. e.. | .(6)... OG5459> .(2298) <OG1765 .(76).. OG5459> .(418). <OG6198 OG11232> .(1517) |

Salmonella enterica subsp. e.. | .(6)... OG5459> .(2313) <OG1765 .(76).. OG5459> .(526). <OG6198 OG11232> .(1520) |

Salmonella enterica subsp. e.. | .(6)... OG5459> .(2300) <OG1765 .(81).. OG5459> .(405). <OG6198 OG11232> .(1452) |

Scandinavium goeteborgense | .(573). OG5459> .(2481) <OG1765 .(54).. OG5459> .(564). <OG6198 .(546). |

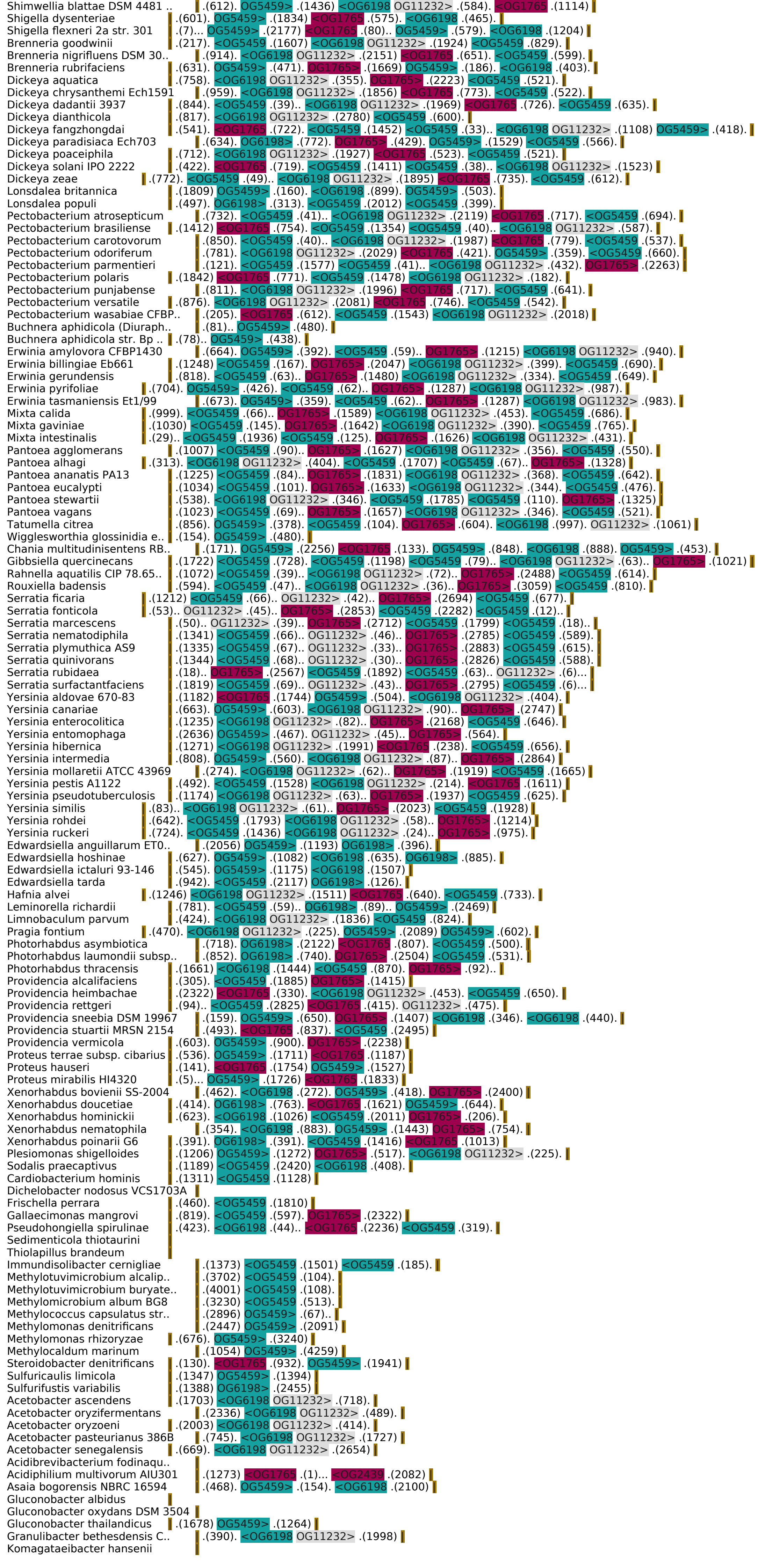

Shimwellia blattae DSM 4481 .. .(612). OG5459> .(1436) <OG6198 OG11232> .(584). <OG1765 .(1114)
Shigella dysenteriae .(601). OG5459> .(1834) <OG1765 .(575). <OG6198 .(465).
Shigella flexneri 2a str. 301 .(7)... OG5459> .(2177) <OG1765 .(80).. OG5459> .(579). <OG6198 .(1204)
Brenneria goodwinii .(217). <OG5459 .(1607) <OG6198 OG11232> .(1924) <OG5459 .(829).
Brenneria nigrifluens DSM 30.. .(914). <OG6198 OG11232> .(2151) <OG1765 .(651). <OG5459 .(599).
Brenneria rubrifaciens .(631). OG5459> .(471). OG1765> .(1669) OG5459> .(186). <OG6198 .(403).
Dickeya aquatica .(758). <OG6198 OG11232> .(355). OG1765> .(2223) <OG5459 .(521).
Dickeya chrysanthemi Ech1591 .(959). <OG6198 OG11232> .(1856) <OG1765 .(773). <OG5459 .(522).
Dickeya dadantii 3937 .(844). <OG5459 .(39).. <OG6198 OG11232> .(1969) <OG1765 .(726). <OG5459 .(635).
Dickeya dianthicola .(817). <OG6198 OG11232> .(2780) <OG5459 .(600).
Dickeya fangzhongdai .(541). <OG1765 .(722). <OG5459 .(1452) <OG5459 .(33).. <OG6198 OG11232> .(1108) OG5459> .(418).
Dickeya paradisiaca Ech703 .(634). OG6198> .(772). OG1765> .(429). OG5459> .(1529) <OG5459 .(566).
Dickeya poaceiphila .(712). <OG6198 OG11232> .(1927) <OG1765 .(523). <OG5459 .(521).
Dickeya solani IPO 2222 .(422). <OG1765 .(719). <OG5459 .(1411) <OG5459 .(38).. <OG6198 OG11232> .(1523)
Dickeya zeae .(772). <OG5459 .(49).. <OG6198 OG11232> .(1895) <OG1765 .(735). <OG5459 .(612).
Lonsdalea britannica .(1809) OG5459> .(160). <OG6198 .(899). OG5459> .(503).
Lonsdalea populi .(497). OG6198> .(313). <OG5459 .(2012) <OG5459 .(399).
Pectobacterium atrosepticum .(732). <OG5459 .(41).. <OG6198 OG11232> .(2119) <OG1765 .(717). <OG5459 .(694).
Pectobacterium brasiliense .(1412) <OG1765 .(754). <OG5459 .(1354) <OG5459 .(40).. <OG6198 OG11232> .(587).
Pectobacterium carotovorum .(850). <OG5459 .(40).. <OG6198 OG11232> .(1987) <OG1765 .(779). <OG5459 .(537).
Pectobacterium odoriferum .(781). <OG6198 OG11232> .(2029) <OG1765 .(421). OG5459> .(359). <OG5459 .(660).
Pectobacterium parmentieri .(121). <OG5459 .(1577) <OG5459 .(41).. <OG6198 OG11232> .(432). OG1765> .(2263)
Pectobacterium polaris .(1842) <OG1765 .(771). <OG5459 .(1478) <OG6198 OG11232> .(182).
Pectobacterium punjabense .(811). <OG6198 OG11232> .(1996) <OG1765 .(717). <OG5459 .(641).
Pectobacterium versatile .(876). <OG6198 OG11232> .(2081) <OG1765 .(746). <OG5459 .(542).
Pectobacterium wasabiae CFBP.. .(205). <OG1765 .(612). <OG5459 .(1543) <OG6198 OG11232> .(2018)
Buchnera aphidicola (Diuraph.. .(81).. OG5459> .(480).
Buchnera aphidicola str. Bp .. .(78).. OG5459> .(438).
Erwinia amylovora CFBP1430 .(664). OG5459> .(392). <OG5459 .(59).. OG1765> .(1215) <OG6198 OG11232> .(940).
Erwinia billingiae Eb661 .(1248) <OG5459 .(167). OG1765> .(2047) <OG6198 OG11232> .(399). <OG5459 .(690).
Erwinia gerundensis .(818). <OG5459 .(63).. OG1765> .(1480) <OG6198 OG11232> .(334). <OG5459 .(649).
Erwinia pyrifoliae .(704). OG5459> .(426). <OG5459 .(62).. OG1765> .(1287) <OG6198 OG11232> .(987).
Erwinia tasmaniensis Et1/99 .(673). OG5459> .(359). <OG5459 .(62).. OG1765> .(1287) <OG6198 OG11232> .(983).
Mixta calida .(999). <OG5459 .(66).. OG1765> .(1589) <OG6198 OG11232> .(453). <OG5459 .(686).
Mixta gaviniae .(1030) <OG5459 .(145). OG1765> .(1642) <OG6198 OG11232> .(390). <OG5459 .(765).
Mixta intestinalis .(29).. <OG5459 .(1936) <OG5459 .(125). OG1765> .(1626) <OG6198 OG11232> .(431).
Pantoea agglomerans .(1007) <OG5459 .(90).. OG1765> .(1627) <OG6198 OG11232> .(356). <OG5459 .(550).
Pantoea alhagi .(313). <OG6198 OG11232> .(404). <OG5459 .(1707) <OG5459 .(67).. OG1765> .(1328)
Pantoea ananatis PA13 .(1225) <OG5459 .(84).. OG1765> .(1831) <OG6198 OG11232> .(368). <OG5459 .(642).
Pantoea eucalypti .(1034) <OG5459 .(101). OG1765> .(1633) <OG6198 OG11232> .(344). <OG5459 .(476).
Pantoea stewartii .(538). <OG6198 OG11232> .(346). <OG5459 .(1785) <OG5459 .(110). OG1765> .(1325)
Pantoea vagans .(1023) <OG5459 .(69).. OG1765> .(1657) <OG6198 OG11232> .(346). <OG5459 .(521).
Tatumella citrea .(856). OG5459> .(378). <OG5459 .(104). OG1765> .(604). <OG6198 .(997). OG11232> .(1061)
Wigglesworthia glossinidia e.. .(154). OG5459> .(480).
Chania multitudinisentens RB.. .(171). OG5459> .(2256) <OG1765 .(133). OG5459> .(848). <OG6198 .(888). OG5459> .(453).
Gibbsiella quercinecans .(1722) <OG5459 .(728). <OG5459 .(79).. <OG6198 OG11232> .(63).. OG1765> .(1021)
Rahnella aquatilis CIP 78.65.. .(1072) <OG5459 .(39).. <OG6198 OG11232> .(72).. OG1765> .(2488) <OG5459 .(614).
Rouxiella badensis .(594). <OG5459 .(47).. <OG6198 OG11232> .(36).. OG1765> .(3059) <OG5459 .(810).
Serratia ficaria .(1212) <OG5459 .(66).. OG11232> .(42).. OG1765> .(2694) <OG5459 .(677).
Serratia fonticola .(53).. OG11232> .(45).. OG1765> .(2853) <OG5459 .(2282) <OG5459 .(12)..
Serratia marcescens .(50).. OG11232> .(39).. OG1765> .(2712) <OG5459 .(1799) <OG5459 .(18)..
Serratia nematodiphila .(1341) <OG5459 .(66).. OG11232> .(46).. OG1765> .(2785) <OG5459 .(589).
Serratia plymuthica AS9 .(1335) <OG5459 .(67).. OG11232> .(33).. OG1765> .(2883) <OG5459 .(615).
Serratia quinivorans .(1344) <OG5459 .(68).. OG11232> .(30).. OG1765> .(2826) <OG5459 .(588).
Serratia rubidaea .(18).. OG1765> .(2567) <OG5459 .(1892) <OG5459 .(63).. OG11232> .(6)...
Serratia surfactantfaciens .(1819) <OG5459 .(69).. OG11232> .(43).. OG1765> .(2795) <OG5459 .(6)...
Yersinia aldovae 670-83 .(1182) <OG1765 .(1744) OG5459> .(504). <OG6198 OG11232> .(404).
Yersinia canariae .(663). OG5459> .(603). <OG6198 OG11232> .(90).. OG1765> .(2747)
Yersinia enterocolitica .(1235) <OG6198 OG11232> .(82).. OG1765> .(2168) <OG5459 .(646).
Yersinia entomophaga .(2636) OG5459> .(467). OG11232> .(45).. OG1765> .(564).
Yersinia hibernica .(1271) <OG6198 OG11232> .(1991) <OG1765 .(238). <OG5459 .(656).
Yersinia intermedia .(808). OG5459> .(560). <OG6198 OG11232> .(87).. OG1765> .(2864)
Yersinia mollaretii ATCC 43969 .(274). <OG6198 OG11232> .(62).. OG1765> .(1919) <OG5459 .(1665)
Yersinia pestis A1122 .(492). <OG5459 .(1528) <OG6198 OG11232> .(214). <OG1765 .(1611)
Yersinia pseudotuberculosis .(1174) <OG6198 OG11232> .(63).. OG1765> .(1937) <OG5459 .(625).
Yersinia similis .(83).. <OG6198 OG11232> .(61).. OG1765> .(2023) <OG5459 .(1928)
Yersinia rohdei .(642). <OG5459 .(1793) <OG6198 OG11232> .(58).. OG1765> .(1214)
Yersinia ruckeri .(724). <OG5459 .(1436) <OG6198 OG11232> .(24).. OG1765> .(975).
Edwardsiella anguillarum ET0.. .(2056) OG5459> .(1193) OG6198> .(396).
Edwardsiella hoshinae .(627). OG5459> .(1082) <OG6198 .(635). OG6198> .(885).
Edwardsiella ictaluri 93-146 .(545). OG5459> .(1175) <OG6198 .(1507)
Edwardsiella tarda .(942). <OG5459 .(2117) OG6198> .(126).
Hafnia alvei .(1246) <OG6198 OG11232> .(1511) <OG1765 .(640). <OG5459 .(733).
Leminorella richardii .(781). <OG5459 .(59).. OG6198> .(89).. OG5459> .(2469)
Limnobaculum parvum .(424). <OG6198 OG11232> .(1836) <OG5459 .(824).
Pragia fontium .(470). <OG6198 OG11232> .(225). OG5459> .(2089) OG5459> .(602).
Photorhabdus asymbiotica .(718). OG6198> .(2122) <OG1765 .(807). <OG5459 .(500).
Photorhabdus laumondii subsp.. .(852). OG6198> .(740). OG1765> .(2504) <OG5459 .(531).
Photorhabdus thracensis .(1661) <OG6198 .(1444) <OG5459 .(870). OG1765> .(92)..
Providencia alcalifaciens .(305). <OG5459 .(1885) OG1765> .(1415)
Providencia heimbachae .(2322) <OG1765 .(330). <OG6198 OG11232> .(453). <OG5459 .(650).
Providencia rettgeri .(94).. <OG5459 .(2825) <OG1765 .(415). OG11232> .(475).
Providencia sneebia DSM 19967 .(159). OG5459> .(650). OG1765> .(1407) <OG6198 .(346). <OG6198 .(440).
Providencia stuartii MRSN 2154 .(493). <OG1765 .(837). <OG5459 .(2495)
Providencia vermicola .(603). OG5459> .(900). OG1765> .(2238)
Proteus terrae subsp. cibarius .(536). OG5459> .(1711) <OG1765 .(1187)
Proteus hauseri .(141). <OG1765 .(1754) OG5459> .(1527)
Proteus mirabilis HI4320 .(5)... OG5459> .(1726) <OG1765 .(1833)
Xenorhabdus bovienii SS-2004 .(462). <OG6198 .(272). OG5459> .(418). OG1765> .(2400)
Xenorhabdus doucetiae .(414). OG6198> .(763). <OG1765 .(1621) OG5459> .(644).
Xenorhabdus hominickii .(623). <OG6198 .(1026) <OG5459 .(2011) OG1765> .(206).
Xenorhabdus nematophila .(354). <OG6198 .(883). OG5459> .(1443) OG1765> .(754).
Xenorhabdus poinarii G6 .(391). OG6198> .(391). <OG5459 .(1416) <OG1765 .(1013)
Plesiomonas shigelloides .(1206) OG5459> .(1272) OG1765> .(517). <OG6198 OG11232> .(225).
Sodalis praecaptivus .(1189) <OG5459 .(2420) <OG6198 .(408).
Cardiobacterium hominis .(1311) <OG5459 .(1128)
Dichelobacter nodosus VCS1703A
Frischella perrara .(460). <OG5459 .(1810)
Gallaecimonas mangrovi .(819). <OG5459 .(597). OG1765> .(2322)
Pseudohongiella spirulinae .(423). <OG6198 .(44).. <OG1765 .(2236) <OG5459 .(319).
Sedimenticola thiotaurini
Thiolapillus brandeum
Immundisolibacter cernigliae .(1373) <OG5459 .(1501) <OG5459 .(185).
Methylotuvimicrobium alcalip.. .(3702) <OG5459 .(104).
Methylotuvimicrobium buryate.. .(4001) <OG5459 .(108).
Methylomicrobium album BG8 .(3230) <OG5459 .(513).
Methylococcus capsulatus str.. .(2896) OG5459> .(67)..
Methylomonas denitrificans .(2447) OG5459> .(2091)
Methylomonas rhizoryzae .(676). OG5459> .(3240)
Methylocaldum marinum .(1054) OG5459> .(4259)
Steroidobacter denitrificans .(130). <OG1765 .(932). OG5459> .(1941)
Sulfuricaulis limicola .(1347) OG5459> .(1394)
Sulfurifustis variabilis .(1388) OG6198> .(2455)
Acetobacter ascendens .(1703) <OG6198 OG11232> .(718).
Acetobacter oryzifermentans .(2336) <OG6198 OG11232> .(489).
Acetobacter oryzoeni .(2003) <OG6198 OG11232> .(414).
Acetobacter pasteurianus 386B .(745). <OG6198 OG11232> .(1727)
Acetobacter senegalensis .(669). <OG6198 OG11232> .(2654)
Acidibrevibacterium fodinaqu..
Acidiphilium multivorum AIU301 .(1273) <OG1765 .(1)... <OG2439 .(2082)
Asaia bogorensis NBRC 16594 .(468). OG5459> .(154). <OG6198 .(2100)
Gluconobacter albidus
Gluconobacter oxydans DSM 3504
Gluconobacter thailandicus .(1678) OG5459> .(1264)
Granulibacter bethesdensis C.. .(390). <OG6198 OG11232> .(1998)
Komagataeibacter hansenii

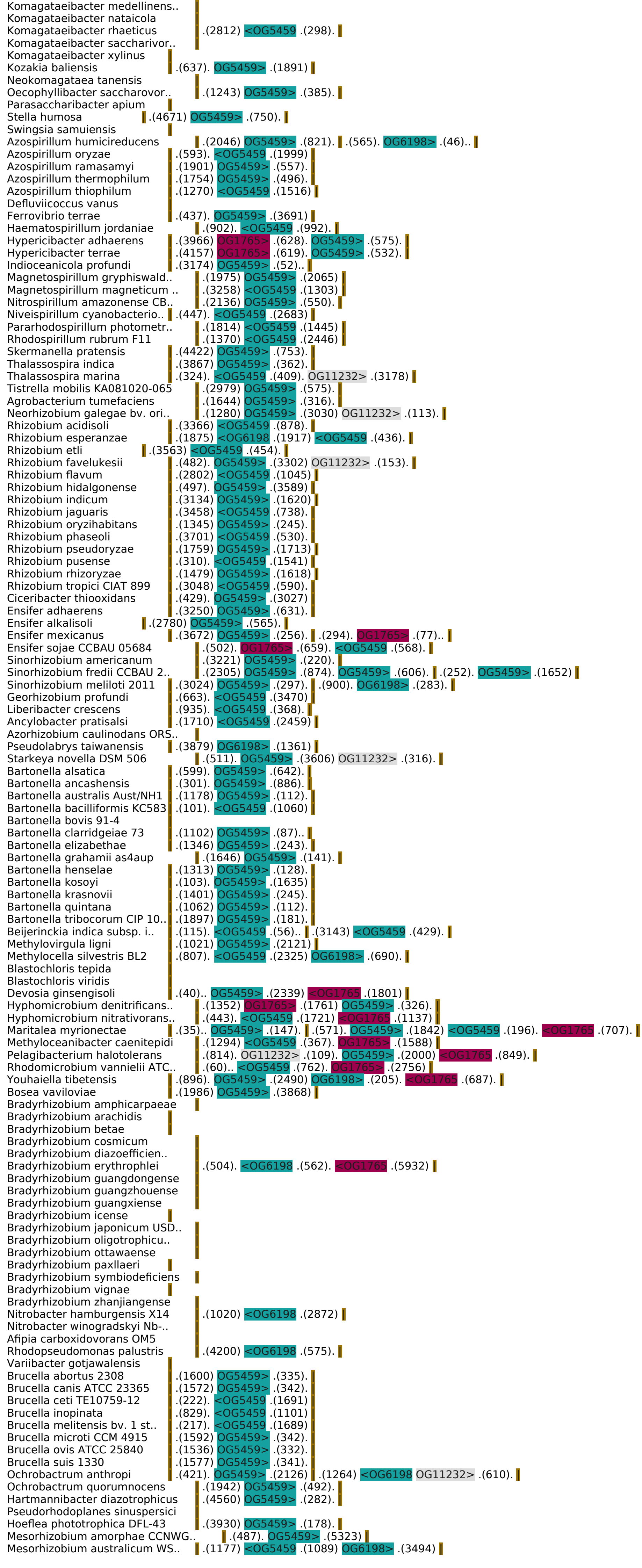
Komagataeibacter medellinens..
Komagataeibacter nataicola
Komagataeibacter rhaeticus .(2812) <OG5459 .(298).
Komagataeibacter saccharivor..
Komagataeibacter xylinus
Kozakia baliensis .(637). OG5459> .(1891)
Neokomagataea tanensis
Oecophyllibacter saccharovor.. .(1243) OG5459> .(385).
Parasaccharibacter apium
Stella humosa .(4671) OG5459> .(750).
Swingsia samuiensis
Azospirillum humicireducens .(2046) OG5459> .(821). .(565). OG6198> .(46)..
Azospirillum oryzae .(593). <OG5459 .(1999)
Azospirillum ramasamyi .(1901) OG5459> .(557).
Azospirillum thermophilum .(1754) OG5459> .(496).
Azospirillum thiophilum .(1270) <OG5459 .(1516)
Defluviicoccus vanus
Ferrovibrio terrae .(437). OG5459> .(3691)
Haematospirillum jordaniae .(902). <OG5459 .(992).
Hypericibacter adhaerens .(3966) OG1765> .(628). OG5459> .(575).
Hypericibacter terrae .(4157) OG1765> .(619). OG5459> .(532).
Indioceanicola profundi .(3174) OG5459> .(52)..
Magnetospirillum gryphiswald.. .(1975) OG5459> .(2065)
Magnetospirillum magneticum .. .(3258) <OG5459 .(1303)
Nitrospirillum amazonense CB.. .(2136) OG5459> .(550).
Niveispirillum cyanobacterio.. .(447). <OG5459 .(2683)
Pararhodospirillum photometr.. .(1814) <OG5459 .(1445)
Rhodospirillum rubrum F11 .(1370) <OG5459 .(2446)
Skermanella pratensis .(4422) OG5459> .(753).
Thalassospira indica .(3867) OG5459> .(362).
Thalassospira marina .(324). <OG5459 .(409). OG11232> .(3178)
Tistrella mobilis KA081020-065 .(2979) OG5459> .(575).
Agrobacterium tumefaciens .(1644) OG5459> .(316).
Neorhizobium galegae bv. ori.. .(1280) OG5459> .(3030) OG11232> .(113).
Rhizobium acidisoli .(3366) <OG5459 .(878).
Rhizobium esperanzae .(1875) <OG6198 .(1917) <OG5459 .(436).
Rhizobium etli .(3563) <OG5459 .(454).
Rhizobium favelukesii .(482). OG5459> .(3302) OG11232> .(153).
Rhizobium flavum .(2802) <OG5459 .(1045)
Rhizobium hidalgonense .(497). OG5459> .(3589)
Rhizobium indicum .(3134) OG5459> .(1620)
Rhizobium jaguaris .(3458) <OG5459 .(738).
Rhizobium oryzihabitans .(1345) OG5459> .(245).
Rhizobium phaseoli .(3701) <OG5459 .(530).
Rhizobium pseudoryzae .(1759) OG5459> .(1713)
Rhizobium pusense .(310). <OG5459 .(1541)
Rhizobium rhizoryzae .(1479) OG5459> .(1618)
Rhizobium tropici CIAT 899 .(3048) <OG5459 .(590).
Ciceribacter thiooxidans .(429). OG5459> .(3027)
Ensifer adhaerens .(3250) OG5459> .(631).
Ensifer alkalisoli .(2780) OG5459> .(565).
Ensifer mexicanus .(3672) OG5459> .(256). .(294). OG1765> .(77)..
Ensifer sojae CCBAU 05684 .(502). OG1765> .(659). <OG5459 .(568).
Sinorhizobium americanum .(3221) OG5459> .(220).
Sinorhizobium fredii CCBAU 2.. .(2305) OG5459> .(874). OG5459> .(606). .(252). OG5459> .(1652)
Sinorhizobium meliloti 2011 .(3024) OG5459> .(297). .(900). OG6198> .(283).
Georhizobium profundi .(663). <OG5459 .(3470)
Liberibacter crescens .(935). <OG5459 .(368).
Ancylobacter pratisalsi .(1710) <OG5459 .(2459)
Azorhizobium caulinodans ORS..
Pseudolabrys taiwanensis .(3879) OG6198> .(1361)
Starkeya novella DSM 506 .(511). OG5459> .(3606) OG11232> .(316).
Bartonella alsatica .(599). OG5459> .(642).
Bartonella ancashensis .(301). OG5459> .(886).
Bartonella australis Aust/NH1 .(1178) OG5459> .(112).
Bartonella bacilliformis KC583 .(101). <OG5459 .(1060)
Bartonella bovis 91-4
Bartonella clarridgeiae 73 .(1102) OG5459> .(87)..
Bartonella elizabethae .(1346) OG5459> .(243).
Bartonella grahamii as4aup .(1646) OG5459> .(141).
Bartonella henselae .(1313) OG5459> .(128).
Bartonella kosoyi .(103). OG5459> .(1635)
Bartonella krasnovii .(1401) OG5459> .(245).
Bartonella quintana .(1062) OG5459> .(112).
Bartonella tribocorum CIP 10.. .(1897) OG5459> .(181).
Beijerinckia indica subsp. i.. .(115). <OG5459 .(56).. .(3143) <OG5459 .(429).
Methylovirgula ligni .(1021) OG5459> .(2121)
Methylocella silvestris BL2 .(807). <OG5459 .(2325) OG6198> .(690).
Blastochloris tepida
Blastochloris viridis
Devosia ginsengisoli .(40).. OG5459> .(2339) <OG1765 .(1801)
Hyphomicrobium denitrificans.. .(1352) OG1765> .(1761) OG5459> .(326).
Hyphomicrobium nitrativorans.. .(443). <OG5459 .(1721) <OG1765 .(1137)
Maritalea myrionectae .(35).. OG5459> .(147). .(571). OG5459> .(1842) <OG5459 .(196). <OG1765 .(707).
Methyloceanibacter caenitepidi .(1294) <OG5459 .(367). OG1765> .(1588)
Pelagibacterium halotolerans .(814). OG11232> .(109). OG5459> .(2000) <OG1765 .(849).
Rhodomicrobium vannielii ATC.. .(60).. <OG5459 .(762). OG1765> .(2756)
Youhaiella tibetensis .(896). OG5459> .(2490) OG6198> .(205). <OG1765 .(687).
Bosea vaviloviae .(1986) OG5459> .(3868)
Bradyrhizobium amphicarpaeae
Bradyrhizobium arachidis
Bradyrhizobium betae
Bradyrhizobium cosmicum
Bradyrhizobium diazoefficien..
Bradyrhizobium erythrophlei .(504). <OG6198 .(562). <OG1765 .(5932)
Bradyrhizobium guangdongense
Bradyrhizobium guangzhouense
Bradyrhizobium guangxiense
Bradyrhizobium icense
Bradyrhizobium japonicum USD..
Bradyrhizobium oligotrophicu..
Bradyrhizobium ottawaense
Bradyrhizobium paxllaeri
Bradyrhizobium symbiodeficiens
Bradyrhizobium vignae
Bradyrhizobium zhanjiangense
Nitrobacter hamburgensis X14 .(1020) <OG6198 .(2872)
Nitrobacter winogradskyi Nb-..
Afipia carboxidovorans OM5
Rhodopseudomonas palustris .(4200) <OG6198 .(575).
Variibacter gotjawalensis
Brucella abortus 2308 .(1600) OG5459> .(335).
Brucella canis ATCC 23365 .(1572) OG5459> .(342).
Brucella ceti TE10759-12 .(222). <OG5459 .(1691)
Brucella inopinata .(829). <OG5459 .(1101)
Brucella melitensis bv. 1 st.. .(217). <OG5459 .(1689)
Brucella microti CCM 4915 .(1592) OG5459> .(342).
Brucella ovis ATCC 25840 .(1536) OG5459> .(332).
Brucella suis 1330 .(1577) OG5459> .(341).
Ochrobactrum anthropi .(421). OG5459> .(2126) .(1264) <OG6198 OG11232> .(610).
Ochrobactrum quorumnocens .(1942) OG5459> .(492).
Hartmannibacter diazotrophicus .(4560) OG5459> .(282).
Pseudorhodoplanes sinuspersici
Hoeflea phototrophica DFL-43 .(3930) OG5459> .(178).
Mesorhizobium amorphae CCNWG.. .(487). OG5459> .(5323)
Mesorhizobium australicum WS.. .(1177) <OG5459 .(1089) OG6198> .(3494)

Mesorhizobium ciceri biovar .. .(5401) OG5459> .(471).

Mesorhizobium erdmanii .(1161) <OG5459 .(1276) OG6198> .(3676)

Mesorhizobium huakuii .(2227) OG5459> .(4082) | .(104). OG6198> .(98)..

Mesorhizobium jarvisii .(1152) <OG5459 .(5363)

Mesorhizobium japonicum MAFF.. .(1867) <OG6198 .(1242) OG5459> .(3436)

Mesorhizobium oceanicum .(267). <OG5459 .(4666)

Mesorhizobium opportunistum .. .(1176) <OG5459 .(5279)

Mesorhizobium terrae .(1961) <OG5459 .(1211) OG6198> .(2199)

Oricola thermophila .(1822) OG11232> .(1463) <OG5459 .(516).

Phyllobacterium zundukense .(44).. <OG5459 .(2832) <OG6198 .(894).

Roseitalea porphyridii .(3253) OG5459> .(142).

Salaquimonas pukyongi .(2821) OG5459> .(286).

Labrenzia alexandrii DFL-11 .(3086) OG5459> .(1746)

Stappia indica .(3043) <OG5459 .(1526)

Lichenihabitans psoromatis .(1882) <OG5459 .(2314)

Martelella endophytica .(994). OG5459> .(3414)

Martelella mediterranea DSM .. .(1940) OG11232> .(1376) OG5459> .(908).

Methylobacterium brachiatum .(3942) OG5459> .(1688)

Methylobacterium currus

Methylobacterium durans .(5453) OG5459> .(396).

Methylobacterium mesophilicu.. .(3827) <OG5459 .(2197)

Methylobacterium nodulans OR..

Methylobacterium oryzae CBMB20

Methylobacterium phyllosphae..

Methylobacterium radiotolera.. .(2267) OG5459> .(3392) | .(67).. OG6198> .(420).

Methylobacterium terrae .(5383) <OG6198 OG11232> .(104).

Methylorubrum extorquens PA1 .(1000) <OG6198 OG11232> .(3838)

Methylorubrum populi .(4595) <OG6198 .(311).

Microvirga ossetica .(2031) <OG5459 .(3237) | .(194). OG11232> .(321).

Microvirga thermotolerans .(576). OG5459> .(2959)

Methylocystis bryophila .(1465) <OG5459 .(2608)

Methylocystis heyeri

Methylocystis parvus

Parvibaculum lavamentivorans.. .(361). OG1765> .(1102) <OG5459 .(2200)

Altererythrobacter atlanticus .(908). <OG5459 .(2227)

Altererythrobacter epoxidivo.. .(1595) <OG5459 .(1128)

Altererythrobacter ishigakie.. .(1170) OG5459> .(60).. OG6198> .(1296)

Aurantiacibacter atlanticus .(134). OG5459> .(2683)

Croceicoccus marinus .(1211) OG5459> .(1676) | .(371). OG1765> .(526).

Erythrobacter aureus .(2402) <OG5459 .(388).

Erythrobacter litoralis .(1074) OG5459> .(1969)

Erythrobacter mangrovi .(464). <OG5459 .(2492)

Erythrobacter neustonensis .(82).. <OG5459 .(2688) <OG6198 .(68)..

Paraurantiacibacter namhicola .(2386) OG5459> .(127).

Pelagerythrobacter marensis .(1488) <OG5459 .(1195)

Qipengyuania flava .(2233) <OG5459 .(459).

Qipengyuania sediminis .(333). OG5459> .(1986)

Qipengyuania seohaensis .(288). <OG6198 .(2082) OG5459> .(486).

Tsuneonella amylolytica .(317). <OG5459 .(2362)

Tsuneonella dongtanensis .(1772) OG5459> .(1118)

Tsuneonella mangrovi .(881). <OG5459 .(1689)

Blastomonas fulva .(3335) <OG5459 .(310).

Novosphingobium aromaticivor.. .(2392) <OG5459 .(974).

Novosphingobium ginsenosidim.. .(2862) <OG5459 .(168).

Novosphingobium pentaromativ.. .(497). OG5459> .(1248) OG6198> .(1907)

Parasphingopyxis algicola .(1120) <OG5459 .(2372)

Rhizorhabdus dicambivorans .(2748) <OG5459 .(1882)

Sphingopyxis alaskensis RB2256 .(111). OG1765> .(1686) OG5459> .(1378)

Sphingopyxis fribergensis .(3155) OG5459> .(1453)

Sphingopyxis lindanitolerans .(1225) OG5459> .(1894) <OG5459 .(731).

Sphingopyxis macrogoltabida .(450). <OG5459 .(4499)

Sphingomonas alpina .(1583) OG5459> .(374). OG1765> .(2706)

Sphingomonas daechungensis

Sphingomonas ginsengisoli An.. .(2887) OG5459> .(41)..

Sphingomonas hengshuiensis .(4458) OG5459> .(240).

Sphingomonas lacunae .(1595) <OG5459 .(1183)

Sphingomonas lutea .(1131) OG5459> .(1224)

Sphingomonas melonis .(2589) OG5459> .(781).

Sphingomonas paucimobilis .(1630) <OG5459 .(2022)

Sphingomonas panacis .(492). <OG5459 .(4025)

Sphingomonas rhizophila .(1174) <OG5459 .(1105)

Sphingomonas sanxanigenens D.. .(1727) OG5459> .(3945)

Sphingomonas sediminicola .(865). OG5459> .(1555)

Sphingomonas taxi .(1141) <OG5459 .(2365)

Sphingomonas wittichii RW1 .(221). OG5459> .(4726)

Sphingobium barthaii .(323). OG5459> .(2850)

Sphingobium cloacae .(2576) OG5459> .(1012)

Sphingobium herbicidovorans .(859). <OG5459 .(1930)

Sphingobium hydrophobicum .(1877) OG5459> .(970).

Sphingobium indicum B90A .(281). OG5459> .(3148)

Sphingobium japonicum UT26S .(2511) <OG5459 .(785).

Allosphingosinicella indica .(2490) <OG5459 .(252).

Sphingorhabdus lacus .(1948) <OG5459 .(1284) OG5459>

Tardibacter chloracetimidivo.. .(1763) <OG5459 .(1633)

Zymomonas mobilis subsp. mob..

Sphingosinicella microcystin.. .(1492) OG5459> .(440). OG6198> .(1796)

Anaplasma centrale str. Israel .(484). OG5459> .(414).

Anaplasma marginale str. Flo.. .(434). <OG5459 .(472).

Anaplasma ovis str. Haibei .(409). <OG5459 .(501).

Anaplasma phagocytophilum st.. .(392). <OG5459 .(629).

Anaplasma platys .(361). <OG5459 .(515).

Ehrlichia canis str. Jake .(447). OG5459> .(486).

Ehrlichia chaffeensis str. W.. .(448). OG5459> .(436).

Ehrlichia muris AS145 .(415). <OG5459 .(452).

Ehrlichia ruminantium .(469). <OG5459 .(443).

Neorickettsia findlayensis .(586). <OG5459 .(150).

Neorickettsia helminthoeca s.. .(611). <OG5459 .(173).

Neorickettsia risticii str. .. .(595). <OG5459 .(163).

Neorickettsia sennetsu str. .. .(581). <OG5459 .(161).

Wolbachia pipientis .(164). OG5459> .(855).

Orientia tsutsugamushi

Rickettsia akari str. Hartford

Rickettsia asiatica

Rickettsia australis str. Cu..

Rickettsia conorii str. Mali..

Rickettsia helvetica C9P9

Rickettsia heilongjiangensis

Rickettsia japonica

Rickettsia monacensis

Rickettsia raoultii

Rickettsia rickettsii str. I..

Rickettsia sibirica 246

Rickettsia slovaca 13-B

Rickettsia bellii RML369-C

Rickettsia canadensis str. C..

Rickettsia prowazekii str. C..

Rickettsia typhi str. TH1527

Asticcacaulis excentricus CB.. .(105). <OG5459 .(2213)

Brevundimonas subvibrioides .. .(554). <OG5459 .(2751)

Brevundimonas vancanneytii .(139). <OG5459 .(2925)

Brevundimonas vesicularis .(1196) OG5459> .(623). OG1765> .(1521)

Caulobacter flavus .(1265) <OG5459 .(2292) <OG1765 .(1)... <OG2439 .(1507)

Caulobacter mirabilis .(324). OG5459> .(414). OG1765> .(1589) <OG6198 OG11232> .(1954)

Caulobacter rhizosphaerae .(4552) <OG1765 .(401). <OG5459 .(171).

Caulobacter segnis .(168). OG5459> .(458). <OG6198 .(3223) <OG1765 .(389).

Caulobacter vibrioides NA1000 .(841). <OG6198 .(2893) <OG5459 .(150).

Phenylobacterium zucineum HLK1 | .(3662) OG5459> .(215). |
Terricaulis silvestris | .(522). OG6198> .(2795) OG5459> .(557). |
Celeribacter ethanolicus | .(3423) <OG5459 .(467). |
Celeribacter indicus | .(1169) <OG5459 .(3188) |
Celeribacter marinus | .(2579) OG5459> .(419). |
Celeribacter manganoxidans | .(441). OG5459> .(2524) |
Defluviimonas alba | .(270). <OG5459 .(3932) |
Dinoroseobacter shibae DFL 1.. | .(1926) OG5459> .(1669) |
Epibacterium mobile F1926 | .(907). OG6198> .(235). | .(802). <OG5459 .(1389) OG11232> .(839). |
Haematobacter massiliensis | .(266). <OG5459 .(2105) |
Halocynthiibacter arcticus | .(637). OG11232> .(1143) OG6198> .(1576) <OG5459 .(485). |
Ketogulonicigenium robustum | .(1394) OG5459> .(941). |
Ketogulonicigenium vulgare | .(1631) OG5459> .(1043) |
Leisingera aquaemixtae | .(446). <OG5459 .(3156) | .(14).. OG6198> .(140). |
Leisingera methylohalidivora.. | .(827). OG6198> .(426). <OG5459 .(1259) OG11232> .(1309) |
Marinovum algicola DG 898 | .(101). <OG5459 .(102). | .(136). OG5459> .(41).. | .(73).. <OG5459 .(23).. | .(86).. OG11232> .(49).. |
Octadecabacter antarcticus 307 | .(798). <OG5459 .(3526) |
Octadecabacter arcticus 238 | .(3007) OG5459> .(1578) |
Octadecabacter temperatus | .(1664) OG11232> .(862). OG5459> .(677). |
Paracoccus aminophilus JCM 7.. | .(329). <OG5459 .(3137) | .(185). <OG6198 .(80).. |
Paracoccus aminovorans | .(452). <OG5459 .(2502) | .(34).. <OG6198 OG11232> .(584). |
Paracoccus contaminans | .(708). <OG5459 .(1044) OG11232> .(951). |
Paracoccus denitrificans | .(1171) OG5459> .(628). <OG6198 OG11232> .(982). |
Paracoccus jeotgali | .(1612) OG5459> .(1270) |
Paracoccus kondratievae | .(111). <OG6198 OG11232> .(182). <OG5459 .(1877) |
Paracoccus liaowanqingii | .(167). <OG5459 .(112). |
Paracoccus mutanolyticus | .(1386) <OG5459 .(1120) |
Paracoccus yeei | .(20).. <OG6198 OG11232> .(260). | .(1983) <OG5459 .(129). <OG1765 .(1325) |
Paracoccus zhejiangensis | .(3460) <OG5459 .(326). |
Parasedimentitalea marina | .(43).. OG11232> .(3269) OG5459> .(803). |
Paraoceanicella profunda | .(2119) OG11232> .(505). <OG5459 .(873). |
Pelagibaca abyssi | .(957). OG5459> .(3103) |
Phaeobacter gallaeciensis | .(2663) OG5459> .(782). |
Phaeobacter inhibens | .(513). <OG5459 .(2912) |
Phaeobacter porticola | .(2622) OG5459> .(729). |
Planktomarina temperata RCA23 | .(922). <OG5459 .(2179) |
Polymorphum gilvum SL003B-26A1 | .(2487) <OG6198 .(1717) OG5459> .(114). |
Profundibacter amoris | .(2248) OG5459> .(1234) |
Pseudohalocynthiibacter aest.. | .(706). OG5459> .(26).. OG1765> .(2023) OG11232> .(853). <OG6198 OG11232> .(37).. |
Pseudooceanicola algae | .(118). <OG5459 .(3299) |
Pseudopuniceibacterium antar.. | .(1986) <OG5459 .(375). OG6198> .(362). OG11232> .(1257) |
Rhodobaca barguzinensis | .(2759) OG5459> .(858). |
Rhodobacter blasticus | .(1265) OG5459> .(1545) OG11232> .(585). |
Rhodobacter capsulatus | .(1009) OG11232> .(1987) <OG5459 .(328). |
Rhodobacter sphaeroides ATCC.. | .(2210) OG5459> .(773). |
Rhodovulum sulfidophilum | .(3258) OG5459> .(498). |
Roseicitreum antarcticum | .(1851) OG5459> .(1357) |
Roseobacter denitrificans | .(9)... <OG5459 .(2948) OG6198> .(845). |
Roseobacter litoralis Och 149 | .(1142) <OG5459 .(3014) |
Roseobacter ponti | .(943). <OG5459 .(2717) |
Roseibacterium elongatum DSM.. | .(1715) <OG5459 .(1462) |
Roseovarius indicus | .(232). <OG5459 .(587). OG6198> .(4376) |
Ruegeria pomeroyi DSS-3 | .(3127) OG5459> .(765). | .(172). <OG6198 .(281). |
Silicimonas algicola | .(797). <OG5459 .(3439) |
Sulfitobacter pseudonitzschiae | .(2849) OG5459> .(907). |
Tabrizicola piscis | .(1170) OG5459> .(2796) |
Thalassobius gelatinovorus | .(3341) <OG5459 .(349). |
Thioclava nitratireducens | .(148). <OG5459 .(2534) OG11232> .(984). |
Paremcibacter congregatus | .(3095) OG5459> .(518). |
Glycocaulis alkaliphilus | .(2265) OG5459> .(549). |
Hirschia baltica ATCC 49814 | .(2774) OG5459> .(328). |
Hyphomonas neptunium ATCC 15.. | .(3143) <OG5459 .(330). |
Kordiimonas pumila | .(3023) OG5459> .(511). |
Magnetococcus marinus MC-1 |
Micavibrio aeruginosavorus A.. | .(606). <OG5459 .(1692) |
Phreatobacter cathodiphilus |
Phreatobacter stygius | .(1538) <OG6198 OG11232> .(5288) |
Parvularcula bermudensis HTC.. | .(1581) <OG5459 .(1055) |
Achromobacter denitrificans | .(3024) OG5459> .(2234) <OG6198 OG11232> .(889). |
Achromobacter insolitus | .(692). <OG6198 OG11232> .(3646) OG5459> .(1514) |
Achromobacter spanius | .(745). OG5459> .(2151) <OG6198 OG11232> .(2751) |
Achromobacter xylosoxidans | .(789). <OG6198 OG11232> .(3996) OG5459> .(866). |
Advenella kashmirensis WT001 | .(1337) <OG6198 .(1886) |
Advenella mimigardefordensis.. | .(2388) OG6198> .(286). OG5459> .(1567) |
Alcaligenes aquatilis | .(2078) OG5459> .(1323) |
Alcaligenes faecalis | .(1206) OG5459> .(2541) |
Algicoccus marinus | .(1237) <OG6198 .(2247) |
Basilea psittacipulmonis DSM.. | .(61).. OG11232> .(1581) |
Bordetella avium 197N | .(175). <OG6198 .(715). OG5459> .(2416) |
Bordetella bronchialis | .(2928) OG6198> .(1298) OG5459> .(1002) |
Bordetella bronchiseptica | .(723). OG11232> .(2590) OG5459> .(1453) |
Bordetella flabilis | .(3966) OG5459> .(1170) |
Bordetella hinzii | .(2287) OG11232> .(791). <OG6198 .(1233) <OG5459 .(67).. |
Bordetella holmesii | .(1262) <OG5459 .(1414) OG11232> .(557). |
Bordetella parapertussis | .(1209) OG11232> .(2411) OG5459> .(540). |
Bordetella pertussis 18323 | .(1042) <OG5459 .(660). OG6198> .(1792) |
Bordetella petrii | .(735). OG6198> .(2725) OG5459> .(1447) |
Bordetella pseudohinzii | .(1336) <OG5459 .(2198) <OG6198 OG11232> .(564). |
Castellaniella defragrans 65.. | .(964). <OG6198 .(1643) OG11232> .(41).. OG5459> .(844). |
Kerstersia gyiorum | .(2247) OG5459> .(1078) |
Orrella dioscoreae | .(973). <OG6198 .(2)... OG11232> .(208). <OG5459 .(3179) |
Pigmentiphaga aceris | .(1276) <OG5459 .(325). <OG6198 .(3618) |
Taylorella asinigenitalis MCE3 | .(22).. OG11232> .(1464) |
Taylorella equigenitalis | .(21).. OG11232> .(1441) |
Acidovorax carolinensis | .(639). OG5459> .(2971) |
Acidovorax citrulli AAC00-1 | .(191). <OG6198 .(732). OG5459> .(3810) |
Acidovorax monticola | .(412). <OG6198 .(547). OG6198> .(2790) |
Alicycliphilus denitrificans | .(869). OG5459> .(989). <OG6198 .(2419) |
Comamonas kerstersii | .(728). OG5459> .(2462) |
Comamonas koreensis | .(397). OG6198> .(951). OG5459> .(3273) |
Comamonas piscis | .(340). OG6198> .(935). OG5459> .(3299) |
Comamonas serinivorans | .(2379) <OG6198 .(1046) <OG5459 .(344). |
Delftia lacustris | .(4440) <OG6198 .(859). OG5459> .(925). |
Delftia tsuruhatensis | .(4538) <OG6198 .(766). OG5459> .(1186) |
Diaphorobacter aerolatus | .(1998) OG6198> .(1624) |
Diaphorobacter polyhydroxybu.. | .(2229) <OG5459 .(1442) |
Diaphorobacter ruginosibacter | .(705). OG5459> .(3772) <OG6198 .(6)... |
Hydrogenophaga crassostreae | .(609). OG5459> .(3227) <OG6198 .(658). |
Hydrogenophaga pseudoflava | .(3221) OG6198> .(718). <OG5459 .(637). |
Ottowia oryzae | .(1392) OG5459> .(2068) |
Polaromonas naphthalenivoran.. | .(637). OG5459> .(1885) OG11232> .(388). OG6198> .(1195) |
Polaromonas vacuolata | .(1610) <OG6198 OG11232> .(1196) <OG5459 .(554). |
Pulveribacter suum | .(1731) <OG6198 .(1079) <OG5459 .(202). |
Ramlibacter tataouinensis TT.. | .(1551) <OG6198 .(2046) <OG5459 .(309). |
Rhodoferax antarcticus | .(2888) <OG5459 .(683). |
Rhodoferax ferrireducens T118 | .(1117) OG5459> .(776). <OG6198 .(2332) |
Rhodoferax koreense | .(2930) <OG6198 OG11232> .(1159) <OG5459 .(1190) |
Rhodoferax saidenbachensis | .(1539) <OG6198 .(536). <OG1765 <OG2439 .(1099) <OG5459 .(792). |
Rhodoferax sediminis | .(561). OG5459> .(883). <OG6198 .(2637) |
Schlegelella thermodepolymer.. | .(709). OG5459> .(517). <OG6198 .(2339) |
Serpentinomonas mccroryi | .(440). <OG6198 .(547). OG5459> .(1341) |
Serpentinomonas raichei | .(927). <OG5459 .(583). OG6198> .(824). |
Simplicispira suum | .(831). <OG5459 .(2647) |
Variovorax paradoxus S110 | .(167). <OG6198 .(916). OG5459> .(4188) |
Verminephrobacter eiseniae E.. | .(1755) OG5459> .(3150) |
Aquabacterium olei | .(655). OG5459> .(1154) <OG1765 .(1)... <OG2439 .(770). OG6198> .(802). |

Inhella inkyongensis .(2487) OG6198> .(577). <OG5459 .(670).
Leptothrix cholodnii SP-6 .(3354) <OG5459 .(986).
Methylibium petroleiphilum PM1 .(2883) OG6198> .(203). <OG5459 .(736).
Rhizobacter gummiphilus .(1187) OG5459> .(1743) <OG6198 .(2906)
Roseateles depolymerans .(979). OG5459> .(1094) OG6198> .(591). OG2439> .(2153)
Sphaerotilus natans subsp. s.. .(2609) <OG5459 .(870).
Thiomonas arsenitoxydans .(137). OG5459> .(3307)
Thiomonas intermedia .(1312) OG6198> .(459). OG5459> .(1194)
Xylophilus rhododendri .(1884) <OG5459 .(2786) <OG6198 .(555).
Burkholderia cepacia .(857). <OG6198 OG11232> .(19).. <OG5459 .(2437)
Burkholderia cenocepacia .(820). OG11232> .(17).. <OG5459 .(2425)
Burkholderia dolosa AU0158 .(742). <OG6198 OG11232> .(16).. <OG5459 .(2327)
Burkholderia metallica .(770). OG11232> .(17).. <OG5459 .(2388)
Burkholderia multivorans ATC.. .(728). OG11232> .(18).. <OG5459 .(2389)
Burkholderia pyrrocinia .(2854) OG11232> .(17).. <OG5459 .(318).
Burkholderia seminalis .(791). OG11232> .(17).. <OG5459 .(2320)
Burkholderia stagnalis .(862). OG11232> .(18).. <OG5459 .(2429)
Burkholderia stabilis .(3217) OG11232> .(19).. <OG5459 .(168).
Burkholderia ubonensis .(850). OG11232> .(19).. <OG5459 .(2729)
Burkholderia glumae .(1169) OG5459> .(1919)
Burkholderia mallei .(1127) <OG6198 OG11232> .(241). <OG5459 .(1616)
Burkholderia oklahomensis C6.. .(3100) <OG6198 OG11232> .(19).. <OG5459 .(484).
Burkholderia pseudomallei .(2920) <OG6198 OG11232> .(20).. <OG5459 .(482).
Burkholderia thailandensis E.. .(943). <OG6198 OG11232> .(17).. <OG5459 .(2311)
Burkholderia plantarii .(2797) OG5459> .(768).
Caballeronia insecticola .(1183) <OG1765 .(1)... <OG2439 .(696). OG5459> .(848).
Chitinimonas arctica .(477). OG6198> .(1563) <OG5459 .(427). OG1765> .(2152)
Cupriavidus basilensis .(1733) <OG5459 .(1631) <OG6198 OG11232> .(668).
Cupriavidus gilardii .(1668) OG5459> .(440). <OG6198 .(927).
Cupriavidus malaysiensis .(963). OG6198> .(1950) OG5459> .(1014) .(913). <OG6198 OG11232> .(1978)
Cupriavidus nantongensis .(822). OG6198> .(404). <OG5459 .(2235) <OG6198 OG11232> .(810).
Cupriavidus neocaledonicus .(1327) <OG5459 .(1398) <OG6198 OG11232> .(529).
Cupriavidus necator H16 .(1008) OG6198> .(350). <OG5459 .(1705) OG11232> .(569).
Cupriavidus oxalaticus .(1404) <OG5459 .(1465) <OG6198 OG11232> .(532).
Cupriavidus taiwanensis LMG .. .(1294) <OG5459 .(1281) <OG6198 OG11232> .(523).
Ephemeroptericola cinctioste.. .(1176) <OG5459 .(1241)
Lautropia mirabilis .(184). OG11232> .(2)... OG11232> .(149). OG5459> .(2185)
Mycoavidus cysteinexigens .(491). <OG5459 .(1770)
Mycetohabitans rhizoxinica H.. .(1732) OG5459> .(513).
Pandoraea apista .(1994) <OG1765 .(1)... <OG2439 .(559). OG5459> .(978). OG6198> .(1282)
Pandoraea faecigallinarum .(1146) <OG6198 .(896). <OG1765 .(1)... <OG2439 .(581). OG5459> .(1853)
Pandoraea fibrosis .(1842) <OG6198 .(911). <OG1765 .(1)... <OG2439 .(588). OG5459> .(1462)
Pandoraea norimbergensis .(1170) <OG6198 .(1209) <OG5459 .(87).. <OG6198 .(844). OG2439> .(1)... OG1765> .(1980)
Pandoraea oxalativorans .(1153) <OG6198 .(920). <OG1765 .(1)... <OG2439 .(700). OG5459> .(1952)
Pandoraea pnomenusa .(1198) <OG6198 .(860). <OG1765 .(1)... <OG2439 .(579). OG5459> .(2048)
Pandoraea pulmonicola .(1200) <OG6198 .(972). <OG1765 .(1)... <OG2439 .(626). OG5459> .(2128)
Pandoraea sputorum .(1989) <OG1765 .(1)... <OG2439 .(693). OG5459> .(1060) OG6198> .(1191)
Pandoraea thiooxydans .(2470) OG5459> .(1579)
Pandoraea vervacti .(1158) <OG6198 .(875). <OG1765 .(1)... <OG2439 .(663). OG5459> .(2017)
Paraburkholderia aromaticivo.. .(121). <OG5459 .(484). <OG1765 .(1)... <OG2439 .(3220)
Paraburkholderia caffeinilyt.. .(2755) <OG5459 .(471). <OG1765 .(1)... <OG2439 .(712).
Paraburkholderia caribensis .(2772) <OG5459 .(356). <OG1765 .(2)... <OG2439 .(4)...
Paraburkholderia dokdonella .(757). <OG5459 .(1974)
Paraburkholderia graminis .(969). <OG5459 .(774). OG6198> .(164). OG2439> .(1)... OG1765> .(1580)
Paraburkholderia phymatum ST.. .(238). <OG6198 .(1368) .(974). <OG5459 .(399). <OG1765 .(1)... <OG2439 .(1669)
Paraburkholderia phytofirman.. .(2083) OG6198> .(1057) .(1189) <OG5459 .(199). OG6198> .(236). <OG1765 .(1)... <OG2439 .(2257)
Paraburkholderia sprentiae W.. .(764). OG6198> .(47).. .(1560) <OG5459 .(325). <OG1765 .(1)... <OG2439 .(1298)
Paraburkholderia terricola .(1425) <OG1765 .(1)... <OG2439 .(1188) OG5459> .(911).
Paraburkholderia terrae .(984). <OG5459 .(340). <OG1765 .(1)... <OG2439 .(1797)
Paraburkholderia tropica .(831). <OG5459 .(2028)
Paraburkholderia xenovorans .. .(1084) <OG5459 .(307). OG6198> .(217). <OG1765 .(1)... <OG2439 .(2690)
Paraburkholderia acidophila .(874). OG11232> .(21).. <OG5459 .(2481)
Polynucleobacter acidiphobus .(1068) OG5459> .(825).
Polynucleobacter asymbioticu.. .(1279) OG5459> .(837).
Polynucleobacter difficilis .(1089) OG5459> .(904).
Polynucleobacter duraquae .(1143) OG5459> .(892).
Polynucleobacter necessarius .(376). <OG5459 .(1322)
Polynucleobacter paneuropaeus .(695). <OG5459 .(894).
Polynucleobacter wuianus .(792). <OG5459 .(1464)
Ralstonia insidiosa .(66).. OG11232> .(1485) <OG6198 .(117). .(1761) <OG5459 .(1440) OG2439> .(1)... OG1765> .(491).
Ralstonia mannitolilytica .(842). OG11232> <OG6198 .(452). .(225). OG5459> .(687). OG2439> .(1)... OG1765> .(2256)
Ralstonia pseudosolanacearum .(148). <OG6198 OG11232> .(1433) .(2161) <OG5459 .(677). <OG2439 .(529).
Ralstonia solanacearum .(152). <OG6198 OG11232> .(1385) .(2337) <OG2439 .(671). <OG5459 .(101).
Collimonas arenae .(75).. <OG6198 .(1325) <OG6198 OG11232> .(34).. <OG5459 .(1375) <OG5459 .(1287)
Collimonas fungivorans .(1600) <OG6198 OG11232> .(44).. <OG5459 .(1669) <OG5459 .(518). <OG2439 <OG1765 .(982).
Collimonas pratensis .(1553) <OG6198 OG11232> .(41).. <OG5459 .(1791) <OG5459 .(1569)
Herminiimonas arsenitoxidans .(2356) OG11232> .(1066) <OG5459 .(65)..
Herbaspirillum frisingense .(1042) <OG5459 .(3767)
Herbaspirillum huttiense .(1023) <OG5459 .(3949)
Herbaspirillum robiniae .(2164) OG5459> .(2671)
Herbaspirillum rubrisubalbic.. .(893). <OG5459 .(3595) OG6198> .(283).
Herbaspirillum seropedicae .(1086) <OG5459 .(3680)
Janthinobacterium agaricidam.. .(1185) <OG5459 .(3870)
Janthinobacterium lividum .(3206) OG5459> .(1352) OG5459> .(973).
Janthinobacterium svalbarden.. .(3150) OG5459> .(1363) OG5459> .(932).
Massilia albidiflava .(924). <OG5459 .(674). OG6198> .(458). OG11232> .(3988)
Massilia armeniaca .(962). <OG5459 .(2513) OG6198> .(134). <OG2439 .(1628)
Massilia flava .(1813) OG5459> .(2716) <OG6198 .(1327)
Massilia lutea .(1205) OG6198> .(2520) <OG5459 .(1123) OG11232> .(1246)
Massilia oculi .(3712) OG6198> .(40).. <OG5459 .(1239)
Massilia putida .(2158) OG5459> .(301). <OG6198 .(3496)
Massilia umbonata .(3358) <OG5459 .(1095) OG11232> .(1625)
Massilia violaceinigra .(2941) <OG5459 .(3317)
Oxalobacter formigenes .(2063) OG5459> .(141).
Undibacterium parvum .(505). <OG5459 .(3678)
Sutterella faecalis
Sutterella megalosphaeroides
Alysiella filiformis .(1150) <OG5459 .(1086)
Chitinolyticbacter meiyuanen.. .(3601) OG5459> .(76).. OG5459> .(374).
Conchiformibius steedae .(1167) <OG5459 .(827).
Crenobacter cavernae .(1264) OG5459> .(390). <OG6198 OG11232> .(1364)
Eikenella corrodens .(1154) OG11232> .(920). <OG5459 .(16)..
Eikenella exigua .(664). <OG5459 .(983). OG11232> .(195).
Kingella oralis .(356). OG5459> .(2098)
Neisseria animaloris .(87).. OG5459> .(1942)
Neisseria animalis .(700). OG5459> .(576). OG11232> .(690).
Neisseria bacilliformis .(502). <OG5459 .(1786)
Neisseria brasiliensis .(767). <OG5459 .(1680)
Neisseria canis .(254). OG5459> .(2041)
Neisseria chenwenguii .(1012) <OG5459 .(1175) OG11232> .(91)..
Neisseria cinerea .(58).. <OG5459 .(1632)
Neisseria elongata .(1255) <OG5459 .(932).
Neisseria flavescens .(1374) OG5459> .(649).
Neisseria gonorrhoeae .(532). OG5459> .(1490)
Neisseria lactamica .(1555) OG5459> .(520).
Neisseria meningitidis .(1351) OG5459> .(591).
Neisseria musculi .(2582) <OG5459 .(34)..
Neisseria polysaccharea .(1178) <OG5459 .(673).
Neisseria shayeganii .(1279) <OG5459 .(991).
Neisseria subflava .(1301) OG5459> .(639).
Neisseria wadsworthii .(191). OG5459> .(2076)
Neisseria weaveri .(1439) <OG5459 .(570).
Neisseria zalophi OG5459> .(2152)
Neisseria zoodegmatis .(555). <OG5459 .(1705)
Simonsiella muelleri ATCC 29.. .(254). <OG5459 .(2081)
Snodgrassella alvi wkB2 .(1641) <OG5459 .(566).

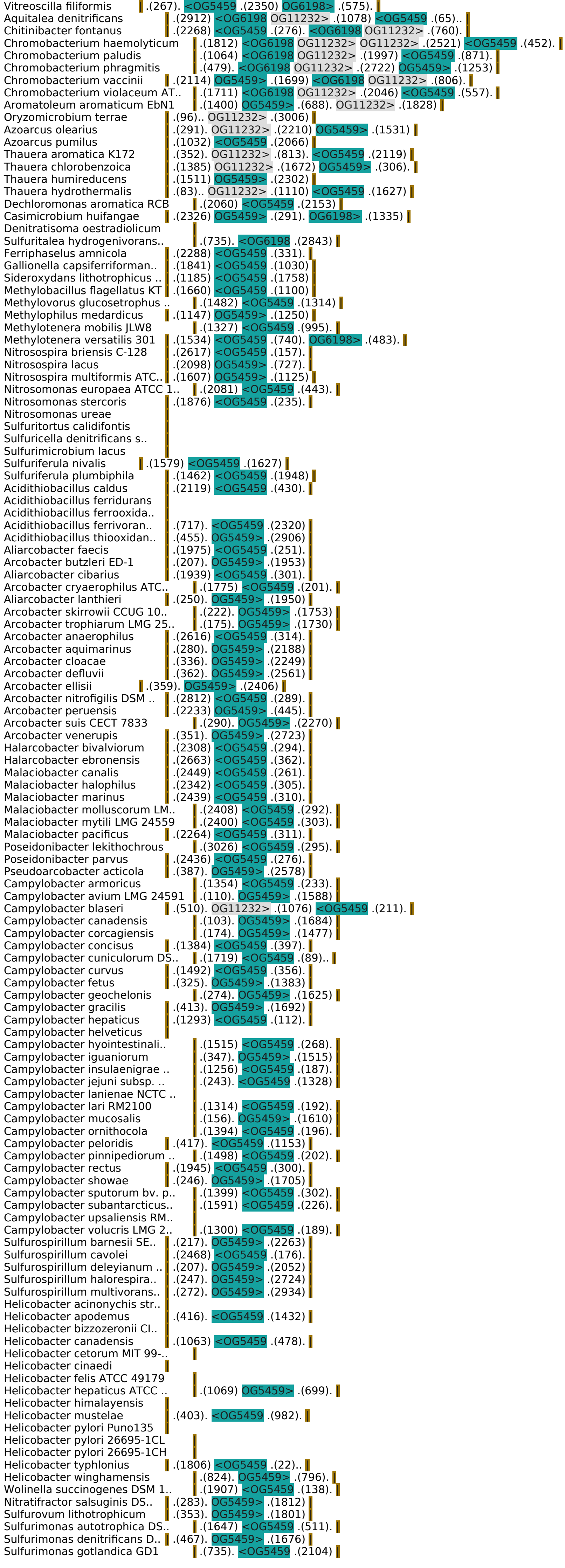

Vitreoscilla filiformis .(267). <OG5459 .(2350) OG6198> .(575).
Aquitalea denitrificans .(2912) <OG6198 OG11232> .(1078) <OG5459 .(65)..
Chitinibacter fontanus .(2268) <OG5459 .(276). <OG6198 OG11232> .(760).
Chromobacterium haemolyticum .(1812) <OG6198 OG11232> OG11232> .(2521) <OG5459 .(452).
Chromobacterium paludis .(1064) <OG6198 OG11232> .(1997) <OG5459 .(871).
Chromobacterium phragmitis .(479). <OG6198 OG11232> .(2722) OG5459> .(1253)
Chromobacterium vaccinii .(2114) OG5459> .(1699) <OG6198 OG11232> .(806).
Chromobacterium violaceum AT.. .(1711) <OG6198 OG11232> .(2046) <OG5459 .(557).
Aromatoleum aromaticum EbN1 .(1400) OG5459> .(688). OG11232> .(1828)
Oryzomicrobium terrae .(96).. OG11232> .(3006)
Azoarcus olearius .(291). OG11232> .(2210) OG5459> .(1531)
Azoarcus pumilus .(1032) <OG5459 .(2066)
Thauera aromatica K172 .(352). OG11232> .(813). <OG5459 .(2119)
Thauera chlorobenzoica .(1385) OG11232> .(1672) OG5459> .(306).
Thauera humireducens .(1511) OG5459> .(2302)
Thauera hydrothermalis .(83).. OG11232> .(1110) <OG5459 .(1627)
Dechloromonas aromatica RCB .(2060) <OG5459 .(2153)
Casimicrobium huifangae .(2326) OG5459> .(291). OG6198> .(1335)
Denitratisoma oestradiolicum
Sulfuritalea hydrogenivorans.. .(735). <OG6198 .(2843)
Ferriphaselus amnicola .(2288) <OG5459 .(331).
Gallionella capsiferriforman.. .(1841) <OG5459 .(1030)
Sideroxydans lithotrophicus .. .(1185) <OG5459 .(1758)
Methylobacillus flagellatus KT .(1660) <OG5459 .(1100)
Methylovorus glucosetrophus .. .(1482) <OG5459 .(1314)
Methylophilus medardicus .(1147) OG5459> .(1250)
Methylotenera mobilis JLW8 .(1327) <OG5459 .(995).
Methylotenera versatilis 301 .(1534) <OG5459 .(740). OG6198> .(483).
Nitrosospira briensis C-128 .(2617) <OG5459 .(157).
Nitrosospira lacus .(2098) OG5459> .(727).
Nitrosospira multiformis ATC.. .(1607) OG5459> .(1125)
Nitrosomonas europaea ATCC 1.. .(2081) <OG5459 .(443).
Nitrosomonas stercoris .(1876) <OG5459 .(235).
Nitrosomonas ureae
Sulfuritortus calidifontis
Sulfuricella denitrificans s..
Sulfurimicrobium lacus
Sulfuriferula nivalis .(1579) <OG5459 .(1627)
Sulfuriferula plumbiphila .(1462) <OG5459 .(1948)
Acidithiobacillus caldus .(2119) <OG5459 .(430).
Acidithiobacillus ferridurans
Acidithiobacillus ferrooxida..
Acidithiobacillus ferrivoran.. .(717). <OG5459 .(2320)
Acidithiobacillus thiooxidan.. .(455). OG5459> .(2906)
Aliarcobacter faecis .(1975) <OG5459 .(251).
Arcobacter butzleri ED-1 .(207). OG5459> .(1953)
Aliarcobacter cibarius .(1939) <OG5459 .(301).
Arcobacter cryaerophilus ATC.. .(1775) <OG5459 .(201).
Aliarcobacter lanthieri .(250). OG5459> .(1950)
Arcobacter skirrowii CCUG 10.. .(222). OG5459> .(1753)
Arcobacter trophiarum LMG 25.. .(175). OG5459> .(1730)
Arcobacter anaerophilus .(2616) <OG5459 .(314).
Arcobacter aquimarinus .(280). OG5459> .(2188)
Arcobacter cloacae .(336). OG5459> .(2249)
Arcobacter defluvii .(362). OG5459> .(2561)
Arcobacter ellisii .(359). OG5459> .(2406)
Arcobacter nitrofigilis DSM .. .(2812) <OG5459 .(289).
Arcobacter peruensis .(2233) OG5459> .(445).
Arcobacter suis CECT 7833 .(290). OG5459> .(2270)
Arcobacter venerupis .(351). OG5459> .(2723)
Halarcobacter bivalviorum .(2308) <OG5459 .(294).
Halarcobacter ebronensis .(2663) <OG5459 .(362).
Malaciobacter canalis .(2449) <OG5459 .(261).
Malaciobacter halophilus .(2342) <OG5459 .(305).
Malaciobacter marinus .(2439) <OG5459 .(310).
Malaciobacter molluscorum LM.. .(2408) <OG5459 .(292).
Malaciobacter mytili LMG 24559 .(2400) <OG5459 .(303).
Malaciobacter pacificus .(2264) <OG5459 .(311).
Poseidonibacter lekithochrous .(3026) <OG5459 .(295).
Poseidonibacter parvus .(2436) <OG5459 .(276).
Pseudoarcobacter acticola .(387). OG5459> .(2578)
Campylobacter armoricus .(1354) <OG5459 .(233).
Campylobacter avium LMG 24591 .(110). OG5459> .(1588)
Campylobacter blaseri .(510). OG11232> .(1076) <OG5459 .(211).
Campylobacter canadensis .(103). OG5459> .(1684)
Campylobacter corcagiensis .(174). OG5459> .(1477)
Campylobacter concisus .(1384) <OG5459 .(397).
Campylobacter cuniculorum DS.. .(1719) <OG5459 .(89)..
Campylobacter curvus .(1492) <OG5459 .(356).
Campylobacter fetus .(325). OG5459> .(1383)
Campylobacter geochelonis .(274). OG5459> .(1625)
Campylobacter gracilis .(413). OG5459> .(1692)
Campylobacter hepaticus .(1293) <OG5459 .(112).
Campylobacter helveticus
Campylobacter hyointestinali.. .(1515) <OG5459 .(268).
Campylobacter iguaniorum .(347). OG5459> .(1515)
Campylobacter insulaenigrae .. .(1256) <OG5459 .(187).
Campylobacter jejuni subsp. .. .(243). <OG5459 .(1328)
Campylobacter lanienae NCTC ..
Campylobacter lari RM2100 .(1314) <OG5459 .(192).
Campylobacter mucosalis .(156). OG5459> .(1610)
Campylobacter ornithocola .(1394) <OG5459 .(196).
Campylobacter peloridis .(417). <OG5459 .(1153)
Campylobacter pinnipediorum .. .(1498) <OG5459 .(202).
Campylobacter rectus .(1945) <OG5459 .(300).
Campylobacter showae .(246). OG5459> .(1705)
Campylobacter sputorum bv. p.. .(1399) <OG5459 .(302).
Campylobacter subantarcticus.. .(1591) <OG5459 .(226).
Campylobacter upsaliensis RM..
Campylobacter volucris LMG 2.. .(1300) <OG5459 .(189).
Sulfurospirillum barnesii SE.. .(217). OG5459> .(2263)
Sulfurospirillum cavolei .(2468) <OG5459 .(176).
Sulfurospirillum deleyianum .. .(207). OG5459> .(2052)
Sulfurospirillum halorespira.. .(247). OG5459> .(2724)
Sulfurospirillum multivorans.. .(272). OG5459> .(2934)
Helicobacter acinonychis str..
Helicobacter apodemus .(416). <OG5459 .(1432)
Helicobacter bizzozeronii CI..
Helicobacter canadensis .(1063) <OG5459 .(478).
Helicobacter cetorum MIT 99-..
Helicobacter cinaedi
Helicobacter felis ATCC 49179
Helicobacter hepaticus ATCC .. .(1069) OG5459> .(699).
Helicobacter himalayensis
Helicobacter mustelae .(403). <OG5459 .(982).
Helicobacter pylori Puno135
Helicobacter pylori 26695-1CL
Helicobacter pylori 26695-1CH
Helicobacter typhlonius .(1806) <OG5459 .(22)..
Helicobacter winghamensis .(824). OG5459> .(796).
Wolinella succinogenes DSM 1.. .(1907) <OG5459 .(138).
Nitratifractor salsuginis DS.. .(283). OG5459> .(1812)
Sulfurovum lithotrophicum .(353). OG5459> .(1801)
Sulfurimonas autotrophica DS.. .(1647) <OG5459 .(511).
Sulfurimonas denitrificans D.. .(467). OG5459> .(1676)
Sulfurimonas gotlandica GD1 .(735). <OG5459 .(2104)

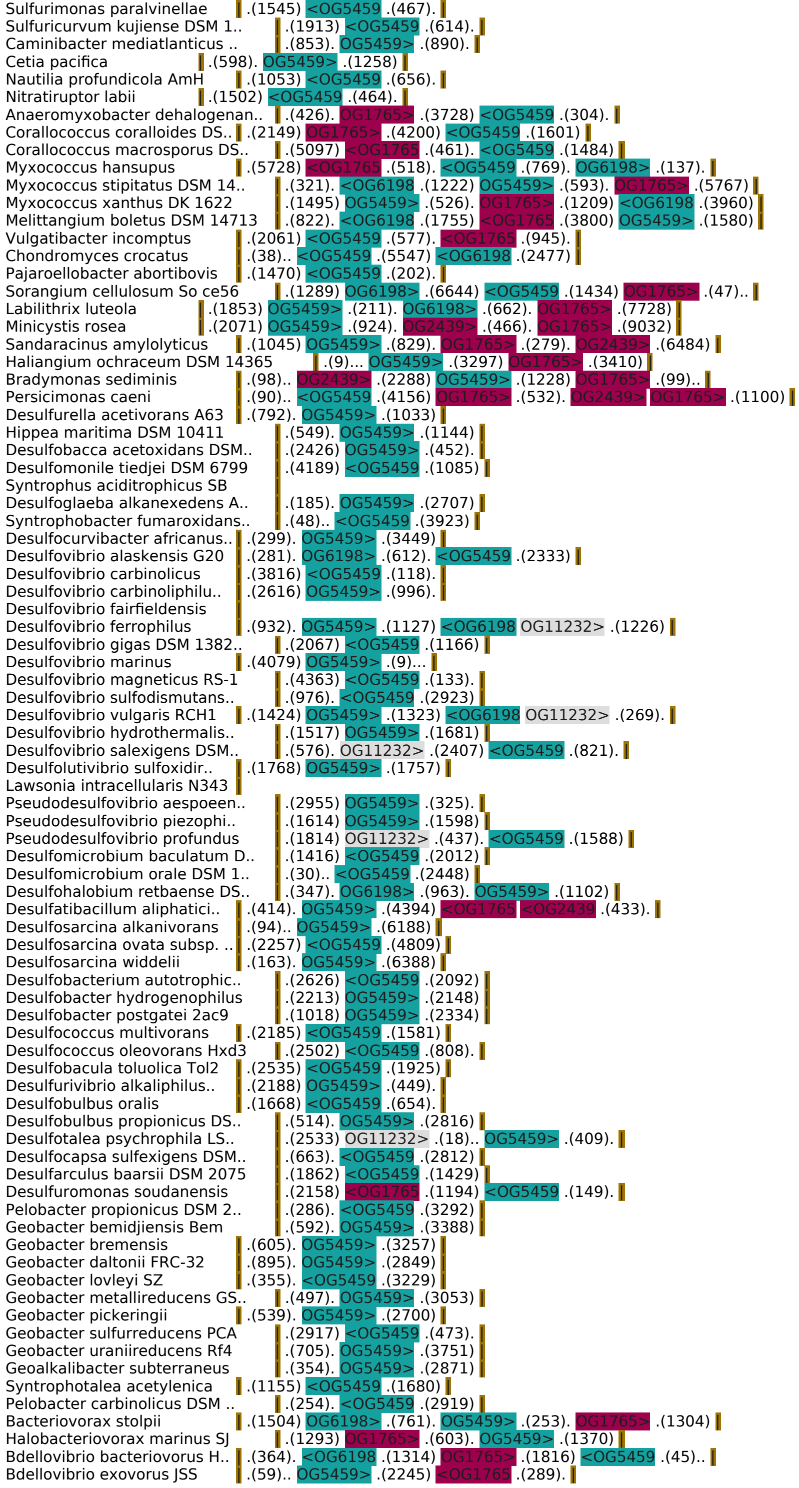

Sulfurimonas paralvinellae .(1545) <OG5459 .(467).
Sulfuricurvum kujiense DSM 1.. .(1913) <OG5459 .(614).
Caminibacter mediatlanticus .. .(853). OG5459> .(890).
Cetia pacifica .(598). OG5459> .(1258)
Nautilia profundicola AmH .(1053) <OG5459 .(656).
Nitratiruptor labii .(1502) <OG5459 .(464).
Anaeromyxobacter dehalogenan.. .(426). OG1765> .(3728) <OG5459 .(304).
Corallococcus coralloides DS.. .(2149) OG1765> .(4200) <OG5459 .(1601)
Corallococcus macrosporus DS.. .(5097) <OG1765 .(461). <OG5459 .(1484)
Myxococcus hansupus .(5728) <OG1765 .(518). <OG5459 .(769). OG6198> .(137).
Myxococcus stipitatus DSM 14.. .(321). <OG6198 .(1222) OG5459> .(593). OG1765> .(5767)
Myxococcus xanthus DK 1622 .(1495) OG5459> .(526). OG1765> .(1209) <OG6198 .(3960)
Melittangium boletus DSM 14713 .(822). <OG6198 .(1755) <OG1765 .(3800) OG5459> .(1580)
Vulgatibacter incomptus .(2061) <OG5459 .(577). <OG1765 .(945).
Chondromyces crocatus .(38).. <OG5459 .(5547) <OG6198 .(2477)
Pajaroellobacter abortibovis .(1470) <OG5459 .(202).
Sorangium cellulosum So ce56 .(1289) OG6198> .(6644) <OG5459 .(1434) OG1765> .(47)..
Labilithrix luteola .(1853) OG5459> .(211). OG6198> .(662). OG1765> .(7728)
Minicystis rosea .(2071) OG5459> .(924). OG2439> .(466). OG1765> .(9032)
Sandaracinus amylolyticus .(1045) OG5459> .(829). OG1765> .(279). OG2439> .(6484)
Haliangium ochraceum DSM 14365 .(9)... OG5459> .(3297) OG1765> .(3410)
Bradymonas sediminis .(98).. OG2439> .(2288) OG5459> .(1228) OG1765> .(99)..
Persicimonas caeni .(90).. <OG5459 .(4156) OG1765> .(532). OG2439> OG1765> .(1100)
Desulfurella acetivorans A63 .(792). OG5459> .(1033)
Hippea maritima DSM 10411 .(549). OG5459> .(1144)
Desulfobacca acetoxidans DSM.. .(2426) OG5459> .(452).
Desulfomonile tiedjei DSM 6799 .(4189) <OG5459 .(1085)
Syntrophus aciditrophicus SB
Desulfoglaeba alkanexedens A.. .(185). OG5459> .(2707)
Syntrophobacter fumaroxidans.. .(48).. <OG5459 .(3923)
Desulfocurvibacter africanus.. .(299). OG5459> .(3449)
Desulfovibrio alaskensis G20 .(281). OG6198> .(612). <OG5459 .(2333)
Desulfovibrio carbinolicus .(3816) <OG5459 .(118).
Desulfovibrio carbinoliphilu.. .(2616) OG5459> .(996).
Desulfovibrio fairfieldensis
Desulfovibrio ferrophilus .(932). OG5459> .(1127) <OG6198 OG11232> .(1226)
Desulfovibrio gigas DSM 1382.. .(2067) <OG5459 .(1166)
Desulfovibrio marinus .(4079) OG5459> .(9)...
Desulfovibrio magneticus RS-1 .(4363) <OG5459 .(133).
Desulfovibrio sulfodismutans.. .(976). <OG5459 .(2923)
Desulfovibrio vulgaris RCH1 .(1424) OG5459> .(1323) <OG6198 OG11232> .(269).
Desulfovibrio hydrothermalis.. .(1517) OG5459> .(1681)
Desulfovibrio salexigens DSM.. .(576). OG11232> .(2407) <OG5459 .(821).
Desulfolutivibrio sulfoxidir.. .(1768) OG5459> .(1757)
Lawsonia intracellularis N343
Pseudodesulfovibrio aespoeen.. .(2955) OG5459> .(325).
Pseudodesulfovibrio piezophi.. .(1614) OG5459> .(1598)
Pseudodesulfovibrio profundus .(1814) OG11232> .(437). <OG5459 .(1588)
Desulfomicrobium baculatum D.. .(1416) <OG5459 .(2012)
Desulfomicrobium orale DSM 1.. .(30).. <OG5459 .(2448)
Desulfohalobium retbaense DS.. .(347). OG6198> .(963). OG5459> .(1102)
Desulfatibacillum aliphatici.. .(414). OG5459> .(4394) <OG1765 <OG2439 .(433).
Desulfosarcina alkanivorans .(94).. OG5459> .(6188)
Desulfosarcina ovata subsp. .. .(2257) <OG5459 .(4809)
Desulfosarcina widdelii .(163). OG5459> .(6388)
Desulfobacterium autotrophic.. .(2626) <OG5459 .(2092)
Desulfobacter hydrogenophilus .(2213) OG5459> .(2148)
Desulfobacter postgatei 2ac9 .(1018) OG5459> .(2334)
Desulfococcus multivorans .(2185) <OG5459 .(1581)
Desulfococcus oleovorans Hxd3 .(2502) <OG5459 .(808).
Desulfobacula toluolica Tol2 .(2535) <OG5459 .(1925)
Desulfurivibrio alkaliphilus.. .(2188) OG5459> .(449).
Desulfobulbus oralis .(1668) <OG5459 .(654).
Desulfobulbus propionicus DS.. .(514). OG5459> .(2816)
Desulfotalea psychrophila LS.. .(2533) OG11232> .(18).. OG5459> .(409).
Desulfocapsa sulfexigens DSM.. .(663). <OG5459 .(2812)
Desulfarculus baarsii DSM 2075 .(1862) <OG5459 .(1429)
Desulfuromonas soudanensis .(2158) <OG1765 .(1194) <OG5459 .(149).
Pelobacter propionicus DSM 2.. .(286). <OG5459 .(3292)
Geobacter bemidjiensis Bem .(592). OG5459> .(3388)
Geobacter bremensis .(605). OG5459> .(3257)
Geobacter daltonii FRC-32 .(895). OG5459> .(2849)
Geobacter lovleyi SZ .(355). <OG5459 .(3229)
Geobacter metallireducens GS.. .(497). OG5459> .(3053)
Geobacter pickeringii .(539). OG5459> .(2700)
Geobacter sulfurreducens PCA .(2917) <OG5459 .(473).
Geobacter uraniireducens Rf4 .(705). OG5459> .(3751)
Geoalkalibacter subterraneus .(354). OG5459> .(2871)
Syntrophotalea acetylenica .(1155) <OG5459 .(1680)
Pelobacter carbinolicus DSM .. .(254). <OG5459 .(2919)
Bacteriovorax stolpii .(1504) OG6198> .(761). OG5459> .(253). OG1765> .(1304)
Halobacteriovorax marinus SJ .(1293) OG1765> .(603). OG5459> .(1370)
Bdellovibrio bacteriovorus H.. .(364). <OG6198 .(1314) OG1765> .(1816) <OG5459 .(45)..
Bdellovibrio exovorus JSS .(59).. OG5459> .(2245) <OG1765 .(289).

Supplementary data 1: The microsynteny analysis of AceI and its orthologs. The colored boxes represent the five genes (OG5459: WP_0014985.1, transaldolase; OG6198: WP_001102208.1, LysR family transcriptional regulator; OG11232: WP_002010078.1, AceI; OG1765: WP_000047938.1, acetyl-CoA C-acyltransferase; OG2439: WP_000132122.1, 3-oxoacyl-ACP reductase), and focal gene AceI is shown in white. Vertical bars represent config/scaffold borders, and numbers in parenthesis give the number of intervening genes between two detected orthologs.

Acinetobacter baumannii	.(3060) OG12704> OG2210> <OG12914 OG6513> <OG3026 .(580).
Acinetobacter calcoaceticus	.(2198) OG12704> OG2210> .(1)... OG6513> .(1575)
Acinetobacter lactucae	.(2049) OG12704> OG2210> <OG12914 OG6513> .(1524)
Acinetobacter nosocomialis M2	.(376). OG12704> OG2210> <OG12914 OG6513> <OG3026 .(3068)
Acinetobacter pittii PHEA-2	.(2712) OG12704> OG2210> <OG12914 OG6513> .(883).
Acinetobacter seifertii	.(2135) OG12704> OG2210> <OG12914 OG6513> <OG3026 .(1501)
Acinetobacter baylyi ADP1	
Acinetobacter chinensis	
Acinetobacter cumulans	
Acinetobacter defluvii	
Acinetobacter dispersus	.(770). <OG12914 OG6513> .(2821)
Acinetobacter equi	
Acinetobacter guillouiae	.(1458) OG3026> .(930). <OG12914 OG6513> .(1750)
Acinetobacter haemolyticus	.(2963) <OG12914 OG6513> .(77)..
Acinetobacter junii	.(2865) <OG12914 OG6513> .(77)..
Acinetobacter larvae	.(1380) OG3026> .(1749)
Acinetobacter lanii	
Acinetobacter oleivorans DR1	.(2184) OG12704> OG2210> <OG12914 OG6513> .(1626)
Acinetobacter schindleri	
Acinetobacter shaoyimingii	
Acinetobacter wanghuae	.(1527) OG3026> .(974).
Moraxella bovoculi	
Moraxella bovis	
Moraxella catarrhalis BBH18	
Moraxella cuniculi	
Moraxella nonliquefaciens	
Moraxella osloensis	
Moraxella ovis	
Psychrobacter alimentarius	.(1820) <OG12914 .(853).
Psychrobacter arcticus 273-4	
Psychrobacter cryohalolentis..	.(1723) <OG12914 .(745).
Psychrobacter urativorans	.(2086) <OG12914 .(206).
Azotobacter chroococcum	.(128). OG6513> .(149).
Azotobacter salinestris	.(3856) <OG6513 .(532).
Azotobacter vinelandii DJ	.(1717) <OG6513 .(2978)
Entomomonas moraniae	
Oblitimonas alkaliphila	.(325). <OG12914 .(1785)
Permianibacter aggregans	
Pseudomonas aeruginosa PAO1H2O	.(2665) <OG12914 OG6513> .(2260) <OG2210 .(471).
Pseudomonas citronellolis	.(2563) OG6513> .(3135) <OG2210 .(381).
Pseudomonas furukawaii	.(2758) OG6513> .(1339) OG2210> .(1476)
Pseudomonas pseudoalcaligene..	
Pseudomonas mendocina S5.2	.(1435) OG6513> .(3221)
Pseudomonas multiresinivorans	.(1026) OG2210> .(3931) <OG2210 .(907).
Pseudomonas agarici	.(2516) <OG6513 .(429). <OG2210 .(1772)
Pseudomonas alkylphenolica	.(1925) <OG2210 .(1304) <OG12914 OG6513> .(1740)
Pseudomonas amygdali pv. tab..	.(462). OG2210> .(4789)
Pseudomonas cichorii JBC1	
Pseudomonas fuscovaginae	.(49).. <OG2210 .(5625)
Pseudomonas syringae pv. tom..	.(4815) <OG2210 .(622).
Pseudomonas viridiflava	.(1371) <OG6513 .(1260) OG2210> .(2650)
Pseudomonas antarctica	.(327). <OG2210 .(5244)
Pseudomonas arsenicoxydans	.(573). <OG2210 .(5198)
Pseudomonas asplenii	.(1060) <OG2210 .(3123) OG6513> .(1396)
Pseudomonas azotoformans	.(4506) OG2210> .(1411)
Pseudomonas brenneri	.(724). <OG12914 .(4016) OG2210> .(613).
Pseudomonas cedrina	.(5385) OG2210> .(400).
Pseudomonas corrugata	.(1228) <OG12914 .(2117) OG2210> .(1894)
Pseudomonas extremorientalis	.(3674) OG2210> .(1932)
Pseudomonas fluorescens	.(2348) OG2210> .(2377) <OG6513 .(1074)
Pseudomonas mandelii JR-1	.(3056) <OG2210 .(1781) <OG12914 OG6513> .(1183)
Pseudomonas mediterranea	.(3163) <OG12914 .(2134) OG2210> .(153).
Pseudomonas mucidolens	
Pseudomonas orientalis	.(322). OG2210> .(4805)
Pseudomonas protegens CHA0	.(1507) <OG12914 OG6513> .(4220) <OG2210 .(409).
Pseudomonas rhodesiae	.(523). OG2210> .(4600)
Pseudomonas synxantha	.(321). OG2210> .(5496)
Pseudomonas trivialis	.(1596) OG2210> .(1921) <OG6513 .(1372)
Pseudomonas veronii	.(350). OG2210> .(1860) OG6513> .(3847)
Pseudomonas balearica DSM 6083	.(2574) OG6513> .(1411)
Pseudomonas stutzeri	.(2290) OG6513> .(1840)
Pseudomonas xanthomarina	.(3212) OG6513> .(661).
Pseudomonas brassicacearum	.(397). OG2210> .(3454) <OG12914 .(2030)
Pseudomonas chlororaphis	.(1521) <OG12914 OG6513> .(4078) <OG2210 .(377).
Pseudomonas lundensis	.(2012) OG2210> .(848). OG6513> .(1483)
Pseudomonas entomophila L48	.(2329) <OG2210 .(1070) <OG12914 OG6513> .(1664)
Pseudomonas extremaustralis	.(4012) <OG2210 .(1944)
Pseudomonas fulva	.(2748) <OG12914 OG6513> .(1509)
Pseudomonas monteilii	.(1464) OG2210> .(2013) <OG12914 OG6513> .(1827)
Pseudomonas mosselii	.(1971) <OG2210 .(1265) <OG12914 OG6513> .(1814)
Pseudomonas plecoglossicida	.(277). <OG2210 .(967). <OG12914 OG6513> .(3573)
Pseudomonas putida NBRC 14164	.(3476) <OG12914 OG6513> .(1890)
Pseudomonas glycinae	.(752). <OG2210 .(4510) <OG12914 OG6513> .(406).
Pseudomonas granadensis	.(1615) <OG12914 OG6513> .(3457) <OG2210 .(52)..
Pseudomonas guangdongensis	
Pseudomonas knackmussii	.(2864) <OG6513 .(2313) <OG2210 .(386).
Pseudomonas koreensis	.(1944) <OG2210 .(1704) <OG12914 OG6513> .(1789)
Pseudomonas kribbensis	.(1381) <OG12914 OG6513> .(3850) <OG2210 .(404).
Pseudomonas lactis	.(375). OG2210> .(4989)
Pseudomonas lalkuanensis	.(2511) <OG6513 .(2916)
Pseudomonas lini	.(591). <OG2210 .(2353) OG2210> .(2756)
Pseudomonas litoralis	.(1204) <OG6513 .(2472)
Pseudomonas lurida	.(347). OG2210> .(5059)
Pseudomonas marincola	
Pseudomonas oryzae	
Pseudomonas otitidis	.(2944) OG6513> .(1460) <OG2210 .(1110)
Pseudomonas pohangensis	.(1798) OG6513> .(1644)
Pseudomonas prosekii	.(2082) <OG2210 .(1865) <OG12914 OG6513> .(1353)
Pseudomonas psychrophila	.(3339) <OG2210 .(409). OG6513> .(909).
Pseudomonas reinekei	.(2104) <OG2210 .(3438)
Pseudomonas rhizosphaerae	.(1060) <OG6513 .(3033)
Pseudomonas salegens	.(1548) OG6513> .(1861)
Pseudomonas sabulinigri	
Pseudomonas sediminis	
Pseudomonas sihuiensis	
Pseudomonas silesiensis	.(245). OG2210> .(3549) <OG2210 .(2141)
Pseudomonas simiae	.(332). OG2210> .(5171)
Pseudomonas soli	.(2443) OG2210> .(275). <OG12914 OG6513> .(2906)
Pseudomonas thivervalensis	.(2260) OG2210> .(3281) <OG12914 .(102).
Pseudomonas umsongensis	.(402). OG2210> .(5500)
Pseudomonas vancouverensis	.(1132) OG2210> .(829). <OG12914 OG6513> .(3798)
Pseudomonas versuta	.(2507) OG6513> .(1111) <OG2210 .(847).
Pseudomonas xinjiangensis	.(1626) <OG12914 .(582). OG6513> .(1026)
Pseudomonas yamanorum	.(2816) OG6513> .(2715) <OG2210 .(599).
Acidihalobacter aeolianus	.(2769) <OG2210 .(374).
Acidihalobacter ferrooxydans	
Alkalilimnicola ehrlichii ML..	
Aquisalimonas sp. 2447	
Halorhodospira halochloris	.(2026) OG6513> .(520).
Halorhodospira halophila SL1	
Spiribacter curvatus	
Spiribacter roseus	
Spiribacter salinus M19-40	
Thioalkalivibrio nitratiredu..	.(1051) OG6513> .(2393)
Thioalkalivibrio paradoxus A..	.(2041) <OG6513 .(1281)
Thioalkalivibrio sulfidiphil..	.(683). <OG6513 .(2582)

Thioalkalivibrio versutus .(1707) <OG6513 .(994).
Allochromatium vinosum DSM 180 .(2912) <OG6513 .(137).
Marichromatium purpuratum 984 .(3084) <OG6513 .(147).
Nitrosococcus halophilus Nc 4
Nitrosococcus oceani ATCC 19..
Nitrosococcus watsonii C-113
Nitrosococcus wardiae .(359). <OG3026 .(3311)
Thermochromatium tepidum ATC..
Thioflavicoccus mobilis 8321
Thiocystis violascens DSM 198
Granulosicoccus antarcticus .. .(2157) <OG12914 .(4481)
Sulfuriflexus mobilis
Guyparkeria halophila
Halothiobacillus neapolitanu..
Sulfurivermis fontis
Wenzhouxiangella marina
Woeseia oceani
Actinobacillus delphinicola
Actinobacillus equuli subsp...
Actinobacillus pleuropneumon..
Actinobacillus porcitonsilla..
Actinobacillus suis ATCC 33415
Aggregatibacter actinomycete..
Aggregatibacter aphrophilus ..
Aggregatibacter segnis ATCC ..
Avibacterium volantium
Basfia succiniciproducens
Bibersteinia trehalosi USDA-..
Bisgaardia hudsonensis
Frederiksenia canicola
Glaesserella parasuis SH0165
Haemophilus aegyptius
Haemophilus haemolyticus
Haemophilus influenzae
Haemophilus pittmaniae
Histophilus somni
Mannheimia haemolytica USMAR..
Mannheimia ovis
Mannheimia varigena USDA-ARS..
Otariodibacter oris
Pasteurella dagmatis
Pasteurella multocida
Pasteurella skyensis
Rodentibacter heylii
Vespertiliibacter pulmonis
Aeromonas allosaccharophila
Aeromonas encheleia .(617). <OG12914 .(3390)
Aeromonas hydrophila .(784). <OG12914 .(3576)
Aeromonas media
Aeromonas salmonicida
Aeromonas simiae .(960). <OG12914 .(2592)
Aeromonas veronii
Oceanisphaera avium .(1013) <OG12914 .(1501)
Oceanisphaera profunda
Tolumonas auensis DSM 9187
Zobellella denitrificans
Aerosticca soli
Ahniella affigens
Dokdonella koreensis DS-123
Dyella thiooxydans
Frateuria aurantia DSM 6220
Luteibacter pinisoli
Luteibacter rhizovicinus DSM..
Rhodanobacter denitrificans .(2664) <OG6513 .(1171)
Arenimonas daejeonensis
Luteimonas chenhongjianii
Luteimonas granuli
Lysobacter alkalisoli
Lysobacter antibioticus .(631). <OG6513 .(4169)
Lysobacter capsici
Lysobacter enzymogenes .(4247) OG6513> .(587).
Lysobacter gummosus
Lysobacter lycopersici
Lysobacter maris
Lysobacter oculi
Lysobacter soli
Pseudolysobacter antarcticus
Pseudoxanthomonas mexicana
Pseudoxanthomonas spadix BD-..
Stenotrophomonas acidaminiph..
Stenotrophomonas maltophilia
Stenotrophomonas rhizophila
Thermomonas brevis
Xanthomonas albilineans
Xanthomonas cassavae CFBP 4642
Xanthomonas campestris pv. r..
Xanthomonas citri
Xanthomonas cucurbitae
Xanthomonas euroxanthea
Xanthomonas fragariae
Xanthomonas hortorum
Xanthomonas hyacinthi
Xanthomonas oryzae pv. oryzi..
Xanthomonas phaseoli pv. die..
Xanthomonas vesicatoria ATCC..
Xylella fastidiosa Temecula1
Xylella taiwanensis
Agarilytica rhodophyticola
Cellvibrio japonicus Ueda107 .(3512) <OG12914 .(103).
Saccharophagus degradans 2-40 .(3779) OG6513> .(275).
Simiduia agarivorans SA1 = D..
Congregibacter litoralis KT71 .(1527) <OG6513 .(2278)
Halioglobus maricola
Kineobactrum salinum
Microbulbifer aggregans
Microbulbifer agarilyticus
Microbulbifer hydrolyticus
Microbulbifer thermotolerans
Oceanicoccus sagamiensis .(2244) <OG6513 .(1677)
Zhongshania aliphaticivorans
Alcanivorax borkumensis SK2 .(28).. OG6513> .(2721)
Alcanivorax dieselolei B5 .(3588) <OG12914 .(798).
Alcanivorax pacificus W11-5
Ketobacter alkanivorans
Bermanella marisrubri
Marinobacterium aestuarii
Marinomonas arctica
Marinomonas mediterranea MMB-1
Marinomonas posidonica IVIA-..
Marinomonas primoryensis
Neptunomonas concharum
Neptunomonas phycophila .(1263) OG6513> .(2282)
Thalassolituus oleivorans MI.. .(2259) OG6513> .(1254)
Chromohalobacter salexigens ..
Cobetia marina .(2970) <OG12914 .(389).
Halomonas aestuarii .(1196) <OG12914 .(2020)

Halomonas beimenensis	.(3365) <OG12914 .(285).
Halomonas campisalis	.(1171) OG6513> .(2872)
Halomonas chromatireducens	.(1678) OG6513> .(1818)
Halomonas elongata DSM 2581	.(296). <OG2210 .(2862) <OG12914 .(546).
Halomonas huangheensis	.(3477) <OG12914 .(557).
Halomonas hydrothermalis	.(885). OG6513> .(2687)
Halomonas piezotolerans	.(841). OG6513> .(2683)
Halomonas socia	
Halomonas subglaciescola	.(2462) OG6513> .(318).
Halomonas titanicae	.(4454) <OG6513 .(251).
Kushneria konosiri	.(1685) <OG12914 .(241). <OG6513 .(1237)
Kushneria marisflavi	.(2071) <OG12914 .(1142)
Pistricoccus aurantiacus	.(2020) <OG12914 .(1411)
Salinicola tamaricis	.(1338) <OG2210 .(1687)
Zymobacter palmae	
Endozoicomonas montiporae CL..	.(3649) <OG6513 .(1077)
Gynuella sunshinyii YC6258	.(4691) OG6513> .(484).
Reinekea forsetii	
Saccharospirillum mangrovi	.(1371) OG6513> .(2022)
Hahella chejuensis KCTC 2396	.(5856) <OG6513 .(405).
Spartinivicinus ruber	
Kangiella geojedonensis	.(1067) <OG6513 .(1187)
Kangiella koreensis DSM 16069	
Kangiella profundi	
Kangiella sediminilitoris	
Litoricola lipolytica	
Oleiphilus messinensis	
Aliivibrio salmonicida LFI1238	
Grimontia hollisae	
Paraphotobacterium marinum	
Photobacterium gaetbulicola ..	
Salinivibrio kushneri	
Vibrio alfacsensis	
Vibrio antiquarius	
Vibrio diabolicus	
Vibrio azureus	.(41).. OG6513> .(2521)
Vibrio campbellii	
Vibrio harveyi	
Vibrio jasicida 090810c	
Vibrio natriegens NBRC 15636..	.(1366) <OG6513 .(284).
Vibrio owensii	
Vibrio parahaemolyticus	
Vibrio rotiferianus	
Vibrio anguillarum	
Vibrio aphrogenes	
Vibrio aquimaris	
Vibrio astriarenae	
Vibrio atlanticus	.(980). OG6513> .(395).
Vibrio cholerae MS6	
Vibrio cyclitrophicus	.(1013) <OG6513 .(447).
Vibrio europaeus	
Vibrio tubiashii ATCC 19109	
Vibrio fluvialis	
Vibrio furnissii	
Vibrio kanaloae	.(947). <OG6513 .(328).
Vibrio metoecus	
Vibrio metschnikovii	
Vibrio mediterranei	
Vibrio navarrensis	.(1184) <OG6513 .(1476)
Vibrio ponticus	.(75).. OG6513> .(1205)
Vibrio qinghaiensis	
Vibrio rumoiensis	
Vibrio scophthalmi	.(740). <OG6513 .(1977)
Vibrio spartinae	
Vibrio tapetis subsp. tapetis	
Vibrio taketomensis	
Vibrio tritonius	
Vibrio vulnificus	
Allofrancisella frigidaquae	.(53).. <OG12914 .(1437)
Allofrancisella guangzhouensis	
Francisella adeliensis	
Francisella frigiditurris	
Francisella halioticida	.(2111) <OG12914 .(68)..
Francisella hispaniensis	
Francisella marina	
Francisella noatunensis subs..	
Francisella opportunistica	
Francisella orientalis LADL-..	
Francisella orientalis FNO12	
Francisella orientalis FNO24	
Francisella persica ATCC VR-..	
Francisella philomiragia	
Francisella salina	
Francisella uliginis	
Beggiatoa leptomitoformis	
Hydrogenovibrio crunogenus X..	
Hydrogenovibrio marinus	
Hydrogenovibrio thermophilus	
Methylophaga frappieri	
Methylophaga nitratireducent..	
Piscirickettsia salmonis	
Thiomicrospira aerophila AL3	
Thiomicrospira cyclica ALM1	
Thiomicrorhabdus aquaedulcis	
Thiomicrorhabdus indica	
Thiosulfatimonas sediminis	
Thiosulfativibrio zosterae	.(1376) <OG12914 .(979).
Alteromonas addita	.(2273) OG6513> .(1576)
Alteromonas australica	.(2079) <OG2210 .(1582)
Alteromonas mediterranea	.(2064) OG6513> .(1671)
Alteromonas naphthalenivorans	.(1119) <OG6513 .(3059)
Alteromonas pelagimontana	.(3270) <OG6513 .(384).
Alteromonas stellipolaris LM..	.(1132) <OG6513 .(2735)
Catenovulum sediminis	.(2416) <OG6513 .(951).
Glaciecola amylolytica	
Glaciecola nitratireducens F..	
Hydrocarboniclastica marina	.(2483) <OG6513 .(1078)
Marinobacter adhaerens HP15	.(480). <OG6513 .(3535)
Marinobacter fonticola	.(826). <OG6513 .(3108)
Marinobacter hydrocarbonocla..	.(678). <OG6513 .(2903)
Marinobacter psychrophilus	.(2339) OG6513> .(1152)
Marinobacter salarius	.(2913) <OG6513 .(1034)
Marinobacter salinus	.(2298) OG6513> .(1402)
Saliniradius amylolyticus	
Salinimonas lutimaris	.(1478) <OG6513 .(2080)
Salinimonas sediminis	.(2224) OG6513> .(1466)
Colwellia beringensis	.(1025) OG6513> .(2781)
Colwellia psychrerythraea 34H	
Litorilituus sediminis	.(3296) OG6513> .(368).
Thalassotalea crassostreae	
Ferrimonas balearica DSM 9799	
Idiomarina andamanensis	.(1444) <OG6513 .(756).
Idiomarina loihiensis L2TR	.(465). <OG6513 .(2176)
Moritella marina ATCC 15381	
Moritella yayanosii	.(1123) <OG6513 .(608). <OG12914 .(2032)
Parashewanella spongiae	

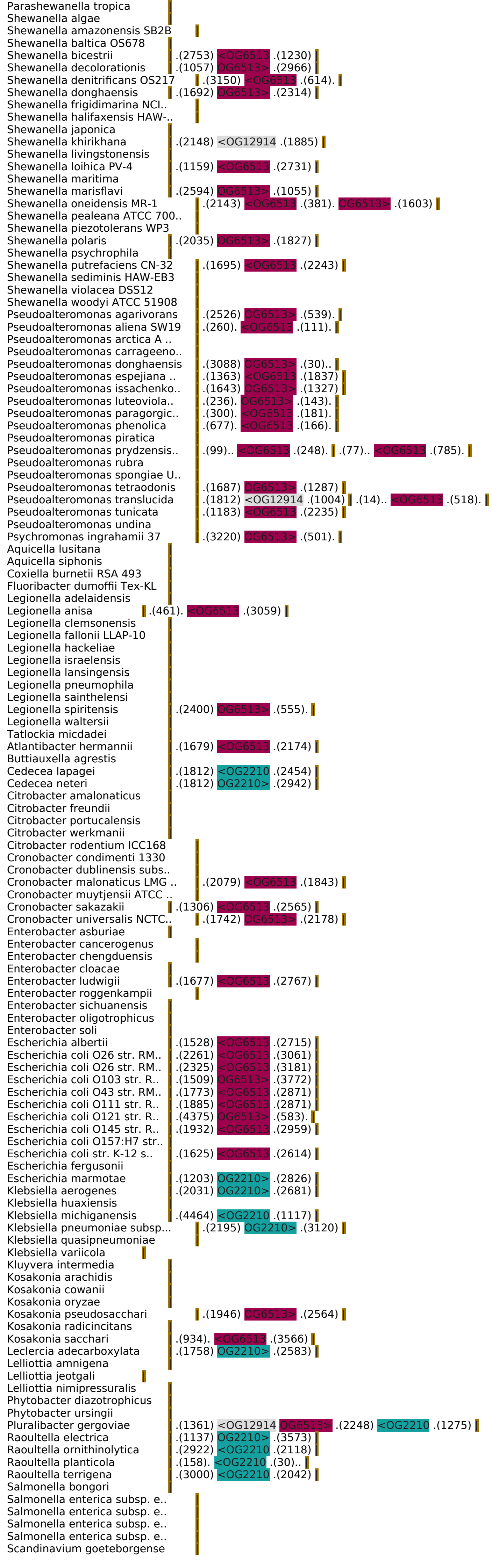
Parashewanella tropica
Shewanella algae
Shewanella amazonensis SB2B
Shewanella baltica OS678
Shewanella bicestrii .(2753) <OG6513 .(1230)
Shewanella decolorationis .(1057) OG6513> .(2966)
Shewanella denitrificans OS217 .(3150) <OG6513 .(614).
Shewanella donghaensis .(1692) OG6513> .(2314)
Shewanella frigidimarina NCI..
Shewanella halifaxensis HAW-..
Shewanella japonica
Shewanella khirikhana .(2148) <OG12914 .(1885)
Shewanella livingstonensis
Shewanella loihica PV-4 .(1159) <OG6513 .(2731)
Shewanella maritima
Shewanella marisflavi .(2594) OG6513> .(1055)
Shewanella oneidensis MR-1 .(2143) <OG6513 .(381). OG6513> .(1603)
Shewanella pealeana ATCC 700..
Shewanella piezotolerans WP3
Shewanella polaris .(2035) OG6513> .(1827)
Shewanella psychrophila
Shewanella putrefaciens CN-32 .(1695) <OG6513 .(2243)
Shewanella sediminis HAW-EB3
Shewanella violacea DSS12
Shewanella woodyi ATCC 51908
Pseudoalteromonas agarivorans .(2526) OG6513> .(539).
Pseudoalteromonas aliena SW19 .(260). <OG6513 .(111).
Pseudoalteromonas arctica A ..
Pseudoalteromonas carrageeno..
Pseudoalteromonas donghaensis .(3088) OG6513> .(30)..
Pseudoalteromonas espejiana .. .(1363) <OG6513 .(1837)
Pseudoalteromonas issachenko.. .(1643) OG6513> .(1327)
Pseudoalteromonas luteoviola.. .(236). OG6513> .(143).
Pseudoalteromonas paragorgic.. .(300). <OG6513 .(181).
Pseudoalteromonas phenolica .(677). <OG6513 .(166).
Pseudoalteromonas piratica
Pseudoalteromonas prydzensis.. .(99).. <OG6513 .(248). .(77).. <OG6513 .(785).
Pseudoalteromonas rubra
Pseudoalteromonas spongiae U..
Pseudoalteromonas tetraodonis .(1687) OG6513> .(1287)
Pseudoalteromonas translucida .(1812) <OG12914 .(1004) .(14).. <OG6513 .(518).
Pseudoalteromonas tunicata .(1183) <OG6513 .(2235)
Pseudoalteromonas undina
Psychromonas ingrahamii 37 .(3220) OG6513> .(501).
Aquicella lusitana
Aquicella siphonis
Coxiella burnetii RSA 493
Fluoribacter dumoffii Tex-KL
Legionella adelaidensis
Legionella anisa .(461). <OG6513 .(3059)
Legionella clemsonensis
Legionella fallonii LLAP-10
Legionella hackeliae
Legionella israelensis
Legionella lansingensis
Legionella pneumophila
Legionella sainthelensi
Legionella spiritensis .(2400) OG6513> .(555).
Legionella waltersii
Tatlockia micdadei
Atlantibacter hermannii .(1679) <OG6513 .(2174)
Buttiauxella agrestis
Cedecea lapagei .(1812) <OG2210 .(2454)
Cedecea neteri .(1812) OG2210> .(2942)
Citrobacter amalonaticus
Citrobacter freundii
Citrobacter portucalensis
Citrobacter werkmanii
Citrobacter rodentium ICC168
Cronobacter condimenti 1330
Cronobacter dublinensis subs..
Cronobacter malonaticus LMG .. .(2079) <OG6513 .(1843)
Cronobacter muytjensii ATCC ..
Cronobacter sakazakii .(1306) <OG6513 .(2565)
Cronobacter universalis NCTC.. .(1742) OG6513> .(2178)
Enterobacter asburiae
Enterobacter cancerogenus
Enterobacter chengduensis
Enterobacter cloacae
Enterobacter ludwigii .(1677) <OG6513 .(2767)
Enterobacter roggenkampii
Enterobacter sichuanensis
Enterobacter oligotrophicus
Enterobacter soli
Escherichia albertii .(1528) <OG6513 .(2715)
Escherichia coli O26 str. RM.. .(2261) <OG6513 .(3061)
Escherichia coli O26 str. RM.. .(2325) <OG6513 .(3181)
Escherichia coli O103 str. R.. .(1509) OG6513> .(3772)
Escherichia coli O43 str. RM.. .(1773) <OG6513 .(2871)
Escherichia coli O111 str. R.. .(1885) <OG6513 .(2871)
Escherichia coli O121 str. R.. .(4375) OG6513> .(583).
Escherichia coli O145 str. R.. .(1932) <OG6513 .(2959)
Escherichia coli O157:H7 str..
Escherichia coli str. K-12 s.. .(1625) <OG6513 .(2614)
Escherichia fergusonii
Escherichia marmotae .(1203) OG2210> .(2826)
Klebsiella aerogenes .(2031) OG2210> .(2681)
Klebsiella huaxiensis
Klebsiella michiganensis .(4464) <OG2210 .(1117)
Klebsiella pneumoniae subsp... .(2195) OG2210> .(3120)
Klebsiella quasipneumoniae
Klebsiella variicola
Kluyvera intermedia
Kosakonia arachidis
Kosakonia cowanii
Kosakonia oryzae
Kosakonia pseudosacchari .(1946) OG6513> .(2564)
Kosakonia radicincitans
Kosakonia sacchari .(934). <OG6513 .(3566)
Leclercia adecarboxylata .(1758) OG2210> .(2583)
Lelliottia amnigena
Lelliottia jeotgali
Lelliottia nimipressuralis
Phytobacter diazotrophicus
Phytobacter ursingii
Pluralibacter gergoviae .(1361) <OG12914 OG6513> .(2248) <OG2210 .(1275)
Raoultella electrica .(1137) OG2210> .(3573)
Raoultella ornithinolytica .(2922) <OG2210 .(2118)
Raoultella planticola .(158). <OG2210 .(30)..
Raoultella terrigena .(3000) <OG2210 .(2042)
Salmonella bongori
Salmonella enterica subsp. e..
Salmonella enterica subsp. e..
Salmonella enterica subsp. e..
Salmonella enterica subsp. e..
Scandinavium goeteborgense

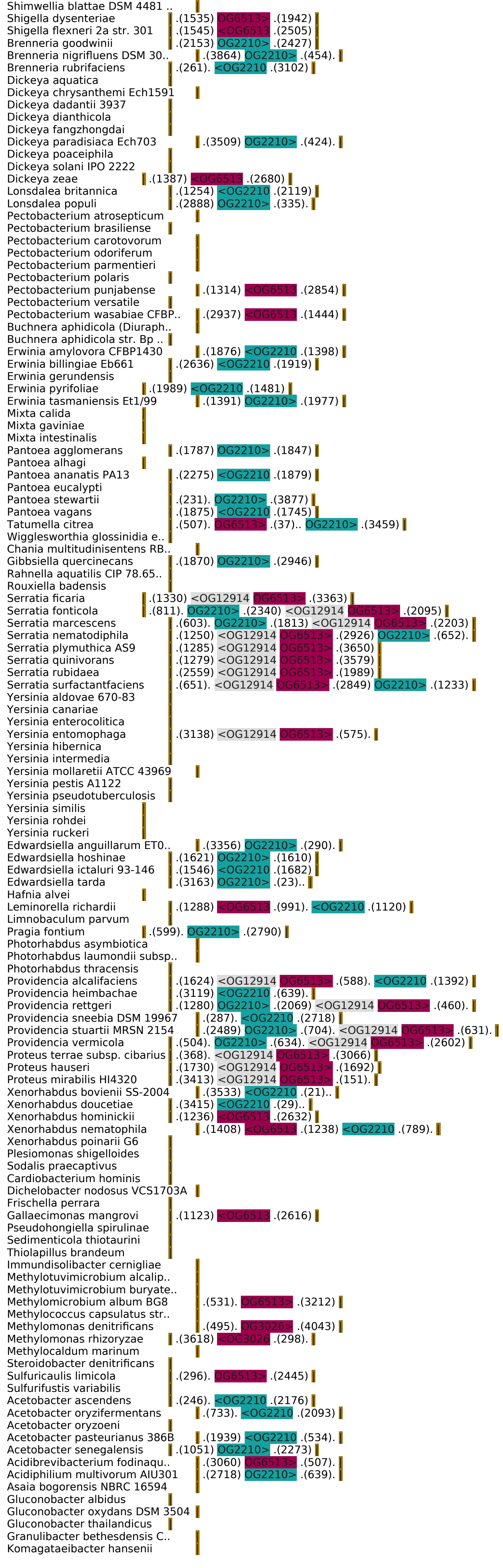
Shimwellia blattae DSM 4481 ..
Shigella dysenteriae .(1535) OG6513> .(1942)
Shigella flexneri 2a str. 301 .(1545) <OG6513 .(2505)
Brenneria goodwinii .(2153) OG2210> .(2427)
Brenneria nigrifluens DSM 30.. .(3864) OG2210> .(454).
Brenneria rubrifaciens .(261). <OG2210 .(3102)
Dickeya aquatica
Dickeya chrysanthemi Ech1591
Dickeya dadantii 3937
Dickeya dianthicola
Dickeya fangzhongdai
Dickeya paradisiaca Ech703 .(3509) OG2210> .(424).
Dickeya poaceiphila
Dickeya solani IPO 2222
Dickeya zeae .(1387) <OG6513 .(2680)
Lonsdalea britannica .(1254) <OG2210 .(2119)
Lonsdalea populi .(2888) OG2210> .(335).
Pectobacterium atrosepticum
Pectobacterium brasiliense
Pectobacterium carotovorum
Pectobacterium odoriferum
Pectobacterium parmentieri
Pectobacterium polaris
Pectobacterium punjabense .(1314) <OG6513 .(2854)
Pectobacterium versatile
Pectobacterium wasabiae CFBP.. .(2937) <OG6513 .(1444)
Buchnera aphidicola (Diuraph..
Buchnera aphidicola str. Bp ..
Erwinia amylovora CFBP1430 .(1876) <OG2210 .(1398)
Erwinia billingiae Eb661 .(2636) <OG2210 .(1919)
Erwinia gerundensis
Erwinia pyrifoliae .(1989) <OG2210 .(1481)
Erwinia tasmaniensis Et1/99 .(1391) OG2210> .(1977)
Mixta calida
Mixta gaviniae
Mixta intestinalis
Pantoea agglomerans .(1787) OG2210> .(1847)
Pantoea alhagi
Pantoea ananatis PA13 .(2275) <OG2210 .(1879)
Pantoea eucalypti
Pantoea stewartii .(231). OG2210> .(3877)
Pantoea vagans .(1875) <OG2210 .(1745)
Tatumella citrea .(507). OG6513> .(37).. OG2210> .(3459)
Wigglesworthia glossinidia e..
Chania multitudinisentens RB..
Gibbsiella quercinecans .(1870) OG2210> .(2946)
Rahnella aquatilis CIP 78.65..
Rouxiella badensis
Serratia ficaria .(1330) <OG12914 OG6513> .(3363)
Serratia fonticola .(811). OG2210> .(2340) <OG12914 OG6513> .(2095)
Serratia marcescens .(603). OG2210> .(1813) <OG12914 OG6513> .(2203)
Serratia nematodiphila .(1250) <OG12914 OG6513> .(2926) OG2210> .(652).
Serratia plymuthica AS9 .(1285) <OG12914 OG6513> .(3650)
Serratia quinivorans .(1279) <OG12914 OG6513> .(3579)
Serratia rubidaea .(2559) <OG12914 OG6513> .(1989)
Serratia surfactantfaciens .(651). <OG12914 OG6513> .(2849) OG2210> .(1233)
Yersinia aldovae 670-83
Yersinia canariae
Yersinia enterocolitica
Yersinia entomophaga .(3138) <OG12914 OG6513> .(575).
Yersinia hibernica
Yersinia intermedia
Yersinia mollaretii ATCC 43969
Yersinia pestis A1122
Yersinia pseudotuberculosis
Yersinia similis
Yersinia rohdei
Yersinia ruckeri
Edwardsiella anguillarum ET0.. .(3356) OG2210> .(290).
Edwardsiella hoshinae .(1621) OG2210> .(1610)
Edwardsiella ictaluri 93-146 .(1546) <OG2210 .(1682)
Edwardsiella tarda .(3163) OG2210> .(23)..
Hafnia alvei
Leminorella richardii .(1288) <OG6513 .(991). <OG2210 .(1120)
Limnobaculum parvum
Pragia fontium .(599). OG2210> .(2790)
Photorhabdus asymbiotica
Photorhabdus laumondii subsp..
Photorhabdus thracensis
Providencia alcalifaciens .(1624) <OG12914 OG6513> .(588). <OG2210 .(1392)
Providencia heimbachae .(3119) <OG2210 .(639).
Providencia rettgeri .(1280) OG2210> .(2069) <OG12914 OG6513> .(460).
Providencia sneebia DSM 19967 .(287). <OG2210 .(2718)
Providencia stuartii MRSN 2154 .(2489) OG2210> .(704). <OG12914 OG6513> .(631).
Providencia vermicola .(504). OG2210> .(634). <OG12914 OG6513> .(2602)
Proteus terrae subsp. cibarius .(368). <OG12914 OG6513> .(3066)
Proteus hauseri .(1730) <OG12914 OG6513> .(1692)
Proteus mirabilis HI4320 .(3413) <OG12914 OG6513> .(151).
Xenorhabdus bovienii SS-2004 .(3533) <OG2210 .(21)..
Xenorhabdus doucetiae .(3415) <OG2210 .(29)..
Xenorhabdus hominickii .(1236) <OG6513 .(2632)
Xenorhabdus nematophila .(1408) <OG6513 .(1238) <OG2210 .(789).
Xenorhabdus poinarii G6
Plesiomonas shigelloides
Sodalis praecaptivus
Cardiobacterium hominis
Dichelobacter nodosus VCS1703A
Frischella perrara
Gallaecimonas mangrovi .(1123) <OG6513 .(2616)
Pseudohongiella spirulinae
Sedimenticola thiotaurini
Thiolapillus brandeum
Immundisolibacter cernigliae
Methylotuvimicrobium alcalip..
Methylotuvimicrobium buryate..
Methylomicrobium album BG8 .(531). OG6513> .(3212)
Methylococcus capsulatus str..
Methylomonas denitrificans .(495). OG3026> .(4043)
Methylomonas rhizoryzae .(3618) <OG3026 .(298).
Methylocaldum marinum
Steroidobacter denitrificans
Sulfuricaulis limicola .(296). OG6513> .(2445)
Sulfurifustis variabilis
Acetobacter ascendens .(246). <OG2210 .(2176)
Acetobacter oryzifermentans .(733). <OG2210 .(2093)
Acetobacter oryzoeni
Acetobacter pasteurianus 386B .(1939) <OG2210 .(534).
Acetobacter senegalensis .(1051) OG2210> .(2273)
Acidibrevibacterium fodinaqu.. .(3060) OG6513> .(507).
Acidiphilium multivorum AIU301 .(2718) OG2210> .(639).
Asaia bogorensis NBRC 16594
Gluconobacter albidus
Gluconobacter oxydans DSM 3504
Gluconobacter thailandicus
Granulibacter bethesdensis C..
Komagataeibacter hansenii

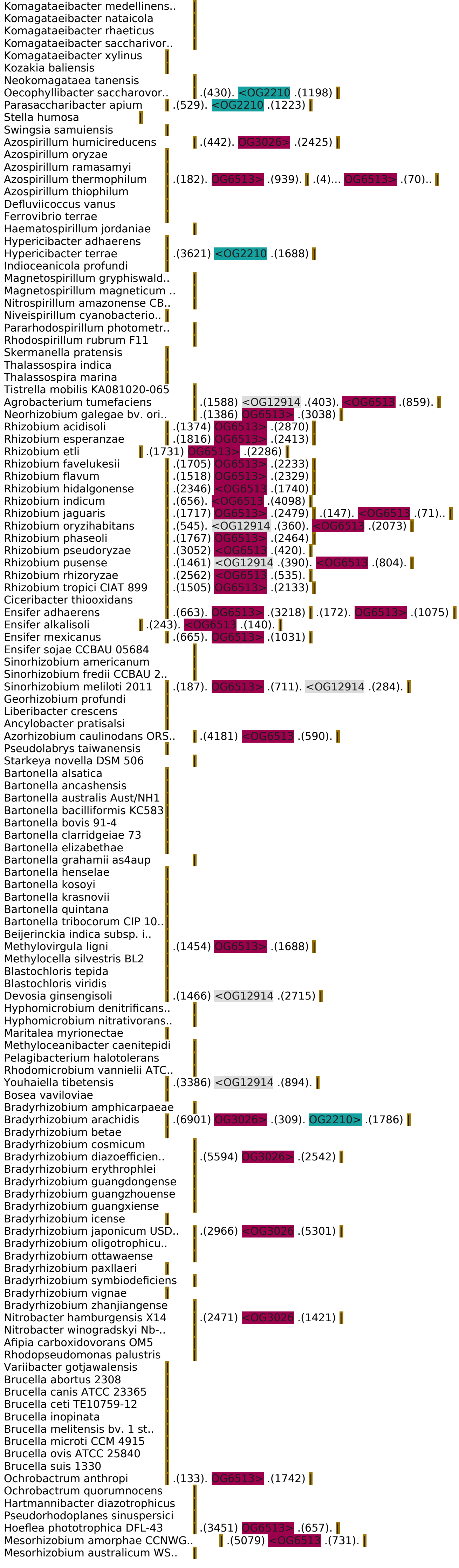
Komagataeibacter medellinens..
Komagataeibacter nataicola
Komagataeibacter rhaeticus
Komagataeibacter saccharivor..
Komagataeibacter xylinus
Kozakia baliensis
Neokomagataea tanensis
Oecophyllibacter saccharovor.. .(430). <OG2210 .(1198)
Parasaccharibacter apium .(529). <OG2210 .(1223)
Stella humosa
Swingsia samuiensis
Azospirillum humicireducens .(442). OG3026> .(2425)
Azospirillum oryzae
Azospirillum ramasamyi
Azospirillum thermophilum .(182). OG6513> .(939). .(4)... OG6513> .(70)..
Azospirillum thiophilum
Defluviicoccus vanus
Ferrovibrio terrae
Haematospirillum jordaniae
Hypericibacter adhaerens
Hypericibacter terrae .(3621) <OG2210 .(1688)
Indioceanicola profundi
Magnetospirillum gryphiswald..
Magnetospirillum magneticum ..
Nitrospirillum amazonense CB..
Niveispirillum cyanobacterio..
Pararhodospirillum photometr..
Rhodospirillum rubrum F11
Skermanella pratensis
Thalassospira indica
Thalassospira marina
Tistrella mobilis KA081020-065
Agrobacterium tumefaciens .(1588) <OG12914 .(403). <OG6513 .(859).
Neorhizobium galegae bv. ori.. .(1386) OG6513> .(3038)
Rhizobium acidisoli .(1374) OG6513> .(2870)
Rhizobium esperanzae .(1816) OG6513> .(2413)
Rhizobium etli .(1731) OG6513> .(2286)
Rhizobium favelukesii .(1705) OG6513> .(2233)
Rhizobium flavum .(1518) OG6513> .(2329)
Rhizobium hidalgonense .(2346) <OG6513 .(1740)
Rhizobium indicum .(656). <OG6513 .(4098)
Rhizobium jaguaris .(1717) OG6513> .(2479) .(147). <OG6513 .(71)..
Rhizobium oryzihabitans .(545). <OG12914 .(360). <OG6513 .(2073)
Rhizobium phaseoli .(1767) OG6513> .(2464)
Rhizobium pseudoryzae .(3052) <OG6513 .(420).
Rhizobium pusense .(1461) <OG12914 .(390). <OG6513 .(804).
Rhizobium rhizoryzae .(2562) <OG6513 .(535).
Rhizobium tropici CIAT 899 .(1505) OG6513> .(2133)
Ciceribacter thiooxidans
Ensifer adhaerens .(663). OG6513> .(3218) .(172). OG6513> .(1075)
Ensifer alkalisoli .(243). <OG6513 .(140).
Ensifer mexicanus .(665). OG6513> .(1031)
Ensifer sojae CCBAU 05684
Sinorhizobium americanum
Sinorhizobium fredii CCBAU 2..
Sinorhizobium meliloti 2011 .(187). OG6513> .(711). <OG12914 .(284).
Georhizobium profundi
Liberibacter crescens
Ancylobacter pratisalsi
Azorhizobium caulinodans ORS.. .(4181) <OG6513 .(590).
Pseudolabrys taiwanensis
Starkeya novella DSM 506
Bartonella alsatica
Bartonella ancashensis
Bartonella australis Aust/NH1
Bartonella bacilliformis KC583
Bartonella bovis 91-4
Bartonella clarridgeiae 73
Bartonella elizabethae
Bartonella grahamii as4aup
Bartonella henselae
Bartonella kosoyi
Bartonella krasnovii
Bartonella quintana
Bartonella tribocorum CIP 10..
Beijerinckia indica subsp. i..
Methylovirgula ligni .(1454) OG6513> .(1688)
Methylocella silvestris BL2
Blastochloris tepida
Blastochloris viridis
Devosia ginsengisoli .(1466) <OG12914 .(2715)
Hyphomicrobium denitrificans..
Hyphomicrobium nitrativorans..
Maritalea myrionectae
Methyloceanibacter caenitepidi
Pelagibacterium halotolerans
Rhodomicrobium vannielii ATC..
Youhaiella tibetensis .(3386) <OG12914 .(894).
Bosea vaviloviae
Bradyrhizobium amphicarpaeae
Bradyrhizobium arachidis .(6901) OG3026> .(309). OG2210> .(1786)
Bradyrhizobium betae
Bradyrhizobium cosmicum
Bradyrhizobium diazoefficien.. .(5594) OG3026> .(2542)
Bradyrhizobium erythrophlei
Bradyrhizobium guangdongense
Bradyrhizobium guangzhouense
Bradyrhizobium guangxiense
Bradyrhizobium icense
Bradyrhizobium japonicum USD.. .(2966) <OG3026 .(5301)
Bradyrhizobium oligotrophicu..
Bradyrhizobium ottawaense
Bradyrhizobium paxllaeri
Bradyrhizobium symbiodeficiens
Bradyrhizobium vignae
Bradyrhizobium zhanjiangense
Nitrobacter hamburgensis X14 .(2471) <OG3026 .(1421)
Nitrobacter winogradskyi Nb-..
Afipia carboxidovorans OM5
Rhodopseudomonas palustris
Variibacter gotjawalensis
Brucella abortus 2308
Brucella canis ATCC 23365
Brucella ceti TE10759-12
Brucella inopinata
Brucella melitensis bv. 1 st..
Brucella microti CCM 4915
Brucella ovis ATCC 25840
Brucella suis 1330
Ochrobactrum anthropi .(133). OG6513> .(1742)
Ochrobactrum quorumnocens
Hartmannibacter diazotrophicus
Pseudorhodoplanes sinuspersici
Hoeflea phototrophica DFL-43 .(3451) OG6513> .(657).
Mesorhizobium amorphae CCNWG.. .(5079) <OG6513 .(731).
Mesorhizobium australicum WS..

Mesorhizobium ciceri biovar .. .(4266) <OG6513 .(1606)
Mesorhizobium erdmanii
Mesorhizobium huakuii
Mesorhizobium jarvisii .(2366) OG6513> .(4149)
Mesorhizobium japonicum MAFF..
Mesorhizobium oceanicum
Mesorhizobium opportunistum .. .(2425) OG6513> .(4030)
Mesorhizobium terrae .(5181) OG2210> .(191).
Oricola thermophila
Phyllobacterium zundukense
Roseitalea porphyridii
Salaquimonas pukyongi
Labrenzia alexandrii DFL-11
Stappia indica .(241). OG6513> .(569). <OG12914 .(3758)
Lichenihabitans psoromatis .(3734) <OG6513 .(266). <OG12914 .(195).
Martelella endophytica
Martelella mediterranea DSM ..
Methylobacterium brachiatum
Methylobacterium currus
Methylobacterium durans
Methylobacterium mesophilicu..
Methylobacterium nodulans OR..
Methylobacterium oryzae CBMB20
Methylobacterium phyllosphae..
Methylobacterium radiotolera.. .(66).. <OG12914 .(421).
Methylobacterium terrae
Methylorubrum extorquens PA1 .(1647) OG6513> .(48).. OG6513> .(3143)
Methylorubrum populi .(310). <OG12914 .(3497) OG6513> .(1098)
Microvirga ossetica .(321). <OG12914 .(194).
Microvirga thermotolerans
Methylocystis bryophila
Methylocystis heyeri
Methylocystis parvus
Parvibaculum lavamentivorans..
Altererythrobacter atlanticus
Altererythrobacter epoxidivo.. .(1774) <OG3026 .(949).
Altererythrobacter ishigakie..
Aurantiacibacter atlanticus .(43).. <OG6513 .(2774)
Croceicoccus marinus
Erythrobacter aureus
Erythrobacter litoralis .(1146) OG6513> .(1897)
Erythrobacter mangrovi .(2548) <OG6513 .(408).
Erythrobacter neustonensis
Paraurantiacibacter namhicola
Pelagerythrobacter marensis
Qipengyuania flava
Qipengyuania sediminis
Qipengyuania seohaensis .(1020) OG3026> .(1837)
Tsuneonella amylolytica
Tsuneonella dongtanensis
Tsuneonella mangrovi
Blastomonas fulva
Novosphingobium aromaticivor.. .(1644) OG6513> .(1722)
Novosphingobium ginsenosidim.. .(103). OG6513> .(2927)
Novosphingobium pentaromativ..
Parasphingopyxis algicola
Rhizorhabdus dicambivorans
Sphingopyxis alaskensis RB2256
Sphingopyxis fribergensis
Sphingopyxis lindanitolerans
Sphingopyxis macrogoltabida
Sphingomonas alpina
Sphingomonas daechungensis
Sphingomonas ginsengisoli An..
Sphingomonas hengshuiensis
Sphingomonas lacunae
Sphingomonas lutea
Sphingomonas melonis
Sphingomonas paucimobilis
Sphingomonas panacis
Sphingomonas rhizophila
Sphingomonas sanxanigenens D.. .(4904) <OG6513 .(768).
Sphingomonas sediminicola
Sphingomonas taxi
Sphingomonas wittichii RW1
Sphingobium barthaii .(152). OG6513> .(823).
Sphingobium cloacae
Sphingobium herbicidovorans
Sphingobium hydrophobicum
Sphingobium indicum B90A
Sphingobium japonicum UT26S .(2380) <OG6513 .(916).
Allosphingosinicella indica
Sphingorhabdus lacus .(1848) <OG3026 .(1385)
Tardibacter chloracetimidivo.. .(1182) <OG3026 .(2214)
Zymomonas mobilis subsp. mob.. .(1665) OG3026> .(62)..
Sphingosinicella microcystin..
Anaplasma centrale str. Israel
Anaplasma marginale str. Flo..
Anaplasma ovis str. Haibei
Anaplasma phagocytophilum st..
Anaplasma platys
Ehrlichia canis str. Jake
Ehrlichia chaffeensis str. W..
Ehrlichia muris AS145
Ehrlichia ruminantium
Neorickettsia findlayensis
Neorickettsia helminthoeca s..
Neorickettsia risticii str. ..
Neorickettsia sennetsu str. ..
Wolbachia pipientis
Orientia tsutsugamushi .(1592) <OG3026 .(73)..
Rickettsia akari str. Hartford
Rickettsia asiatica
Rickettsia australis str. Cu..
Rickettsia conorii str. Mali..
Rickettsia helvetica C9P9
Rickettsia heilongjiangensis
Rickettsia japonica
Rickettsia monacensis
Rickettsia raoultii
Rickettsia rickettsii str. I..
Rickettsia sibirica 246
Rickettsia slovaca 13-B
Rickettsia bellii RML369-C
Rickettsia canadensis str. C..
Rickettsia prowazekii str. C..
Rickettsia typhi str. TH1527
Asticcacaulis excentricus CB..
Brevundimonas subvibrioides ..
Brevundimonas vancanneytii
Brevundimonas vesicularis
Caulobacter flavus
Caulobacter mirabilis
Caulobacter rhizosphaerae
Caulobacter segnis
Caulobacter vibrioides NA1000

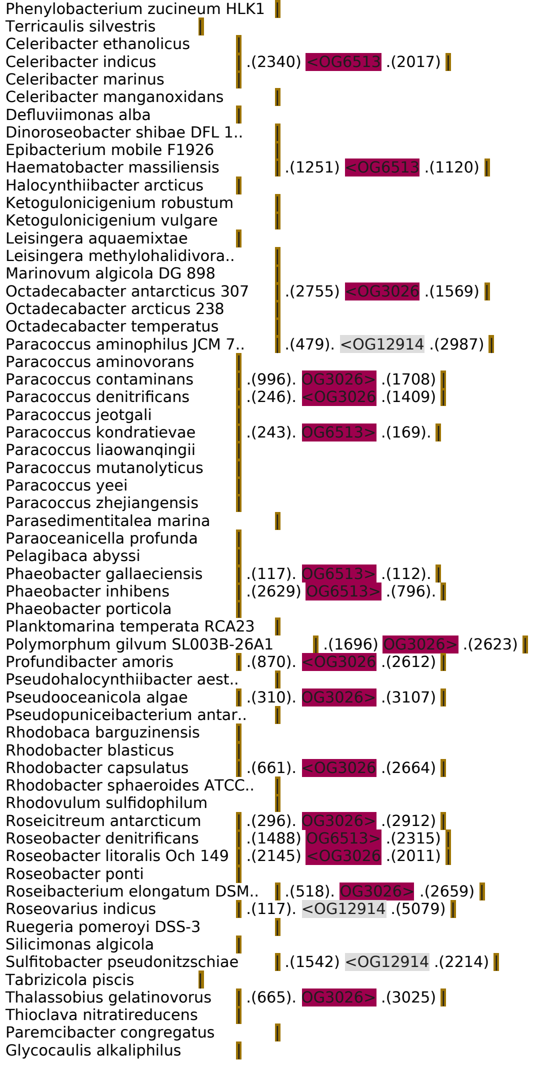
Phenylobacterium zucineum HLK1
Terricaulis silvestris
Celeribacter ethanolicus
Celeribacter indicus .(2340) <OG6513 .(2017)
Celeribacter marinus
Celeribacter manganoxidans
Defluviimonas alba
Dinoroseobacter shibae DFL 1..
Epibacterium mobile F1926
Haematobacter massiliensis .(1251) <OG6513 .(1120)
Halocynthiibacter arcticus
Ketogulonicigenium robustum
Ketogulonicigenium vulgare
Leisingera aquaemixtae
Leisingera methylohalidivora..
Marinovum algicola DG 898
Octadecabacter antarcticus 307 .(2755) <OG3026 .(1569)
Octadecabacter arcticus 238
Octadecabacter temperatus
Paracoccus aminophilus JCM 7.. .(479). <OG12914 .(2987)
Paracoccus aminovorans
Paracoccus contaminans .(996). OG3026> .(1708)
Paracoccus denitrificans .(246). <OG3026 .(1409)
Paracoccus jeotgali
Paracoccus kondratievae .(243). OG6513> .(169).
Paracoccus liaowanqingii
Paracoccus mutanolyticus
Paracoccus yeei
Paracoccus zhejiangensis
Parasedimentitalea marina
Paraoceanicella profunda
Pelagibaca abyssi
Phaeobacter gallaeciensis .(117). OG6513> .(112).
Phaeobacter inhibens .(2629) OG6513> .(796).
Phaeobacter porticola
Planktomarina temperata RCA23
Polymorphum gilvum SL003B-26A1 .(1696) OG3026> .(2623)
Profundibacter amoris .(870). <OG3026 .(2612)
Pseudohalocynthiibacter aest..
Pseudooceanicola algae .(310). OG3026> .(3107)
Pseudopuniceibacterium antar..
Rhodobaca barguzinensis
Rhodobacter blasticus
Rhodobacter capsulatus .(661). <OG3026 .(2664)
Rhodobacter sphaeroides ATCC..
Rhodovulum sulfidophilum
Roseicitreum antarcticum .(296). OG3026> .(2912)
Roseobacter denitrificans .(1488) OG6513> .(2315)
Roseobacter litoralis Och 149 .(2145) <OG3026 .(2011)
Roseobacter ponti
Roseibacterium elongatum DSM.. .(518). OG3026> .(2659)
Roseovarius indicus .(117). <OG12914 .(5079)
Ruegeria pomeroyi DSS-3
Silicimonas algicola
Sulfitobacter pseudonitzschiae .(1542) <OG12914 .(2214)
Tabrizicola piscis
Thalassobius gelatinovorus .(665). OG3026> .(3025)
Thioclava nitratireducens
Paremcibacter congregatus
Glycocaulis alkaliphilus

Supplementary data 2: The microsynteny analysis of AS_1503 and its orthologs. The colored boxes represent the five genes (OG12704: WP_001046931.1, hypothetical protein; OG2210: WP_000378375.1, cytosine permease; OG12914: WP_001161759.1, AS_1503, OG6513: WP_001010546.1, LysR family transcriptional regulator; OG3026: WP_000108023.1, Integrase), and focal gene AS_1503 is shown in white. Vertical bars represent config/scaffold borders, and numbers in parenthesis give the number of intervening genes between two detected orthologs.
